# Design, synthesis, and insecticidal potency of novel 3-methyl-pyrazole derivatives against *Culex pipiens* larvae

**DOI:** 10.1038/s41598-026-50895-3

**Published:** 2026-05-09

**Authors:** Hager R. Nofal, Ali Khalil Ali, Mahmoud F. Ismail, Mahmoud Kamal, Eslam M. Hosni, Eslam M. Abbass

**Affiliations:** 1 Chemistry Department, Faculty of Science, Ain Shams University, Cairo, 11566 Egypt; 2 Entomology Department, Faculty of Science, Ain Shams University, Cairo, 11566 Egypt

**Keywords:** *Culex pipiens*, 3-Methyl-pyrazole, Larvicidal activity, Acetylcholinesterase inhibition, Molecular docking, SAR, Mosquito control, Biochemistry, Chemical biology, Chemistry, Computational biology and bioinformatics, Drug discovery

## Abstract

Mosquito-borne illnesses remain a serious public health concern worldwide, with the growing resistance of vectors such as *Culex pipiens* reducing the efficacy of standard insecticides. In pursuit of alternative control strategies, a new library of 3-methyl-pyrazole derivatives was rationally designed, synthesized through multistep reactions, and comprehensively characterized using IR, NMR, and mass spectrometry. Larvicidal screening of all nineteen synthesized 2-methyl-pyrazole derivatives, conducted according to WHO protocols, revealed several highly active candidates. Among these, derivatives **7** and **12** exhibited the most potent activity, with LC_50_ values of 0.16 µg/mL and 0.30 µg/mL, respectively — far surpassing the performance of the reference insecticide chlorpyrifos (LC_50_ = 52.90 µg/mL). Complementary in silico analyses, including molecular docking and molecular dynamics (MD) simulations, were employed to elucidate the mode of action of the synthesized compounds. Docking studies were performed for all nineteen 3-methyl-pyrazole derivatives against two principal neural targets: acetylcholinesterase (AChE), the enzyme responsible for acetylcholine degradation, and the nicotinic acetylcholine receptor (nAChR), a major ligand-gated ion channel involved in synaptic transmission. To further examine binding stability, MD simulations were conducted only for the two most active derivatives (**7** and **12**) in complex with AChE, using chlorpyrifos as a reference ligand. The simulations confirmed that the active compounds formed stable and persistent interactions within the catalytic site, consistent with a neurotoxic mechanism involving inhibition of AChE. Structure–activity relationship (SAR) analysis further identified functional groups contributing to potency. Overall, this preliminary investigation establishes a computational and experimental foundation for future optimization, toxicological validation, and eventual field application of next-generation larvicides designed to mitigate resistance and control mosquito-borne diseases.

**Supplementary Information:**

The online version contains supplementary material available at 10.1038/s41598-026-50895-3.

## Introduction

The common house mosquito *Culex pipiens* is a significant vector of concern in public health due to its capability to transmit a variety of serious and sometimes fatal diseases. This species is a carrier of several arboviruses, notably West Nile virus, Usutu virus, and St. Louis encephalitis virus, all of which contribute substantially to the global spread of vector-borne illnesses^1–3^. Additionally, *C. pipiens* can transmit filarial nematodes responsible for canine dirofilariasis and plays a major role in spreading *Plasmodium* parasites that cause avian malaria^4^. Recent studies have indicated that raw milk may be contaminated through the transmission of pathogenic bacteria linked to serious diseases, posing an additional risk to food safety and public health^5^. These characteristics underline the urgent need to focus control strategies on this mosquito species.

Historically, chemical insecticides have been the primary choice for managing *C. pipiens* populations. However, the widespread and repeated application of these agents has accelerated the emergence of resistance, reducing their overall effectiveness in disease control^6,7^. Resistance is not confined to a single mosquito species; numerous mosquito populations worldwide have developed mechanisms to withstand common insecticides, necessitating continuous research into novel chemical agents and integrated pest management strategies^7–14^. Compounding the issue, climate change is altering the geographical distribution of insects, enabling mosquitoes to invade new areas and facilitating the spread of vector-borne diseases into regions previously unaffected^9–21^. Despite these challenges, chemical insecticides remain an important short-term intervention due to their rapid action, which is vital during outbreak situations to reduce pathogen transmission. Long-term solutions, however, must incorporate sustainable approaches aimed at delaying or preventing resistance development^22–24^.

Responding to these challenges, the current work involves the creation and biological evaluation of nineteen novel 3-methyl-pyrazole derivatives specifically developed to target *Culex pipiens* larvae. The selection of acetylcholinesterase (AChE) and nicotinic acetylcholine receptor (nAChR) as molecular targets was guided by the structural features and functional groups of the synthesized compounds, which resemble those present in major neuroactive insecticides such as organophosphates, carbamates, and neonicotinoids. Most commercial insecticides exert their toxicity through interference with neural signal transmission involving four principal molecular targets, AChE, nAChR, GABA receptors, and voltage-gated sodium channels, with AChE and nAChR being the most frequently affected^25–32^. Based on this well-established neurotoxic framework, the two targets were rationally selected for in silico assessment to evaluate whether the new pyrazole derivatives engage them in a similar manner to reference insecticides. Molecular docking analyses were therefore performed for all nineteen synthesized 3-methyl-pyrazole derivatives to investigate their binding affinities toward AChE and nAChR. To gain deeper insight into binding stability and dynamic behavior, molecular dynamics (MD) simulations were subsequently conducted for selected compounds, particularly those exhibiting the highest larvicidal activity, in complex with AChE, using chlorpyrifos as a reference inhibitor.

Through this integrated experimental–computational approach, the present work aims to establish a solid scientific foundation for the rational design of next-generation, environmentally safer, and resistance-mitigating larvicides. While *Culex pipiens* was used as a representative vector species in this preliminary stage, it is recommended that future studies extend the biological evaluation to include additional mosquito genera such as *Aedes* and *Anopheles*, thereby enabling cross-species validation and improving the translational potential of these findings toward practical vector control applications.

## Experimental

### Chemistry

Thin-layer chromatography (TLC) on UV-fluorescent silica gel plates (Merck 60 F_254_) was employed to monitor the progress of the reactions. Melting points of the synthesized compounds were determined using a Gallenkamp apparatus and are reported without correction. Fourier-transform infrared (FT-IR) spectra were recorded on a Nicolet iS10 spectrometer. Proton nuclear magnetic resonance (^1^H NMR) spectra were obtained on a JEOL ECA-500 instrument (Shimadzu) using DMSO-d₆ as the solvent and tetramethylsilane (TMS) as an internal standard. Elemental composition was determined with a Perkin-Elmer 2400 CHN analyzer. Mass fragmentation patterns were acquired using a Shimadzu GC–MS QP1000EX system.

#### Ethyl − 3-(2-carbamothioylhydrazineylidene)butanoate (1)

A mixture of ethyl acetoacetate (1.3 mL, 0.01 mol) and thiosemicarbazide (0.92 g, 0.01 mol) was dissolved in absolute ethanol (25 mL). A few drops of acetic acid were added, and the solution was heated under reflux for 8 h. TLC checked the progress of the reaction. After completion, the mixture was left to evaporate in a beaker. The resulting precipitate was collected by filtration, rinsed repeatedly with ethanol, dried, and purified through recrystallization to yield compound **1** as white crystals.

#### 3-methyl-5-oxo-4,5-dihydro-1 H-pyrazole-1-carbothioamide (2)

##### Method (a)

Compound 1 (2.03 g, 0.01 mol) was combined with fused sodium acetate (0.82 g, 0.02 mol) in absolute ethanol (25 mL). The mixture was refluxed for 10 h, and the course of reaction was followed by TLC. Upon completion, the hot solution was poured into ice-cold water and acidified with HCl. The solid that separated was filtered, washed thoroughly with water, dried, and recrystallized from benzene, affording compound **2** as white crystals (m.p.: 178–180 °C, yield: 40%).

##### Method (b)

A mixture of **1** (2.03 g, 0.01 mol), fused sodium acetate (0.82 g, 0.02 mol), and a few milligrams (0.01 g) of TiO_2_ nanoparticles in absolute ethanol (25 mL) was refluxed for 10 h. TLC monitored the reaction. Upon completion, the hot solution was poured into ice-cold water and acidified with HCl. The solid that separated was filtered, washed thoroughly with water, dried, and recrystallized from benzene, affording compound **2** as white crystals (m.p.: 178–180 °C, yield: 85%).

IR (KBr, υ/cm^− 1^): 3260, 3190 (NH_2_), 1649 (C = O). ^1^H NMR (500 MHz, DMSO-*d*_6_) δ (ppm): 17.32 (br. s, 1H, OH), 12.22 (br. s, 2 H, NH_2_), 7.36 (s, 1H, CH), 2.22 (s, 3 H, CH_3_). Anal. Calcd for: C_5_H_7_N_3_OS (157.19): C, 38.21; H, 4.49; N, 26.73; Found: C, 38.42; H, 4.62; N, 26.89.

#### 5-methyl-2-(4-phenylthiazol-2-yl)-2,4-dihydro-3 H-pyrazol-3-one (3)

Compound 1 (2.03 g, 0.01 mol) was dissolved in ethanol (25 mL) along with phenacyl bromide (1.99 g, 0.01 mol). The reaction mixture was heated under reflux for 6 h, after which it was poured into ice-cold water. The precipitated solid was collected by filtration, rinsed thoroughly with water, dried, and obtained as the intermediate product. This intermediate (3.03 g, 0.01 mol) was then combined with fused sodium acetate (0.82 g, 0.03 mol) in ethanol (25 mL) and refluxed for 24 h. TLC monitored reaction progress. On completion, the solution was poured into cold water and acidified with HCl. The resulting solid was filtered, washed with water, dried, and recrystallized from petroleum ether, yielding compound **3** as orange crystals. m.p.: 180–184 °C, yield: 78%. IR (KBr, υ/cm^− 1^): 3112 (C-H_arom_), 3026 (C-H_aliph_), 1634 (C = O). Anal. Calcd for: C_13_H_11_N_3_OS (257.31): C, 60.68; H, 4.31; N, 16.33; Found: C, 60.85; H, 4.52; N, 16.55.

#### 6-amino-5-cyano-4-(4-methoxyphenyl)-3-methylpyrano[2,3-c]pyrazole-1(4 H)-carbothioamide (4)

A mixture of compound **2** (1.57 g, 0.01 mol), *p*-anisaldehyde (1.36 mL, 0.01 mol), and malononitrile (0.66 g, 0.01 mol) in ethanol (25 mL) was treated with a few drops of piperidine. The solution was refluxed for 15 h while being monitored by TLC. After completion, the mixture was poured into ice-cold water and acidified with HCl. The precipitate that formed was filtered, washed with water, dried, and recrystallized from benzene to afford compound **4** as orange crystals. m.p.: 150–154 °C, yield: 27%. IR (KBr, υ/cm^− 1^): 3333, 3216 (NH_2_), 3070 (C-H_arom_), 2839 (C-H_aliph_), 2202 (C ≡ N).^1^H NMR (500 MHz, DMSO-*d*_6_) δ (ppm): 7.44 (d, 2 H, Ar-H), 7.03 (d, 2 H, Ar-H), 6.88 (br. s, 2 H, NH_2_), 6.87 (br. s, 2 H, NH_2_), 3.83 (s, 1H, CH), 3.72 (s, 3 H, OCH_3_), 1.62 (s, 3 H, CH_3_). MS (*m*/*z*) (%): 341.24 (M^+^., 15), 146.16 (100). Anal. Calcd for: C_16_H_15_N_5_O_2_S (341.39): C, 56.29; H, 4.43; N, 20.51; Found: C, 56.42; H, 4.58; N, 20.72.

### General procedure for synthesis of compounds 5, 6 and 7

A solution of compound **2** (1.57 g, 0.01 mol), *p*-anisaldehyde (1.36 mL, 0.01 mol) or *p*-(N, N-dimethylamino)benzaldehyde (1.49 g, 0.01 mol) or *p*-nitrobenzaldehyde (1.51 g, 0.01 mol), together with malononitrile (0.66 g, 0.01 mol) and ammonium acetate (0.5 g), was prepared in ethanol (25 mL). The mixture was heated under reflux for 10–15 h, and the progress was monitored by TLC. After completion, the reaction was poured into ice-cold water. The solid product was isolated by filtration, washed thoroughly with water, dried, and crystallized to afford compounds **5**, **6**, and **7**, respectively.

#### 6-amino-5-cyano-4-(4-methoxyphenyl)-3-methyl-1 H-pyrazolo[3,4-b]pyridine-1-carbothioamide (5)

Compound **5** was obtained as orange crystals after recrystallization from ethanol, m.p.: 150–154 °C, yield: 24%. IR (KBr, υ/cm^− 1^): 3476, 3363 (NH_2_), 3215 (NH_2_.), 3142 (C-H_arom_.), 2202 (CN). ^1^H NMR (500 MHz, DMSO-*d*_6_) δ (ppm): 7.44 (d, 2 H, Ar-H), 7.16 (d, 2 H, Ar-H), 7.09 (br. s, 2 H, NH_2_), 7.07(br. s, 2 H, NH_2_), 3.83 (s, 3 H, OCH_3_), 2.4 (s, 3 H, CH_3_). MS (*m*/*z*) (%): 338.52 (M^+^., 26), 284.53 (100). Anal. Calcd for: C_16_H_14_N_6_OS (338.39): C, 56.79; H, 4.17; N, 24.84; Found: C, 56.95; H, 4.29; N, 25.06.

#### 6-amino-5-cyano-4-(4-(dimethylamino)phenyl)-3-methyl-1 H-pyrazolo[3,4-b]pyridine-1-carbothioamide (6)

Compound **6** was obtained as orange crystals after recrystallization from benzene, m.p.: 218–220 °C, yield: 15%. IR (KBr, υ/cm^− 1^): 3346, 3204 (NH_2_), 3100 (C-H_arom_.), 2203 (CN.). ^1^H NMR (500 MHz, DMSO-*d*_6_) δ (ppm): 8.57 (br. s, 2 H, NH_2_), 7.42 (d, 2 H, Ar-H), 7.22 (d, 2 H, Ar-H), 7.07(br. s, 2 H, NH_2_), 3.11(s, 3 H, CH_3_), 3.01(s, 3 H, CH_3_), 2.12(s, 3 H, CH_3_). MS (*m*/*z*) (%): 351.48 (M^+^., 27), 66.26 (100). Anal. Calcd for: C_17_H_17_N_7_S (351.43): C, 58.10; H, 4.88; N, 27.90; Found: C, 58.26; H, 5.05; N, 28.12.

#### 6-amino-5-cyano-3-methyl-4-(4-nitrophenyl)-1 H-pyrazolo[3,4-b]pyridine-1-carbothioamide (7)

Compound **7** was obtained as brown crystals after recrystallization from ethanol, m.p.: 276–280 °C, yield: 18%. IR (KBr, υ/cm^− 1^): 3458, 3347 (NH_2_), 3100 (C-H_arom_.), 2209 (CN.). ^1^H NMR (500 MHz, DMSO-*d*_6_) δ (ppm): 11.34 (s, 2 H, NH_2_), 8.09 (d, 2 H, Ar-H), 7.36 (d, 2 H, Ar-H), 4.94(s, 2 H, NH_2_), 2.06(s, 3 H, CH_3_). MS (*m*/*z*) (%): 353.56 (M^+^., 15), 95.10 (100). Anal. Calcd for: C_15_H_11_N_7_O_2_S (353.36): C, 50.99; H, 3.14; N, 27.75; Found: C, 51.21; H, 3.29; N, 27.94.

### General procedure for synthesis of compounds 8 and 9

Compound **2** (1.57 g, 0.01 mol) was mixed with *p*-anisaldehyde (1.36 mL, 0.01 mol) or p-nitrobenzaldehyde (1.51 g, 0.01 mol) in fused sodium acetate (0.82 g, 0.01 mol) using acetic acid (20 mL) as solvent. The reaction mixture was refluxed for 8 h, with progress monitored by TLC. After completion, the mixture was poured into ice-cold water. The resulting precipitate was filtered, washed thoroughly with water, dried, and crystallized to afford compounds **8** and **9**, respectively.

#### 4-(4-methoxybenzylidene)-3-methyl-5-oxo-4,5-dihydro-1 H-pyrazole-1-carbothioamide (8)

Compound **8** was isolated as yellow crystals after recrystallization from ethanol, m.p.: 150–154 °C, yield: 25%. IR (KBr, υ/cm^− 1^): 3200 (br.) (NH_2_), 2836 (C-H_arom_.), 2724 (C-H_aliph_.), 1730 (C = O _(ketone)_). ^1^H NMR (500 MHz, DMSO-*d*_6_) δ (ppm): 7.04 (d, 2 H, Ar-H), 7.02 (d, 2 H, Ar-H), 6.99(s, 1H, CH), 6.76(s, 2 H, NH_2_), 3.62(s, 3 H, OCH_3_), 2.12(s, 3 H, CH_3_). MS (*m*/*z*) (%): 375.41 (M^+^., 59), 64.12 (100). Anal. Calcd for: C_13_H_13_N_3_O_2_S (375.33): C, 56.71; H, 4.76; N, 15.26; Found: C, 56.89; H, 4.86; N, 15.42.

#### 3-methyl-4-(4-nitrobenzylidene)-5-oxo-4,5-dihydro-1 H-pyrazole-1-carbothioamide (9)

Compound 9 was obtained as orange crystals after recrystallization from benzene, m.p.: 226–230 °C, yield: 14%. IR (KBr, υ/cm^− 1^): 3250 (br.) (NH_2_), 2926 (C-H_arom_.), 2865 (C-H_aliph_.), 1702 (C = O _(ketone)_). ^1^H NMR (500 MHz, DMSO-*d*_6_) δ (ppm): 8.69(s, 1H, CH), 8.58(d, 2 H, Ar-H), 8.34(d, 2 H, Ar-H), 7.96(br. s, 2 H, NH_2_), 2.27(s, 3 H, CH_3_). MS (*m*/*z*) (%): 290.81 (M^+^., 37), 83.62 (100). Anal. Calcd for: C_12_H_10_N_4_O_3_S (290.05): C, 49.65; H, 3.47; N, 19.30; Found: C, 49.82; H, 3.62; N, 19.50.

### General procedure for synthesis of compounds 10, 11, 12 and 13

A mixture of 2-aminothiazole (1 g, 0.01 mol) or *p*-toluidine or *p*-nitroaniline or *p*-hydroxyaniline and sodium nitrite (0.69 g, 0.01 mol) in HCl (10 mL, 5 M) was stirred for 20 min. to make diazonium salt in ultrasonic put **2** (1.57 g, 0.01 mol) dissolved in acetone and drops of sodium hydroxide to make a basic solution, then add on diazonium salt drop by drop in the ice bath and stir for 4 h. The reaction was monitored by TLC. The resulting precipitate was filtered off, washed several times with water, thoroughly dried, and crystallized to give compound **10**, **11**, **12** and **13**, respectively.

#### 3-methyl-5-oxo-4-(thiazol-2-yldiazenyl)-4,5-dihydro-1 H-pyrazole-1-carbothioamide (10)

Purified by recrystallization from EtOH to afford **10** as red crystals m.p.: 238–240 °C, yield: 23%. IR (KBr, υ/cm^− 1^): 3412, 3297 (NH, NH_2_), 3146 (C-H_arom_.), 2958 (C-H_aliph_.), 1652 (C = O _(ketone)_). ^1^H NMR (500 MHz, DMSO-*d*_6_) δ (ppm): 10.39(s, 1H, NH), 8.94(br. s, 2 H, NH_2_), 7.58(s, 1H, C_5_ H), 7.21(s, 1H, C_4_ H), 2.31(s, 3 H, CH_3_). MS (*m*/*z*) (%): 268.49 (M^+^., 47), 95.25 (100). Anal. Calcd for: C_8_H_8_N_6_OS_2_ (268.31): C, 35.81; H, 3.01; N, 31.32; Found: C, 35.97; H, 3.18; N, 31.53.

#### 3-methyl-5-oxo-4-(2-(p-tolyl)hydrazineylidene)-4,5-dihydro-1 H-pyrazole-1-carbothioamide (11)

Purified by recrystallization from EtOH to afford **11** as orange crystals m.p.: 220–222 °C, yield: 48%. IR (KBr, υ/cm^− 1^): 3432, 3262 (NH, NH_2_), 3158 (C-H_arom_.), 2921 (C-H_aliph_.), 1689 (C = O _(ketone)_). ^1^H NMR (500 MHz, DMSO-*d*_6_) δ (ppm): 9.41 (s, 1H, NH), 8.95 (br. s, 2 H, NH_2_), 7.54 (d, 2 H, Ar-H), 7.34 (d, 2 H, Ar-H), 2.31 (s, 3 H, CH_3_), 2.25 (s, 3 H, CH_3_). MS (*m*/*z*) (%): 275.14 (M^+^., 30), 213.21 (100). Anal. Calcd for: C_12_H_13_N_5_OS (275.33): C, 52.35; H, 4.76; N, 25.44; Found: C, 52.48; H, 4.92; N, 25.67.

#### 3-methyl-4-((4-nitrophenyl)diazenyl)-5-oxo-4,5-dihydro-1 H-pyrazole-1-carbothioamide (12)

Purified by recrystallization from EtOH to afford **12** as orange crystals m.p.: 238–240 °C, yield: 41%. IR (KBr, υ/cm^− 1^): 3394, 3288 (NH, NH_2_), 3101 (C-H_arom_.), 2925 (C-H_aliph_.), 1710 (C = O _(ketone)_), (1335, 1581) (NO_2_). Anal. Calcd for: C_11_H_10_N_6_O_3_S (306.30): C, 43.13; H, 3.29; N, 27.44; Found: C, 43.27; H, 3.48; N, 27.61.

#### 4-((4-hydroxyphenyl)diazenyl)-3-methyl-5-oxo-4,5-dihydro-1 H-pyrazole-1-carbothioamide (13)

Purified by recrystallization from EtOH to afford **13** as brown crystals m.p.: 225–227 °C, yield: 20%. IR (KBr, υ/cm^− 1^): 3257 (broad OH), 3200 (br.) (NH, NH_2_), 3105 (C-H_arom_.), 1648 (C = O _(ketone)_). Anal. Calcd for: C_11_H_11_N_5_O_2_S (277.30): C, 47.65; H, 4.00; N, 25.26; Found: C, 47.81; H, 4.16; N, 25.39.

#### 4-amino-5-imino-2-methyl-5,6-dihydro-7 H-3-thia-1,6,7a-triazacyclopenta[cd]indene-7-thione (14)

Compound **2** (1.57 g, 0.01 mol), malononitrile (0.66 g, 0.01 mol), and elemental sulfur (0.32 g, 0.01 mol) were dissolved in ethanol (25 mL). A few drops of morpholine were added, and the mixture was stirred at 60 °C for 4 h. The progress was monitored by TLC. After completion, the reaction mixture was poured into ice-cold water and acidified with HCl. The precipitate was collected by filtration, washed thoroughly with water, dried, and recrystallized from benzene to afford compound **14** as yellow crystals. m.p.: 170–176 °C, yield: 26%. IR (KBr, υ/cm^− 1^): 3269, 3201, 3141 (NH, NH_2_), 2904 (C-H_aliph_), 1202 (C = S). ^1^H NMR (500 MHz, DMSO-*d*_6_) δ (ppm): 11.34 (s, 1H, NH, exchangeable with D_2_O), 8.73 (s, 1H, NH, exchangeable with D_2_O), 6.57 (br. s, 1H, NH_2_, exchangeable with D_2_O), 2.3 (s, 3 H, CH_3_). MS (*m*/*z*) (%): 237.41 (M^+^., 20), 75.26 (100). Anal. Calcd for: C_8_H_7_N_5_S_2_ (237.30): C, 40.49; H, 2.97; N, 29.51; Found: C, 40.59; H, 3.18; N, 29.72.

#### 1-(benzoylcarbamothioyl)-3-methyl-1 H-pyrazol-5-yl benzoate (15)

Compound **2** (1.57 g, 0.01 mol) was treated with benzoyl chloride (1.4 mL, 0.01 mol) and potassium hydroxide (0.56 g, 0.01 mol) in dioxane (25 mL). The mixture was refluxed for 10 h with monitoring by TLC. After completion, the hot solution was poured into ice-cold water and acidified with HCl. The separated solid was filtered, washed with water, dried, and recrystallized from petroleum ether to give compound **15** as grey crystals. m.p.: 96–98 °C, yield: 10%. IR (KBr, υ/cm^− 1^): 3416 (NH), 2959 (C-H_arom_.), 1748 (C=O_ester_), 1707 (C=O_amide_). ^1^H NMR (500 MHz, DMSO-*d*_6_) δ (ppm): 8.09 (d, 2 H, Ar-H), 7.87 (d, 2 H, Ar-H), 7.75 (t, 1H, Ar-H), 7.68 (s, 1H, CH_olefinic_), 7.66–7.51 (m, 5 H, Ar-H), 6.58 (s, 1H, NH), 2.66 (s, 3 H, CH_3_). MS (*m*/*z*) (%): 365.81 (M^+^., 27), 264.12 (100). Anal. Calcd for: C_19_H_15_N_3_O_3_S (365.41): C, 62.45; H, 4.14; N, 11.50; Found: C, 62.61; H, 4.28; N, 11.72.

#### 6-amino-4-(4-methoxyphenyl)-3-methyl-1-(4-phenylthiazol-2-yl)-1 H-pyrazolo[3,4-b]pyridine-5-carbonitrile (16)

Compound **3** (2.57 g, 0.01 mol), *p*-anisaldehyde (1.36 mL, 0.01 mol), and malononitrile (0.66 g, 0.01 mol) were dissolved in ethanol (25 mL). Ammonium acetate (0.5 g) was added, and the mixture was refluxed for 8 h with TLC monitoring. After completion, the reaction was poured into ice-cold water. The product was filtered, washed with water, dried, and recrystallized from benzene to give compound **16** as brown crystals. m.p.: 218–220 °C, yield: 79%. IR (KBr, υ/cm^− 1^): 3366 (br.) (NH_2_), 3184 (C-H_arom_.), 2206 (C ≡ N.). ^1^H NMR (500 MHz, DMSO-*d*_6_) δ (ppm): 7.97–7.12 (m, 9 H, Ar-H), 7.07 (br. s, 2 H, NH_2_), 6.9 (s, 1H, CH_thiazole_), 3.86 (s, 3 H, OCH_3_), 2.27 (s, 3 H, CH_3_). MS (*m*/*z*) (%): 437.67 (M^+^., 34), 145.12 (100). Anal. Calcd for: C_24_H_18_N_6_OS (438.51): C, 65.74; H, 4.14; N, 19.17; Found: C, 65.91; H, 4.28; N, 19.31.

#### 4-(4-methoxybenzylidene)-5-methyl-2-(4-phenylthiazol-2-yl)-2,4-dihydro-3 H-pyrazol-3-one (17)

Compound **3** (2.57 g, 0.01 mol) and *p*-anisaldehyde (1.36 mL, 0.01 mol) were dissolved in acetic acid in the presence of fused sodium acetate. The mixture was refluxed for 8 h, with progress monitored by TLC. On completion, the hot solution was poured into ice-cold water, and the precipitate was collected by filtration, washed thoroughly with water, dried, and recrystallized from petroleum ether to give compound **17** as orange crystals. m.p.: 256–260 °C, yield: 68%. IR (KBr, υ/cm^− 1^): 3061 (C-H_arom_.), 2924 (C-H_aliph_.), and 1638 (C = O _(ketone)_). ^1^H NMR (500 MHz, DMSO-*d*_6_) δ (ppm): 8.71 (s, 1H, CH_(olefinic)_), 8.69–7.14 (m, 9 H, Ar-H), 6.94 (s, 1H, CH_(thiazole)_), 3.92 (s, 3 H, OCH_3_), 2.42 (s, 3 H, CH_3_). MS (*m*/*z*) (%): 375.03 (M^+^., 25), 55.39 (100). Anal. Calcd for: C_21_H_17_N_3_O_2_S (375.45): C, 67.18; H, 4.56; N, 11.19; Found: C, 67.32; H, 4.68; N, 11.37.

#### 5-amino-3-methyl-1-(4-phenylthiazol-2-yl)-1 H-thieno[3,2-c]pyrazole-6-carbonitrile (18)

Compound **3** (2.57 g, 0.01 mol), malononitrile (0.66 g, 0.01 mol), and elemental sulfur (0.32 g, 0.01 mol) were dissolved in ethanol (25 mL). A few drops of morpholine were added, and the mixture was stirred at 60 °C for 4 h while monitoring by TLC. After completion, the mixture was poured into ice-cold water, acidified with HCl, and the precipitate was filtered, washed thoroughly with water, dried, and recrystallized from ethanol to yield compound **18** as brown crystals. m.p.: 198–200 °C, yield: 57%. IR (KBr, υ/cm^− 1^): 3327 (br.) (NH_2_), 3109 (C-H_arom_), 2964 (C-H_aliph_), 2201 (C ≡ N). ^1^H NMR (500 MHz, DMSO-*d*_6_) δ (ppm): 8.00 (s, 1H, CH_thiazole_), 7.93–7.29 (m, 5 H, Ar-H), 3.75(s, 2 H, NH_2_, exchangeable with D_2_O), 2.27 (s, 3 H, CH_3_). MS (*m*/*z*) (%): 337.70 (M^+^., 38), 206.24 (100). Anal. Calcd for: C_16_H_11_N_5_S_2_ (337.42): C, 56.95; H, 3.29; N, 20.76; Found: C, 57.08; H, 3.38; N, 20.91.

#### 5-methyl-2-(4-phenylthiazol-2-yl)-4-(p-tolyldiazenyl)-2,4-dihydro-3 H-pyrazol-3-one (19)

A mixture of *p*-hydroxyaniline (1.09 g, 0.01 mol), and sodium nitrite (0.69 g, 0.01 mol) in HCl (10 ml, 5 M) was stirred for 20 min. to make diazonium salt. in ultrasonic put **3** (2.57 g, 0.01 mol), dissolved in acetone and drops of NaOH to make basic solution, then add on diazonium salt drop by drop an ice bath and make stir for 4 h. The reaction was monitored by TLC. The resulting ppt was filtered off, washed several times with water, dried well and then purified by recrystallization from benzene to afford **19** as orange crystals m.p.: over 200–204 °C, yield: 62%. IR (KBr, υ/cm^− 1^): 3420 (br.) (NH), 3061 (C-H_arom_.), 2922 (C-H_aliph_.), 1602 (br.) (C = O _(ketone)_). Anal. Calcd for: C_20_H_17_N_5_OS (375.45): C, 63.98; H, 4.56; N, 18.65; Found: C, 64.07; H, 4.68; N, 18.77.

### Biological activity

#### Mosquito rearing

A laboratory strain of *Culex pipiens* was maintained for over 40 successive generations in the insectary of the Entomology Department, Faculty of Science, Ain Shams University. The rearing environment was maintained under controlled conditions, with a temperature of 27 ± 2 °C, relative humidity set at 75%, and a light–dark cycle of 12 h each^33^. A commercial fish food (TetraMin) was used to feed the larvae. Adult females were provided with blood meals using live pigeons (*Columba livia* domestica), which were exposed to the mosquitoes for approximately 20 min per feeding. A 10% sucrose solution was available daily for adults of both sexes^34^.

#### Bioassay test

Larvicidal assays were conducted following WHO guidelines^35^. The bioassay involved exposing third-instar larvae to a series of five concentrations for each test compound (11.25, 22.5, 45, 67.5, and 90 µg/mL). Stock solutions were initially prepared in dimethylformamide (DMF) and subsequently diluted with dechlorinated water. The final DMF concentration in all test solutions was maintained below 1% (v/v), which is within the safe limit recommended by WHO and does not cause larval mortality. Control groups received DMF and water only, and the observed mortality in all control replicates remained below 5%, confirming assay validity and compliance with WHO criteria. Each concentration and control was tested in triplicate. Mortality was assessed after 24 h, with larvae classified as dead if they failed to respond to gentle prodding^36^.

#### Data analysis

Mortality data obtained from the larval bioassays were processed using the LDP line statistical software to determine the lethal concentration that kills 50% of the test population (LC_50_), along with the corresponding 95% confidence intervals (C.I.). Mortality in control groups was corrected using Abbott’s formula to adjust for natural mortality before probit analysis, ensuring the accuracy of the estimated toxicity parameters:

$$Corrected~Mortality~\left( \% \right)=\left[ {~\frac{{Observed~Mortality~\% - Control~Mortality~\% }}{{100 - Control~Mortality~\% }}} \right]*100$$

Accordingly, the LC_50_ values presented in the results represent statistically corrected data that account for natural death rates in the control group. The precision and validity of the LC_50_ estimates were assessed using multiple statistical measures. Chi-square (χ^2^) analysis was performed to assess the goodness-of-fit between the observed and expected mortality data, and the calculated values were compared against the critical threshold of 7.8 at a 0.05 significance level. Additional model validation employed the Finney method and determination of the coefficient of determination (r^2^) to confirm the reliability of the probit regression analysis.

The relative potency of the tested compounds was assessed by calculating the Toxicity Index (T.I.) using Sun’s equations^37–39^:

$${\mathrm{Toxicity~index}} = \frac{{{\mathrm{LC50~of~most~effective~compound}}}}{{{\mathrm{LC50~of~the~compound~used}}}}{\mathrm{~}} \times {\mathrm{100}}$$

### Molecular docking studies

#### Receptor preparations

The selection of molecular targets for docking simulations was based on the neurotoxic signs observed during larvicidal assays, which suggested interference with neural transmission. Consequently, *Culex pipiens* acetylcholinesterase (AChE) (UniProt accession number: Q86GC8) and nicotinic acetylcholine receptor (nAChR) (UniProt accession number: B0XBN4) were chosen. The UniProt Knowledgebase (https://www.uniprot.org/) served as the source for retrieving the amino acid sequences of both targets. Since experimentally resolved crystal structures for these specific mosquito receptors are absent from the Protein Data Bank (PDB), homology modeling was conducted to obtain reliable three-dimensional (3D) structural models.

The SWISS-MODEL server (https://swissmodel.expasy.org/) was employed to perform homology modeling, owing to its recognized accuracy in protein structure prediction^40–46^. The server integrates BLASTp and HHblits algorithms to identify the most suitable structural templates from the SWISS-MODEL Template Library (SMTL) and PDB. The best templates were selected based on sequence similarity and coverage and then used to construct the 3D models. Model reliability was verified through multiple quality assessment metrics, including Z-score analysis, QMEAN evaluation, and calculation of the General Model Quality Estimate (GMQE). All assessments confirmed that the generated structures were of high quality and structural accuracy^40,41,47^.

#### Molecular docking assessment

The chemical structures of all synthesized 3-methyl-pyrazole derivatives were first sketched in ChemDraw 20.0 and subsequently transformed into three-dimensional (3D) formats using the Molecular Operating Environment (MOE, version 2024.06; https://www.chemcomp.com/en/index.htm). Prior to docking, ligand preparation involved adjusting protonation states to match physiological pH, computing partial charges, and performing energy minimization to obtain the most stable conformations^30–32,48–51^.

The receptor structures were prepared in MOE by detecting the ligand-binding regions using the Site-Finder module. This tool identified alpha-site pockets on the protein surface that could serve as active sites, and dummy atoms were placed to define the docking coordinates for subsequent simulations. The docking procedure was executed using the London ΔG scoring function in MOE, which generated 100 initial binding poses for each ligand in the defined active site. These poses were ranked according to their docking scores, and the 20 top-scoring conformations were retained for detailed inspection.

Selection of the final binding pose for each compound was based on several parameters: the lowest calculated binding free energy (S-score), a root mean square deviation (RMSD) less than 2 Å from the initial docked orientation, and the presence of favorable molecular interactions within the binding site, including hydrogen bonding, hydrophobic contacts, π–π stacking, and other non-covalent forces. Docking simulations were performed with the receptor kept rigid to maintain structural stability, while ligands were allowed full conformational flexibility to explore different orientations and binding modes.

To ensure the credibility of the docking methodology, validation was performed using known insecticidal agents as reference ligands. For AChE, chlorpyrifos, a well-characterized organophosphate insecticide, was docked into the active site under identical conditions to the test compounds, allowing for direct comparison of docking scores and interaction patterns. For nAChR, modeled using AlphaFold-predicted structural data, validation involved docking three neonicotinoid insecticides, thiamethoxam, clothianidin, and imidacloprid using the same docking parameters. The interaction profiles and docking energies of these reference ligands were compared with those of the synthesized derivatives, providing a benchmark for evaluating binding affinities and supporting the biological relevance of the computational predictions.

### Molecular dynamics simulations

To extend the docking results and examine time-dependent stability and binding behavior, molecular dynamics (MD) simulations were executed for the highest-scoring complexes using the Desmond Maestro 2022.1 platform from Schrödinger, Inc. The simulations focused on three systems: derivative **“7”**–AChE, derivative **“12”**–AChE, and chlorpyrifos–AChE—each initiated from the top-ranked docking pose to ensure biologically meaningful starting conformations. Every complex was embedded in an orthorhombic water box filled with TIP3P molecules, maintaining a 10 Å buffer in all directions. System neutrality and physiological salt conditions were imposed by adding Na⁺/Cl⁻ counterions to reach an ionic strength of 0.15 M^49,52^.

The OPLS_2005 force field was applied for parameterization of the protein, ligand, and solvent components. Energy minimization was performed initially, following a steepest-descent approach, until the energy gradient was reduced to below 25 kcal·mol^–1^ Å^–1^. Subsequent equilibration was carried out under NPT conditions, maintaining a temperature of 300 K and a pressure of 1 atm. A Berendsen thermostat (coupling constant 1.0 ps) and barostat (coupling constant 2.0 ps) were used to control temperature and pressure, respectively. For the production phase, simulations were run for 100 ns with a 2-fs integration step. Coordinate data were recorded every 1.2 ps, ensuring adequate sampling for later analysis of transient molecular interactions^49,52^.

Trajectory evaluation included RMSD of the protein backbone to monitor overall stability, RMSF to quantify residue-level flexibility (with emphasis on binding-site loops and catalytic residues), and secondary-structure profiling to track any time-dependent reorganization of the receptor. Protein–ligand contacts—notably hydrogen bonds, hydrophobic contacts, ionic links, and water-mediated bridges—were quantified using Maestro’s Simulation Interactions Diagram utility to capture both frequency and persistence of key interactions across the 100-ns windows^49,52^.

## Results and discussion

### Chemistry

**Scheme 1:**
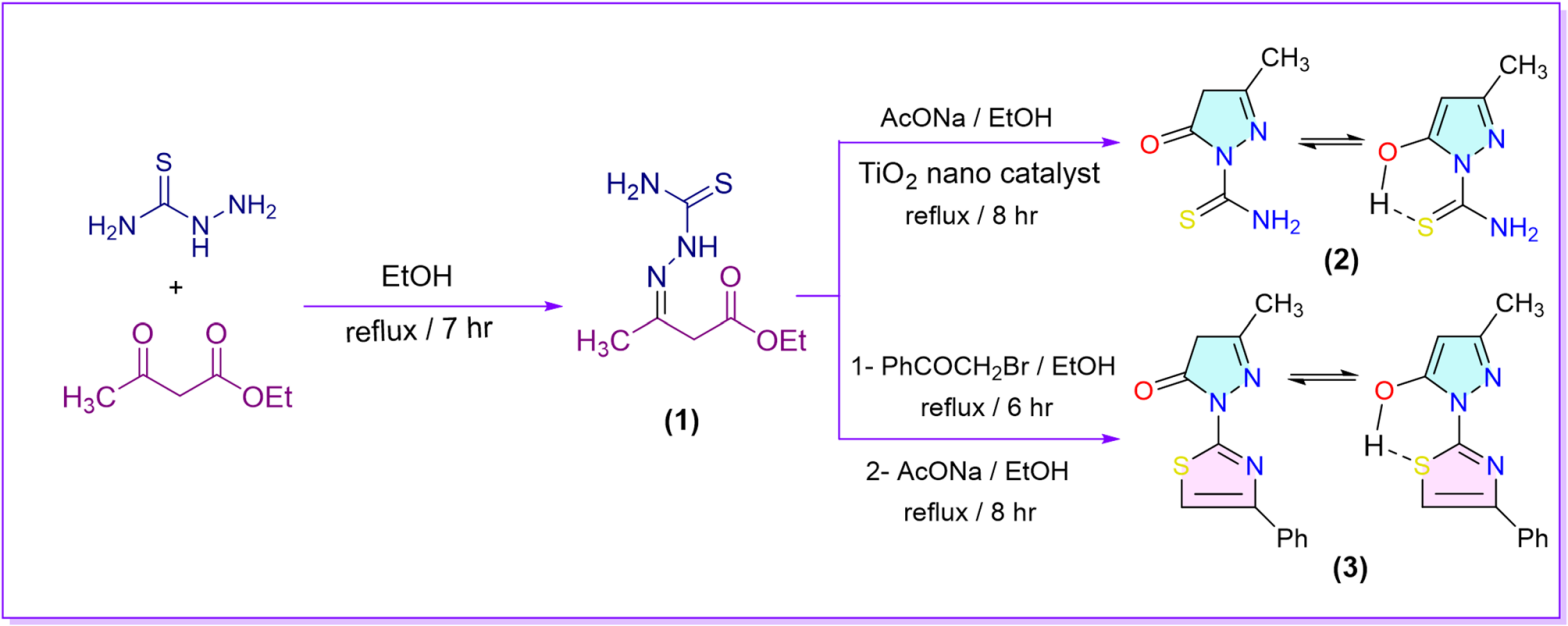
Synthesis of the key starting pyrazolone derivatives **2** and **3**.

In our methodology, we synthesized the 3-methylpyrazolone derivative **2** using two approaches. The first approach, based on the literature^53^, which involved condensing ethyl acetoacetate with thiosemicarbazide in the presence of a few drops of acetic acid under reflux conditions, Ethyl-3-(2-carbamothioylhydrazineylidene) butanoate **(1)**^54^ was smoothly obtained as the sole product. Afterwards, this intermediate was refluxed with fused sodium acetate in ethanolic solution to yield 3-methyl-5-oxo-4,5-dihydro-1*H*-pyrazole-1-carbothioamide **2** with a 40% yield as a key starting material. In the second approach, the reaction yield improved to 85% via the incorporation of TiO_2_ nanoparticles as a catalyst to the reaction mixture under the same conditions. The ^1^H-NMR spectrum of compound 2 exhibited a broad singlet signal at 17.32 ppm corresponding to OH proton, which is more deshielded due to forming the intramolecular 6-membered chelation hydrogen bond. Also, it displayed a singlet signal at 7.36 ppm corresponding to C_4_-H of pyrazole moiety. On the other hand, treating compound **1** with phenacyl bromide under reflux conditions yielded ethyl 3-(2-(4-phenylthiazol-2-yl)hydrazineylidene)butanoate^55^. After that, the latter was refluxed in ethanol with fused sodium acetate to give 5-methyl-2-(4-phenylthiazol-2-yl)-2,4-dihydro-3*H*-pyrazol-3-one (**3**)^55^ (Scheme 1).

**Scheme 2:**
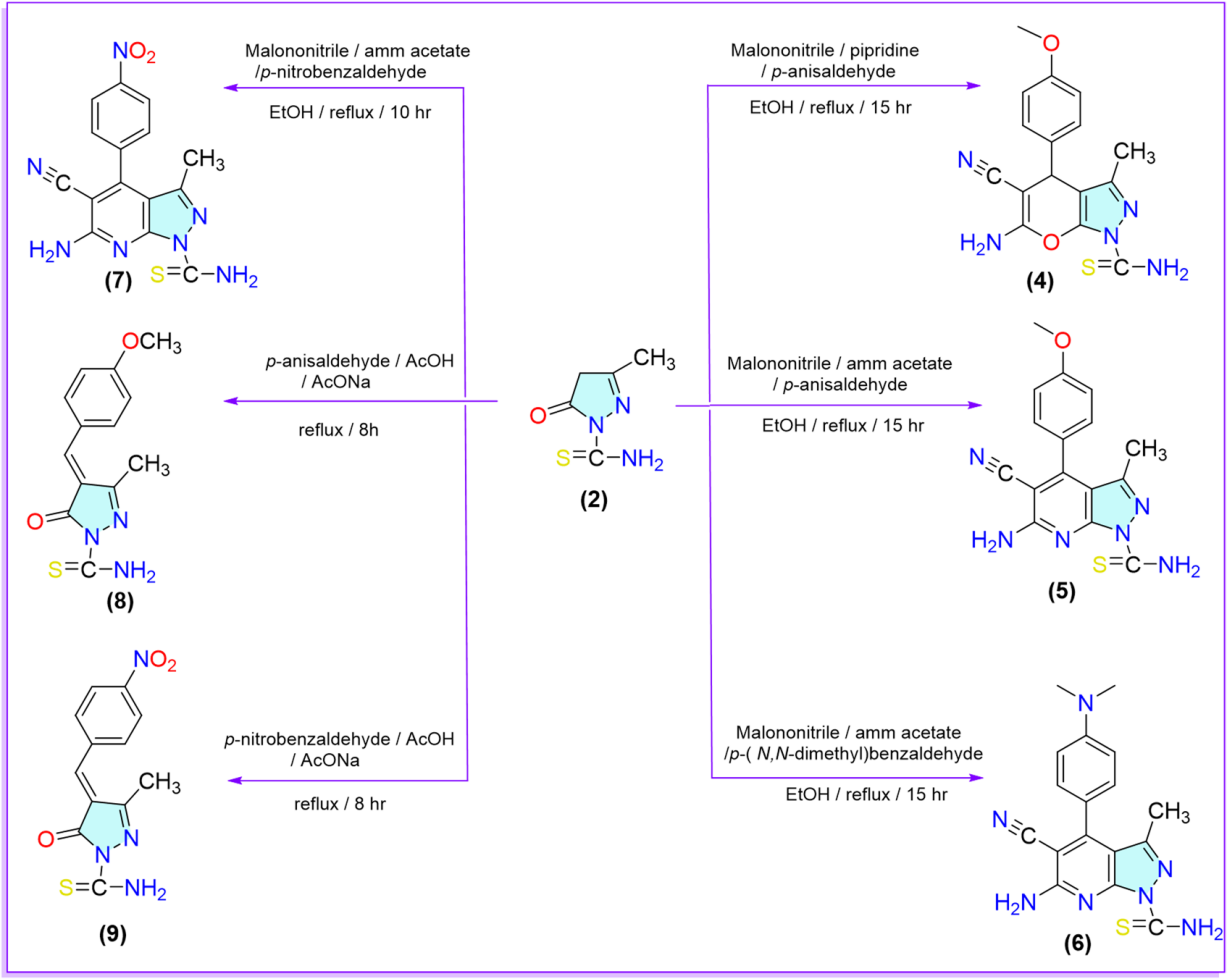
Reaction of pyrazolone derivative **2** with different carbon electrophiles.

The nucleophilicity of CH_2_ of the pyrazolone ring was tested by the reaction with different carbon electrophiles. The one-pot multicomponent reaction of pyrazolone **1** with different aromatic aldehydes and malononitrile in the presence of piperidine led to the formation of pyranopyrazole derivative **4**. On the other hand, the using of ammonium acetate instead of piperidine under the same reaction conditions led to the formation of pyrazolopyridine derivatives **5–7**. Spectral analyses confirmed the structures of the target compounds **4–7**. For instance, the IR spectrum of compound **4** showed absorption bands at 2202 cm^–1^ for the C ≡ N group and 3333 and 3216 cm^–1^ for NH_2_. Additionally, the ^1^H-NMR spectrum exhibited two broad singlet signals at 6.88 and 6.87 ppm exchangeable with D_2_O, compatible with the corresponding two NH_2_ protons, and a singlet signal at 3.83 ppm corresponding to the C_4_-H of pyranopyrazole moiety. Also, the IR spectra for compounds **5–7** displayed absorption bands at 2202, 2203, and 2209 cm^–1^ for the conjugated C ≡ N group and at (3476 and 3363), (3346 and 3204), and (3458 and 3347) cm^–1^ for the NH_2_ group, respectively. Additionally, the ¹H‐NMR spectrum revealed singlet signals at (7.09 and 7.07), (8.57 and 7.07) and (11.34 and 4.93) ppm, corresponding to the two NH_2_ protons. Furthermore, Compounds **8** and **9** formed by the activation of the CH_2_ of pyrazolone ring **2** by fused sodium acetate and followed by nucleophilic attack on the carbonyl group of the aromatic aldehydes, resulted in the formation of arylidine derivatives **8**, and **9**. Spectral analyses confirmed the structures of compounds **8** and **9**. For instance, the ^1^H‐NMR spectra showed singlet signals at (6.99 and 2.12) and (8.69 and 2.27) ppm corresponding to the CH olefinic protons and CH_3_ protons. respectively, and two doublet signals at (7.04 and 7.02) and (8.58 and 8.34) ppm corresponding to the four protons of aromatic ring, respectively, besides the appearance of a singlet signal at 3.62 ppm corresponding to OCH_3_ protons of compound **8** (Scheme 2).

**Scheme 3:**
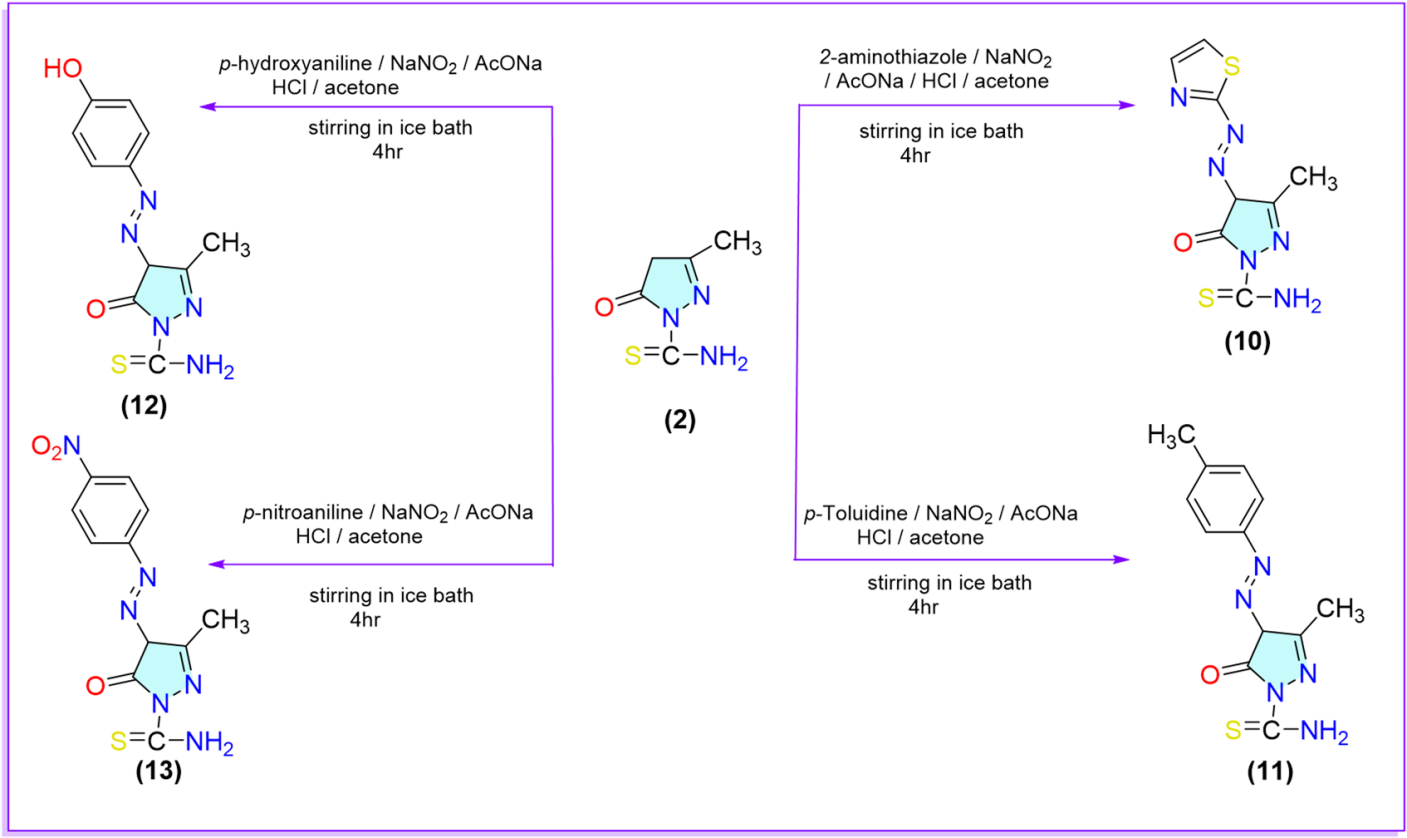
Reaction of pyrazolone derivative **2** with different nitrogen electrophiles.

To further investigate the nucleophilic character of the methylene group of pyrazolone derivative **2**, compound **2** was subjected to diazo coupling reactions with a variety of aryl diazonium chlorides. Specifically, compound **2** was stirred with thiazole diazonium chloride, *p*-toluene diazonium chloride, *p*-nitroaniline diazonium chloride, and *p*-hydroxyaniline diazonium chloride in acetone containing sodium hydroxide at 0 °C. These reactions yielded the corresponding azo derivatives **10–13**. Compounds **11–13** have been previously reported in the literature^56,57^. The primary objective of synthesizing these derivatives was to evaluate their insecticidal activity, particularly in the context of substituent effects. Both the electron-donating groups (e.g., OH, CH₃) and the electron-withdrawing groups (e.g., NO₂), as well as the incorporation of heterocyclic moieties such as thiazole, were considered for their potential influence on biological activity. Spectral analysis verified the structure of compounds **10**–**13**. For example, the ^1^H-NMR spectrum of compound **10** showed two broad signals at singlets 7.58 and 7.21 ppm corresponding to thiazole moiety protons. On the other hand, the ^1^H‐NMR spectrum of compound **11** showed singlet signals at 2.31 ppm corresponding to the CH_3_ protons (Scheme 3).

4-Amino-5-imino-2-methyl-5,6-dihydro-7*H*-3-thia-1,6,7a-triazacyclopenta[*c*,*d*]indene-7-thione **(14)** was efficiently synthesized under Gewald reaction conditions in a one-pot multicomponent approach. This involved stirring an ethanolic solution of compound **2** with malononitrile and elemental sulfur in the presence of a catalytic amount of morpholine as a base at 60 °C. Incredibly, we obtained the tricyclic compound **14** instead of bicyclic compound **14**^**\**^. The reaction takes place through the Gewald reaction mechanism, which afforded cyano[3,2-c]pyrazole derivative **14**^**\**^ as an intermediate, followed by cyclization through 1,6-exo-dig cyclization via the attack of the lone pair of the NH_2_ group on the carbon of the cyano group (CN). The spectral data supported the chemical structure of compound **14**. Whereas the IR spectrum showed the disappearance of the CN group, beside the appearance of absorption bands at 3269, 3201, and 3141 cm^−1^, corresponding to NH and NH_2_ groups. Additionally, the ^1^H-NMR spectrum displayed three singlet signals at 11.34, 8.73, and 6.57 ppm consistent with two NH and NH_2_ protons, respectively.

**Scheme 4:**
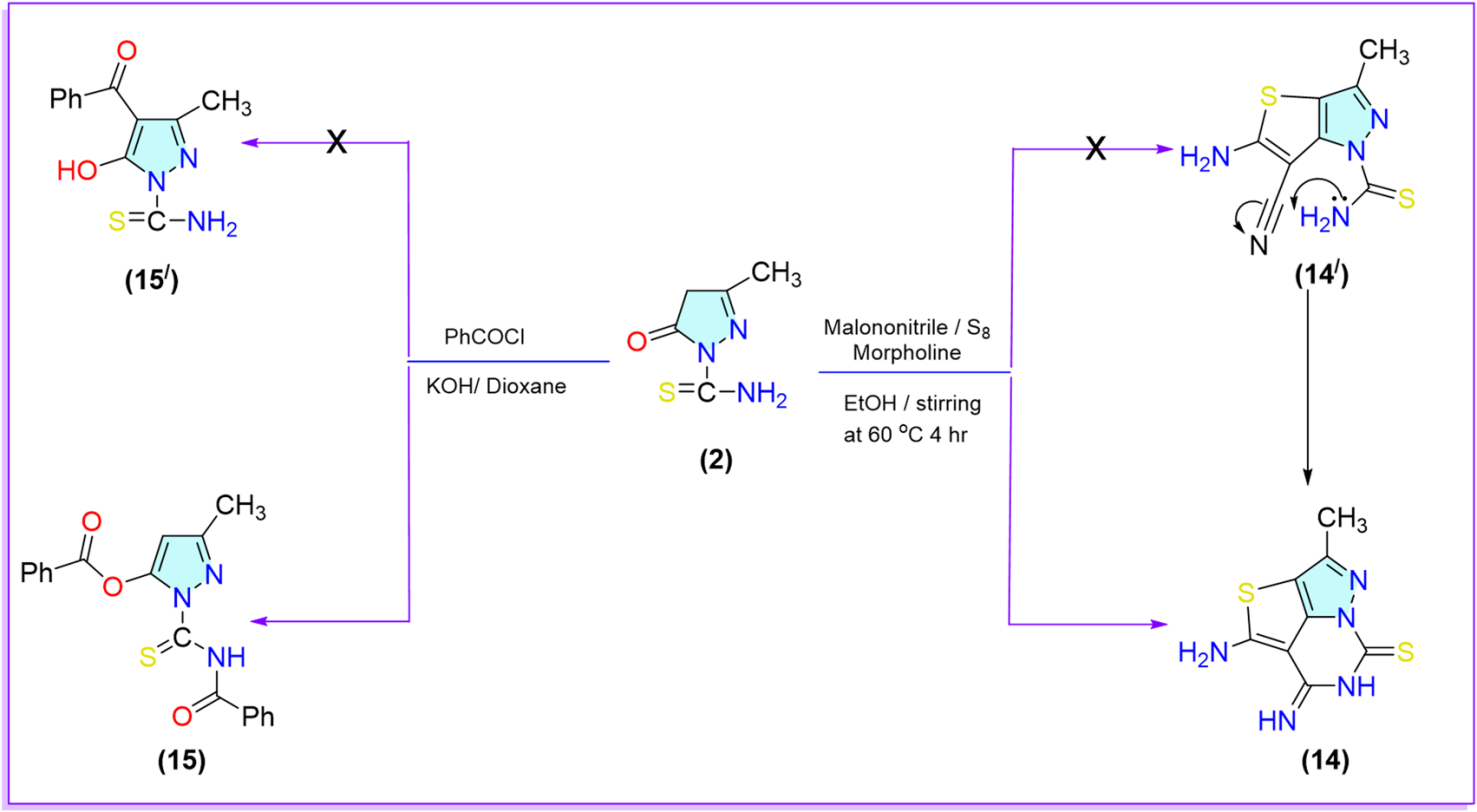
Synthesis of pyrazole derivatives **14**, **15**.

On the other hand Reaction of compound **2** with benzoyl chloride in the presence of potassium hydroxide as a base under reflux conitions for 10 h afford smoothly 1-(benzoylcarbamothioyl)-3-methyl-1*H*-pyrazol-5-yl benzoate (**15)** rather than formation of 4-benzoyl-5-hydroxy-3-methyl-1H-pyrazole-1-carbothioamide **(15**^**\**^**)** that refer to dibenzoylation occur on (OH) and (NH_2_) groups instead of benzoylation on the active methylene. The chemical structure of compound **15** was supported by the spectral data. Whereas the IR spectrum showed two bands at 1748, 1707 cm^− 1^ corresponding to two C = O of ester and amide, and the appearance of (NH) group at 3416 cm^− 1^ instead of (NH_2_) group. The ^1^H-NMR showed signals at 7.68 ppm corresponding to C_4_-H of the pyrazole ring and multiplet signals at 8.09–7.51 ppm corresponding to ten aromatic protons of the two phenyl groups (Scheme 4).

**Scheme 5:**
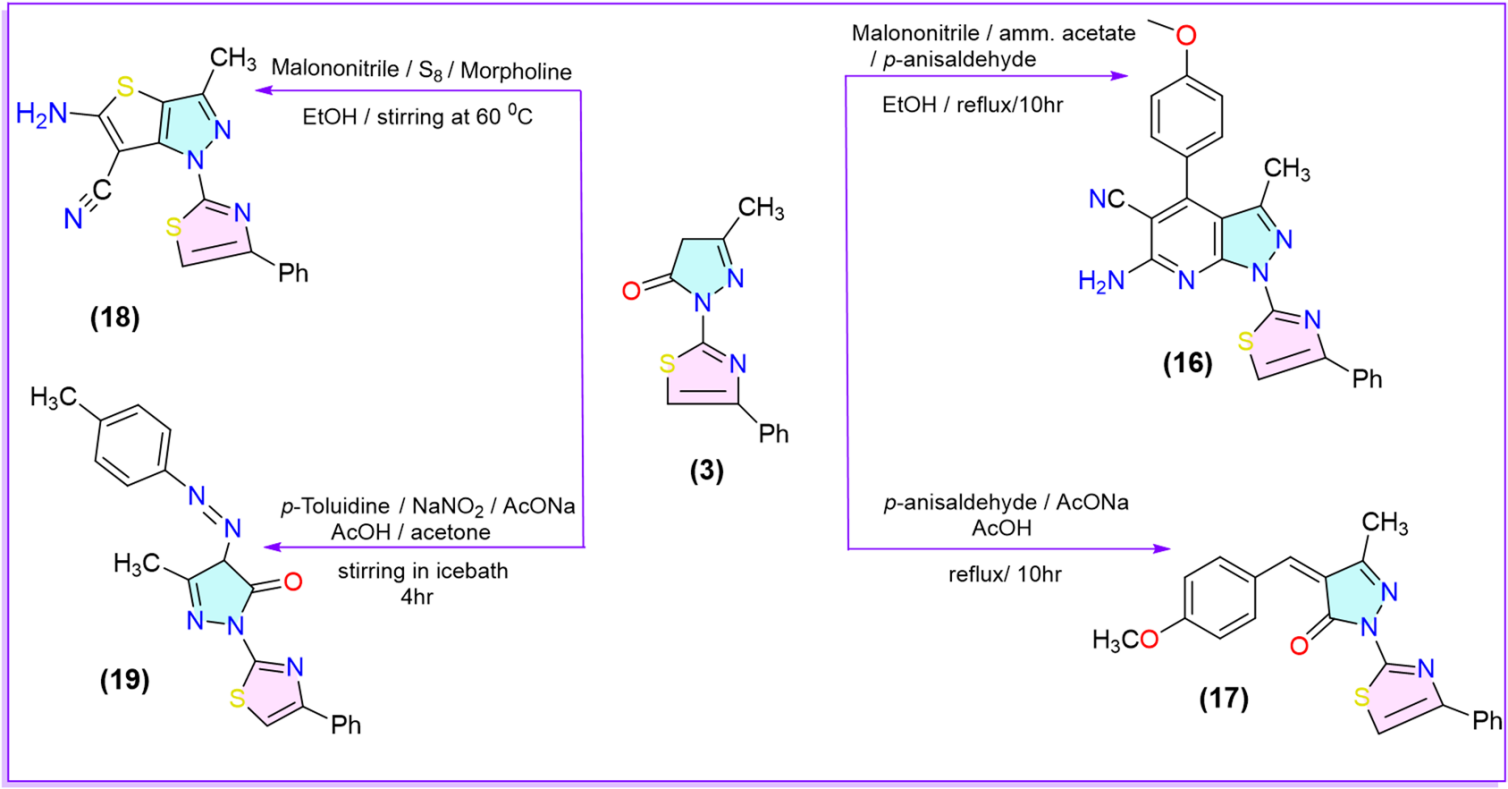
Reaction of pyrazolone derivative **3** with different electrophilic centers.

To enable a comparative assessment of the insecticidal potency between thioamide pyrazolone derivative **2** and thiazolyl pyrazolone derivative **3**. So, the same kind of reactions performed on compound **2** were repeated using compound **3** as the starting material. The reaction of compound **3** with aldehydes, particularly *p*-anisaldehyde, formed the arylidene intermediate. This intermediate subsequently cyclized with malononitrile in the presence of ammonium acetate, producing the compound 6-amino-4-(4-methoxyphenyl)-3-methyl-1-(4-phenyl thiazol-2-yl)-1*H*-pyrazolo[3,4-*b*]pyridine-5-carbonitrile **(16)**. Spectral analysis confirmed the structure of compound **16**. The IR spectrum showed absorption bands at 2206 cm⁻¹ for the C ≡ N group and 3366 (br.) cm^− 1^ for the NH_2_ group. Furthermore, the ^1^H-NMR spectrum displayed two singlet peaks at 7.07 ppm (exchangeable with D_2_O) and 3.86 ppm corresponding to NH_2_ protons and OCH_3_ protons. Additionally, the condensation of compound **3** with *p-*anisaldehyde, in acetic acid in the presence of fused sodium acetate, yielded 4-(4-methoxybenzylidene)-5-methyl-2-(4-phenylthiazol-2-yl)-2,4-dihydro-3*H*-pyrazol-3-one **(17)**. The spectral analysis verified the structure of the compound **17**, the ^1^H-NMR spectrum showed a singlet signals at 8.71, and 3.92 ppm corresponding to the olefinic CH, and OCH_3_ protons. Furthermore, Thieno[3,2-*c*]pyrazole derivative **(18)** was smoothly synthesized using Gewald reaction conditions through a one-pot, multicomponent approach. The IR spectrum showed absorption bands at 2201 and 3327 (br.) cm^− 1^ corresponding to C ≡ N and NH_2_ groups. Furthermore, the ^1^H-NMR spectrum displayed a singlet at 3.75 ppm, which matched the NH_2_ protons. Finally, when compound **3** stirred with *p*-toluene diazonium chloride, in acetone with sodium hydroxide at 0 °C, the compound 5-methyl-2-(4-phenylthiazol-2-yl)-4-(*p*-tolyldiazenyl)-2,4-dihydro-3*H*-pyrazol-3-one **(19)** was obtained as literature^39^ (Scheme 5).

### Insecticidal bioassay

The insecticidal potential of the synthesized compounds was evaluated by *exposing Culex pipiens* third-instar larvae to 19 different 3-methyl-pyrazole derivatives with varied structural features. The larvicidal bioassay revealed a wide range of toxic responses, with LC_50_ values varying significantly among the tested compounds (Table 1). When sorted in ascending order, derivative “**7**” (LC_50_ = 0.16 µg/mL) exhibited the strongest insecticidal activity, clearly outperforming the reference insecticide chlorpyrifos (LC_50_ = 52.90 µg/mL). The LC_50_ value obtained for chlorpyrifos falls within the range reported for laboratory-maintained or tolerant *Culex pipiens* colonies, which commonly display variable sensitivity to organophosphates depending on strain history and test conditions^58–65^. Thus, in this work, chlorpyrifos is used primarily as an internal benchmark under consistent experimental settings rather than as a universal toxicity reference.

Closely following in potency, derivative “**12**” (LC_50_ = 0.30 µg/mL) also demonstrated high larvicidal activity. Both compounds were significantly more effective than the rest of the tested derivatives and are thus classified as highly potent candidates.

The next group of compounds showed considerably higher LC_50_ values and consequently lower toxicity. These included Derivative “**4**” (LC_50_ = 9.70 µg/mL), derivative “**11**” (LC_50_ = 9.90 µg/mL), and derivative “**9**” (LC_50_ = 10.10 µg/mL), which can be regarded as moderately potent. Other compounds such as derivative “**14**” (LC_50_ = 30 µg/mL), derivative “**19**” (LC_50_ = 30.90 µg/mL). Control mortality across all assays remained consistently below 5%, confirming the reliability and validity of the recorded LC₅₀ values.

Across all treatments, the larvae exhibited consistent neurotoxic symptoms including hyperactivity, erratic movement, tremors, and eventual paralysis. These signs closely mirrored those observed in chlorpyrifos-exposed larvae, suggesting a similar or overlapping mode of action, likely involving disruption of neural transmission through acetylcholinesterase inhibition or interaction with nicotinic acetylcholine receptors.

To facilitate a standardized comparison of insecticidal potency, the Toxicity Index (TI) was calculated for each compound using derivative “**7**” as the reference standard (TI = 100). Among all compounds, only derivative “**12**” approached this level of activity, with a TI of approximately 53.3. As such, both compounds are identified as highly potent larvicides. All other derivatives displayed significantly lower TI values, confirming their moderate to low activity relative to derivative “7”. For instance, derivative “4” had a TI of 1.6, derivative “11” a TI of 1.5, and derivative “2” a TI of 0.7.

All LC_50_ values presented here were derived from probit analysis incorporating Abbott’s correction for natural control mortality, ensuring that the reported values accurately reflect true toxic effects. All χ^2^ values associated with the probit regression models remained below the critical threshold of 7.8, confirming the statistical validity of the dose–response data. These results support the reliability of the LC_50_ and TI estimations and highlight derivative “**7**” and derivative “**12**” as promising candidates for further development as larvicidal agents targeting *Culex pipiens*. Furthermore, the recalculated χ^2^ p-values for all tested compounds exceeded 0.05, providing additional statistical assurance of model fit and validating the consistency of the regression analyses.

**Table 1:** The insecticidal efficacy of 19 synthesized compounds was evaluated against the 3rd instar larvae of *Culex pipiens* over a 24-hour period, with chlorpyrifos used as a reference insecticide, and results were reported at a 95% confidence interval (C.I).

Compound	LC_50_/ µg/mL	χ^2^cal./tab. _(7.8)_	*r* ^2^	χ^2^ *P*-value	Toxicity index
**1**	15.50	3	0.98	0.89	1.01
**2**	22.70	7.2	0.93	0.41	0.7
**3**	15.60	1.1	0.99	0.99	1.02
**4**	9.70	7.4	0.94	0.39	1.6
**5**	19.80	7.8	0.96	0.35	0.8
**6**	11.20	7.3	0.91	0.39	1.4
**7**	0.16	0.2	0.97	0.99	100
**8**	23.00	7.6	0.96	0.41	0.69
**9**	10.10	6.8	0.96	0.45	1.5
**10**	26.90	7.8	0.94	0.35	0.59
**11**	9.90	7.7	0.95	0.36	1.61
**12**	0.30	0.5	0.95	0.99	53.3
**13**	29.20	6.6	0.96	0.47	0.54
**14**	30.00	7.6	0.94	0.37	0.53
**15**	14.40	0.8	0.99	0.99	1.1
**16**	28.60	7.6	0.92	0.37	0.55
**17**	23.10	7.5	0.96	0.38	0.69
**18**	28.60	7.3	0.94	0.59	0.559
**19**	30.90	5.5	0.98	0.60	0.51
**Chlorpyrifos**	52.90	1.8	0.95	0.97	0.3

### Molecular docking assessment and SAR relationship

To explore the possible mode of action, molecular docking was performed on nineteen synthesized 3-methyl-pyrazole derivatives targeting two principal neural proteins in *Culex pipiens*. The first target, acetylcholinesterase (AChE), is an enzyme that terminates nerve impulses by catalyzing the breakdown of acetylcholine in the synaptic cleft. The second, the nicotinic acetylcholine receptor (nAChR), is a ligand-gated ion channel essential for rapid synaptic transmission. These targets were chosen based on neurotoxic symptoms observed during larvicidal assays, which pointed to disruption of cholinergic signaling. Their selection was further supported by the structural similarity of the synthesized derivatives to known insecticides organophosphates, which inhibit AChE, and neonicotinoids, which act as nAChR agonists. Inhibition of AChE, as seen with chlorpyrifos, leads to excessive accumulation of acetylcholine, prolonged depolarization of the postsynaptic membrane, continuous nerve firing, paralysis, and ultimately death^47,66^. In contrast, neonicotinoids—including thiamethoxam, imidacloprid, and clothianidin bind to nAChRs as acetylcholine mimics, persistently opening ligand-gated ion channels. This prolonged excitation disrupts neuronal homeostasis, leading to hyperactivity, paralysis, and mortality^67–69^.

The structural design of the synthesized 3-methyl-pyrazole derivatives was deliberately informed by the incorporation of functional groups with well-established insecticidal relevance. The series incorporates diverse pharmacophores—such as sulfur atoms, amino (–NH₂), cyano (–CN), nitro (–NO₂), and carbonyl (C = O) functionalities—integrated with aromatic and heteroaromatic scaffolds, including the pyrazole core. Such motifs are characteristic of classical acetylcholinesterase (AChE) inhibitors and nicotinic acetylcholine receptor (nAChR) agonists; for example, chlorpyrifos contains a P = S moiety essential for AChE binding, while neonicotinoids frequently employ nitro or cyano substituents to enhance nAChR affinity. This chemical diversity reflects a rational design strategy aimed at maximizing insecticidal potency through simultaneous engagement of multiple neural targets. In addition to strengthening target–ligand interactions via diverse binding environments, these structural elements are expected to enhance physicochemical properties critical to in-vivo performance, such as solubility, lipophilicity, metabolic stability, and penetration through the insect cuticle, thereby reinforcing the premise that AChE and nAChR serve as primary mediators of the observed biological activity.

Due to the lack of resolved crystal structures for *Culex pipiens* AChE and nAChR in the Protein Data Bank (PDB), three-dimensional models of these targets were generated through homology modeling. The AChE model was constructed using the *Anopheles gambiae* AChE structure complexed with phenylmethylsulfonyl fluoride (SMTL ID: 5ydj.1), while the nAChR model was based on the *Aedes aegypti* Noppera-bo receptor. Structural validation yielded GMQE scores of 0.84 for AChE and 0.91 for nAChR, reflecting high confidence in model accuracy and active-site integrity for docking analyses.

Docking simulations (Table 2) revealed that all nineteen derivatives exhibited binding affinities comparable to, or exceeding, those of the reference insecticides. Derivative “**7**”, the most potent in bioassays (LC_50_ = 0.16 µg/mL), showed strong interactions with both AChE (–6.70 kcal/mol) and nAChR (–5.78 kcal/mol). For comparison, chlorpyrifos docked to AChE with a score of − 6.61 kcal/mol, while thiamethoxam, clothianidin, and imidacloprid bound to nAChR with scores of − 7.11, − 6.43, and − 6.62 kcal/mol, respectively.

**Fig. 1:**
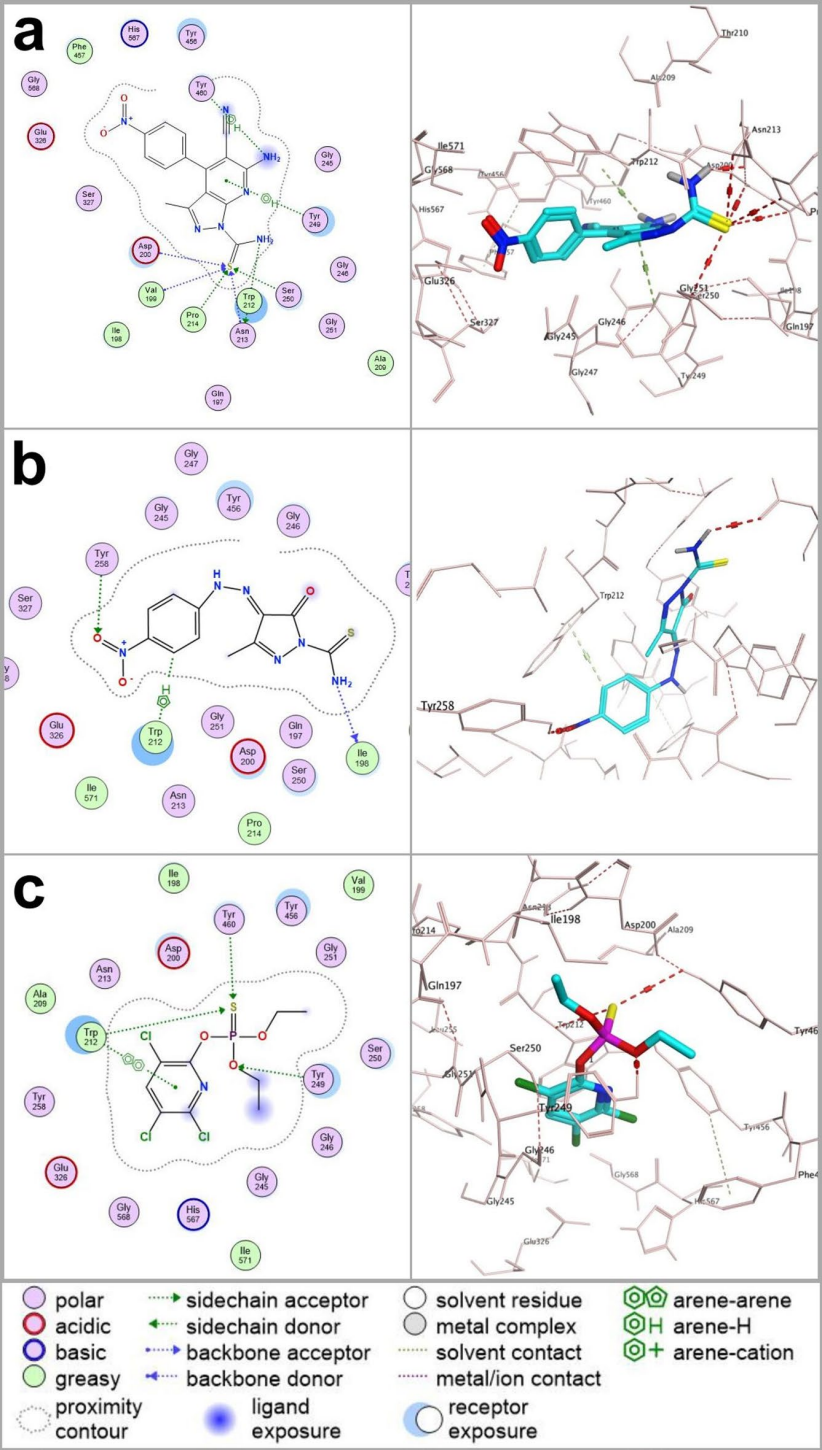
Two-dimensional (2D) and three-dimensional (3D) molecular docking interactions illustrating the binding mode of (a) derivative **7**, (b) derivative **12**, and (c) chlorpyrifos with the acetylcholinesterase (AChE) enzyme from *Culex pipiens*.

**Fig. 2:**
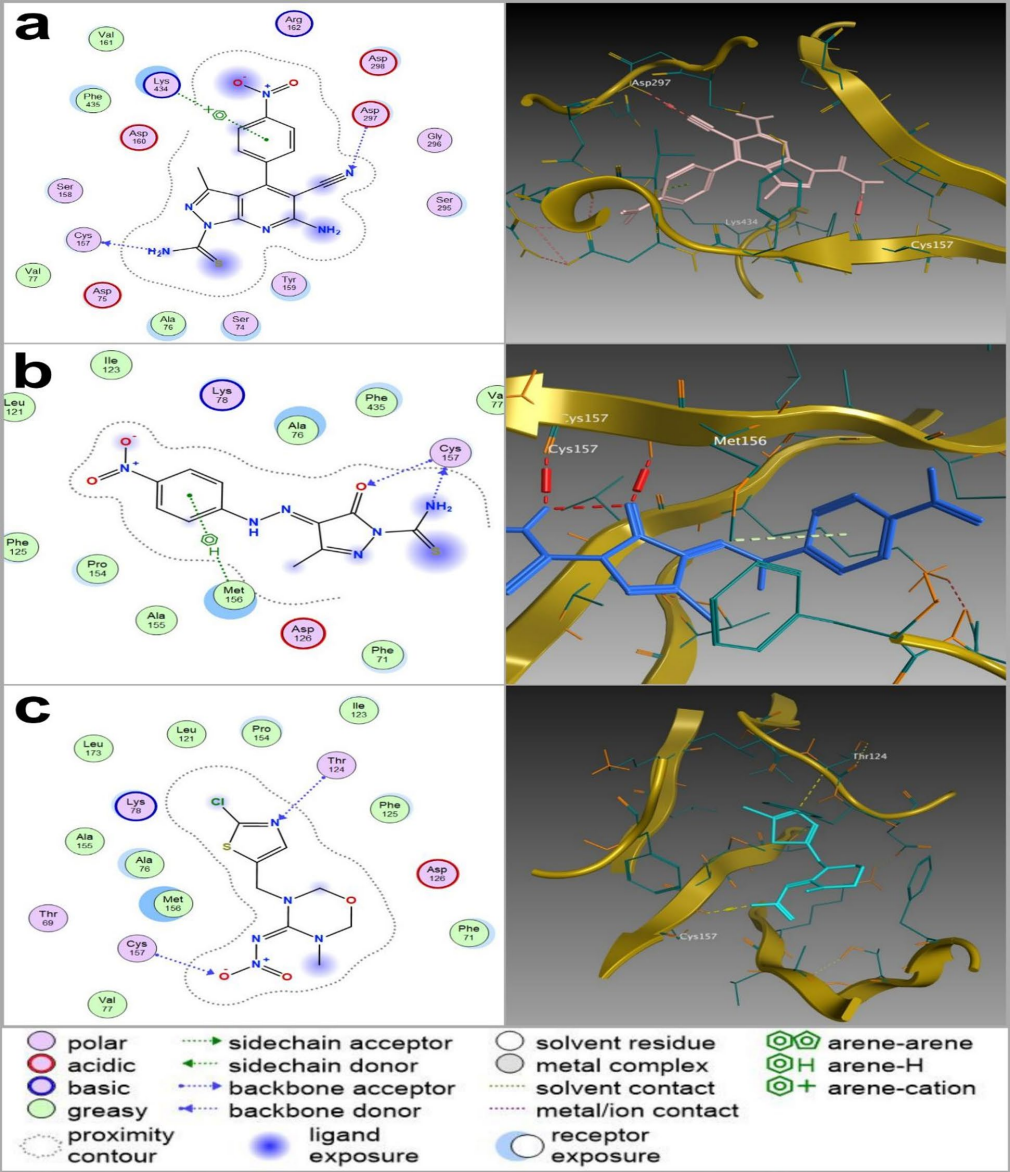
Two-dimensional (2D) and three-dimensional (3D) molecular docking interactions depicting the binding modes of (a) derivative **“7”**, (b) derivative **“12”** and (c) the reference insecticide thiamethoxam with the nicotinic acetylcholine receptor (nAChR) from *Culex pipiens*. Derivatives **“7”** and **“12”** exhibit strong binding interactions, including hydrogen bonding and π-cation interactions, with critical receptor residues, indicative of potent neurotoxic potential. Thiamethoxam similarly engages key residues through distinct hydrogen bond interactions, serving as a benchmark for comparative evaluation.

All docked compounds adopted stable binding conformations, as indicated by root mean square deviation (RMSD) values below 2 Å—ranging from 0.58 to 1.76 Å for AChE and 0.76 to 1.87 Å for nAChR. Binding stability was further corroborated through pose visualizations and detailed interaction mapping (Table 3; Figs. 1 and 2). For clarity, the main text presents representative docking results for the two most biologically active derivatives (**7** and **12**), which were selected based on their superior larvicidal performance. These compounds displayed typical hydrogen-bonding, hydrophobic, and π–π stacking interactions within the catalytic sites of AChE and nAChR, consistent with the interaction profiles observed across the series. Comparative analysis revealed that derivatives **7** and **12** engaged their targets through interaction patterns closely resembling those of known insecticides, including chlorpyrifos (AChE inhibitor) and neonicotinoids such as thiamethoxam, clothianidin, and imidacloprid (nAChR agonists). This similarity in binding behavior suggests that the newly synthesized molecules may act through a comparable neurotoxic mechanism, primarily involving interference with cholinergic signaling. Among the two, derivative **7** formed a slightly greater number of hydrogen-bond and π–π stacking interactions with AChE than chlorpyrifos, which may partially explain its higher observed larvicidal potency. In contrast, derivative **12** exhibited an interaction profile and bond count more comparable to chlorpyrifos, reinforcing the view that both compounds follow a similar binding mode despite differences in biological activity. Complete docking data and interaction maps for all nineteen synthesized derivatives are available in the Supporting Information (Tables S1-S4), following the same analytical format used for derivatives **7** and **12**.

Structure–activity relationship (SAR) evaluation highlighted the critical role of certain functional groups in enhancing receptor binding strength. Sulfur-containing moieties, whether embedded in heterocyclic frameworks or as substituents, facilitated hydrogen bonding and polar interactions within catalytic sites. Electron-withdrawing groups such as cyano and carbonyl increased electrophilicity, thereby enhancing target engagement, while amino groups functioned as hydrogen-bond donors, contributing to complex stabilization. These functional features were prominent in the most active derivatives and frequently yielded more extensive or diverse interactions than those observed for reference insecticides, suggesting distinct and potentially superior binding characteristics for the newly synthesized molecules.

The docking outcomes strongly supported the initial target selection rationale, as the synthesized 3-methyl-pyrazole derivatives, particularly compounds **7** and **12**, displayed interaction motifs, binding residues, and orientations closely resembling those of reference insecticides acting on AChE and nAChR. This convergence between predicted binding behavior and the established neurotoxic profiles of organophosphates and neonicotinoids provides compelling evidence that the observed larvicidal effects likely arise from cholinergic pathway disruption. Further confirmation of binding stability and mechanistic reliability was provided by the subsequent molecular-dynamics (MD) analyses, which reinforced the docking observations and helped rationalize the potency differences among the most active derivatives.

The close correlation between molecular docking predictions and larvicidal bioassay data points to AChE and nAChR as the main neurotoxic targets of the tested compounds. However, docking affinity values alone do not completely account for the observed insecticidal effects. Discrepancies in LC₅₀ among derivatives with similar docking scores may be attributed to factors such as metabolic degradation rates, ability to penetrate the cuticle, or patterns of distribution within the insect body. Taken together, the deliberate structural design, strong computational binding profiles, and potent biological activity suggest that these receptors are the key mediators of toxicity for the synthesized 3-methyl-pyrazole derivatives, with derivative “**7**” and “**12**” standing out as the most potent example of dual-target engagement.

**Table 2:** Docking results for 19 synthesized compounds against *Culex pipiens* acetylcholinesterase (AChE) and nicotinic acetylcholine receptor (nAChR), showing binding affinities (S, kcal/mol) and root mean square deviations (RMSD, Å).

Compound	AChE		nAChR	
	*S	RMSD refine	S	RMSD refine
1	-5.25	0.68	-5.81	1.03
2	-4.90	1.67	-5.21	0.87
3	-6.21	1.03	-5.30	1.82
4	-6.13	1.05	-5.72	1.83
5	-6.72	1.27	-5.96	1.61
**6**	-6.74	1.64	-5.91	1.07
7	-6.70	1.49	-5.78	1.42
8	-6.58	0.73	-5.24	1.83
9	-6.63	1.22	-5.48	0.96
10	-6.13	1.71	-5.21	1.40
11	-6.52	0.90	-6.34	1.83
12	-6.98	1.58	-6.50	1.20
13	-6.56	1.65	-6.09	1.17
14	-5.40	0.58	-5.04	0.76
15	-6.19	1.74	-5.89	1.16
16	-7.86	0.80	-7.13	1.10
17	-7.78	0.81	-6.17	1.66
18	-6.94	1.01	-6.04	1.74
19	-7.52	1.35	-7.39	1.61
**Chlorpyrifos**	-6.61	0.81	-	-
**Thiamethoxam**	-	-	-7.11	1.38
**Clothianidin**	-	-	-6.43	1.22
**Imidacloprid**	-	-	-6.62	1.76

Reference insecticides include chlorpyrifos (AChE inhibitor) and the neonicotinoids thiamethoxam, clothianidin, and imidacloprid (nAChR agonists).

**Table 3:** Interaction profile of the most biologically active compounds, derivatives “7” and “12” with *Culex pipiens* neural targets in comparison with conventional insecticides.

Ligand-receptor complex	Residue	Interaction	Bond distance in A^0^	E (kcal/mol)
**7-AChE**	ASN 213 (B)	H-donor	2.71	-1.2
	VAL 199 (B)	H-acceptor	4.2	-0.9
	ASP 200 (B)	H-acceptor	4.1	-1.7
	ASN 213 (B)	H-acceptor	3.81	-0.8
	PRO 214 (B)	H-acceptor	3.57	-0.6
	SER 250 (B)	H-acceptor	3.8	-0.7
	TYR 460 (B)	H-pi	4.54	-0.6
	TYR 249 (B)	pi-H	3.9	-0.7
**12-AChE**	ILE 198 (B)	H-donor	3.34	-1.2
	TYR 258 (B)	H-acceptor	3.15	-1
	TRP 212 (B)	H-pi	3.61	-0.6
**Chlorpyrifos-AChE**	TRP 212 (B)	H-acceptor	3.52	-0.8
	TYR 460 (B)	H-acceptor	4.27	-0.8
	TYR 249 (B)	H-acceptor	3.02	-0.6
	TRP 212 (B)	pi-pi	3.97	-
**7-nAChR**	CYS 157 (A)	H-donor	3.17	-3.3
	ASP 297 (A)	H-acceptor	3.4	-1.3
	LYS 434 (A)	pi-cation	4.6	-0.9
**12-nAChR**	CYS 157 (A)	H-donor	3.13	-3.2
	CYS 157 (A)	H-acceptor	2.99	-2.8
	MET 156 (A)	pi-H	3.99	-0.5
**Thiamethoxam-nAChR**	CYS 157 (A)	H-acceptor	3.35	-0.8
	THR 124 (A)	H-acceptor	3.61	-0.5
**Clothianidin-nAChR**	CYS 157 (A)	H-acceptor	3.46	-0.5
**Imidacloprid-nAChR**	CYS 157 (A)	H-acceptor	2.88	-2.8

The table lists the interacting residues, interaction types, bond distances, and binding energies (E, kcal/mol) for each receptor–ligand complex. Data include interactions of derivatives “7” and “12” with acetylcholinesterase (AChE) and the nicotinic acetylcholine receptor (nAChR), alongside reference insecticides chlorpyrifos (AChE inhibitor) and three neonicotinoids: thiamethoxam, clothianidin, and imidacloprid (nAChR agonists).

All three ligands exhibit comparable hydrogen-bonding and hydrophobic interactions with residues located within the catalytic pocket, reflecting a similar binding orientation and interaction pattern. Derivative **7** formed a slightly higher number of stabilizing contacts, which may contribute to its higher experimental activity, whereas derivative **12** and chlorpyrifos displayed a broadly similar interaction profile. Overall, the docking results indicate that the new derivatives interact with AChE in a manner consistent with known organophosphate insecticides, supporting a shared mode of target engagement.

### Molecular dynamics simulation

To further verify and expand upon the docking findings and to validate the mechanistic rationale linking larvicidal activity to cholinergic pathway interference, molecular dynamics (MD) simulations were performed, offering a time-dependent and biologically relevant evaluation of ligand–protein interactions. In contrast to docking, which provides only a single static representation of possible binding modes, MD simulations incorporate receptor flexibility, the influence of solvent, and the continuous motion of molecular systems. This allows assessment of the overall complex stability, longevity of critical interactions, and the structural adaptability of both ligand and target protein over the simulation run^49–52,70,71^.

Derivatives **“7”** and **“12”** were identified as the most active larvicides in bioassays and were chosen for MD analysis due to their stronger docking affinity and more favorable interaction pattern with acetylcholinesterase (AChE) than with the nicotinic acetylcholine receptor (nAChR). Accordingly, AChE was selected as the main focus for dynamic evaluation. The simulation aimed to verify the stability of the bound conformation of derivatives **“7”** and **“12”** in the AChE active site over the course of the run and to compare its behavior with that of chlorpyrifos, a reference AChE inhibitor.

The MD trajectories generally corroborated the docking poses, maintaining key hydrogen bonds and hydrophobic interactions identified in the initial static models, while allowing assessment of their persistence over time. For this purpose, three main descriptors were monitored: the root mean square deviation (RMSD) to assess overall protein–ligand structural stability; the root mean square fluctuation (RMSF) to examine residue-specific mobility; and ligand–protein interaction histograms to track the frequency and durability of hydrogen bonds, hydrophobic contacts, and ionic interactions.

**Fig. 3:**
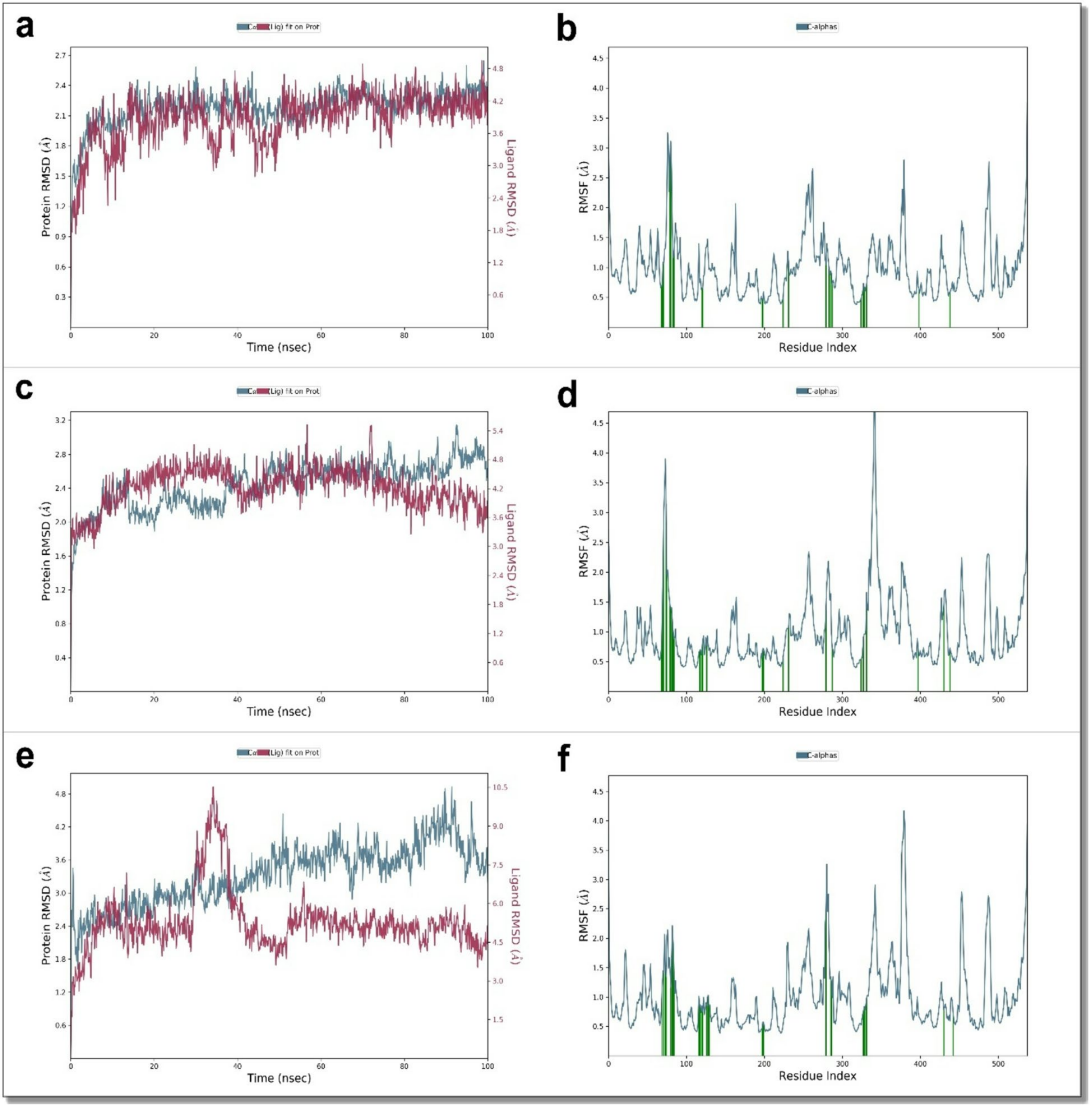
Comparative analysis of the dynamic stability and flexibility of acetylcholinesterase (AChE) in complex with the most active derivatives (**7** and **12**) and with the reference insecticide chlorpyrifos over a 100 ns molecular dynamics (MD) simulation. (a) RMSD plot showing the backbone stability of AChE (blue) and ligand RMSD (red) for derivative **7**. (b) RMSF profile of AChE residues in complex with derivative **7**, indicating residue-level fluctuations and regions of low flexibility. (c) RMSD plot showing the backbone stability of AChE (blue) and ligand RMSD (red) for derivative **12**. (d) RMSF profile of AChE residues in complex with derivative **12**, illustrating the flexibility distribution along the protein chain. (e) RMSD plot for the AChE–chlorpyrifos complex, showing protein backbone RMSD (blue) and ligand RMSD (red) across the same simulation period. (f) RMSF profile for AChE–chlorpyrifos, highlighting overall residue-wise dynamics. Overall, these plots demonstrate distinct differences in conformational stability and residue-level flexibility of AChE depending on the bound ligand, with derivatives **7** and **12** exhibiting more stable binding profiles than the reference compound.

RMSD analysis showed that the AChE backbone maintained overall structural stability throughout all simulations, indicating no large-scale conformational rearrangements upon ligand binding. The RMSD trajectories of the ligands, however, displayed subtle differences. Derivatives **7** and **12** exhibited slightly lower and more stable RMSD values compared with chlorpyrifos, suggesting a more consistent accommodation within the active site. In contrast, the chlorpyrifos–AChE complex showed transient fluctuations during the early phase of the simulation before reaching equilibrium, which may reflect a somewhat looser initial fit. Overall, all systems reached stable conformations within the first 20–25 ns of the trajectories (Fig. 3a, c,e).

RMSF analysis further supported these observations. As expected, the terminal regions of AChE displayed higher flexibility across all systems, while the residues forming the catalytic gorge exhibited relatively low fluctuations. The overall RMSF profiles of the derivative **7**, derivative **12**, and chlorpyrifos complexes were broadly similar, with derivatives **7** and **12** showing slightly reduced mobility at residues surrounding the active-site pocket. This modest local rigidity is consistent with more stable ligand anchoring rather than a major conformational restriction. By contrast, chlorpyrifos displayed marginally higher residue fluctuations in some regions, aligning with its comparatively weaker binding stability (Fig. 3b, d,f).

**Fig. 4:**
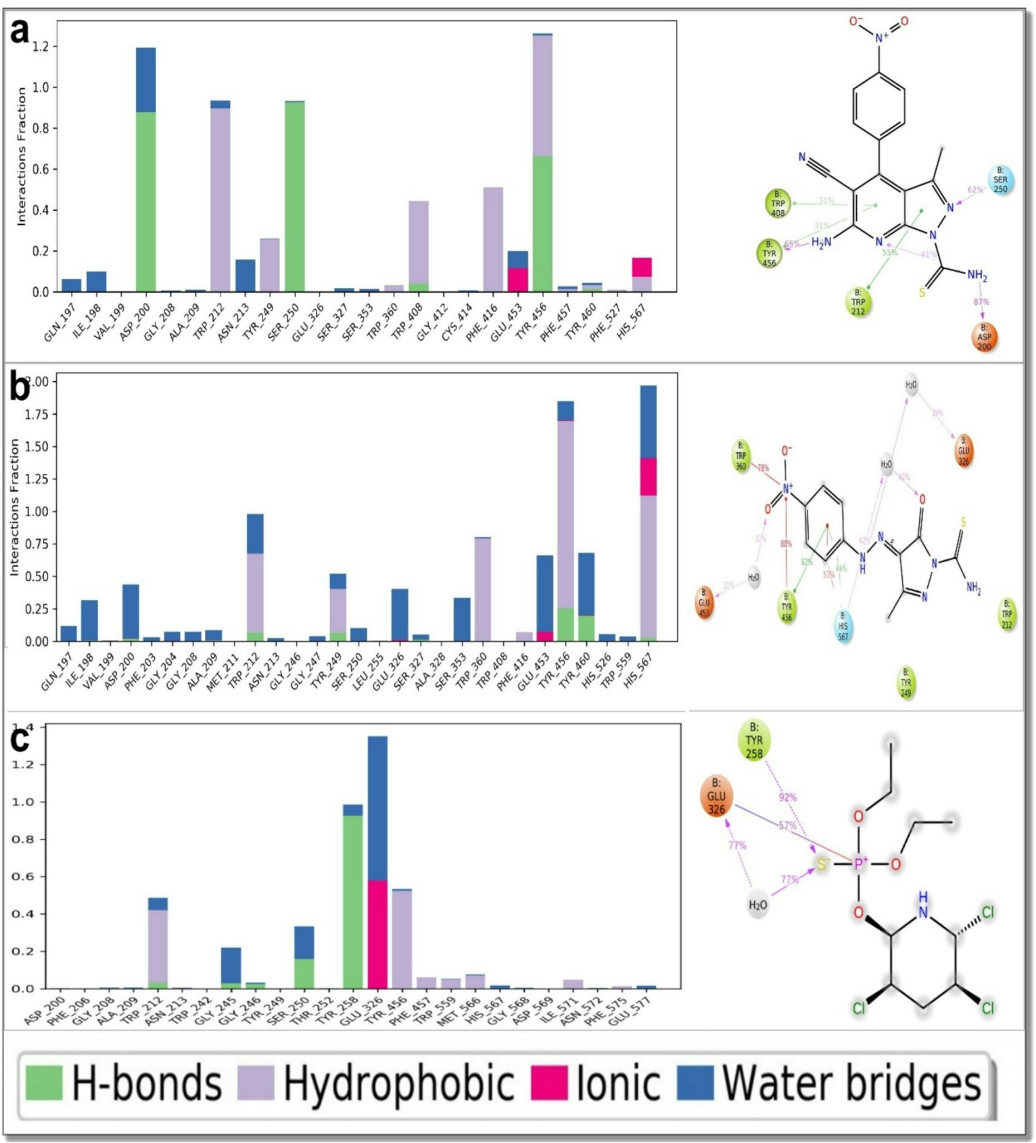
Molecular dynamics (MD) simulation analyses illustrating the protein–ligand interaction histograms and contact frequencies for the top-performing derivatives and the reference insecticide with acetylcholinesterase (AChE) from *Culex pipiens*. (a) derivative **7** — showing stable and recurrent hydrogen-bonding, hydrophobic, ionic, and water-bridge interactions that persist throughout the simulation. (b) Derivative **12** displays similar stability patterns with high-frequency hydrogen bonds and water-mediated contacts, indicating a comparable binding persistence within the active site. (c) Chlorpyrifos exhibits fewer and less stable interactions overall, consistent with its weaker binding profile. Overall, these interaction histograms emphasize the stronger and more stable binding behavior of derivatives **7** and **12** compared with chlorpyrifos, highlighting their potential as superior AChE-targeting larvicidal agents.

Ligand–protein interaction profiling provided further insight into the binding stability of the three complexes (Fig. 4). Both derivatives **7** and **12** maintained a balanced combination of hydrogen-bonding, hydrophobic, ionic, and water-mediated interactions throughout the simulation, with moderate persistence over time. These interactions collectively contributed to the observed complex stability in the MD trajectories. In contrast, chlorpyrifos showed a smaller number of persistent contacts and a greater reliance on transient water bridges, indicating comparatively weaker and less consistent binding. Notably, unlike the docking results, where the relative binding differences among the ligands were less pronounced, the MD simulations clearly highlighted the superior interaction stability and a higher number of sustained contacts for derivatives **7** and **12** compared with chlorpyrifos. This dynamic consistency likely underpins their higher experimental larvicidal potency and demonstrates the greater predictive reliability of molecular dynamics simulations in capturing subtle yet biologically relevant differences in binding strength and persistence.

This alignment between dynamic interaction behavior and experimental toxicity levels strengthens the interpretation that AChE inhibition is the primary driver of larval mortality, as predicted by the in-silico models. Collectively, the MD simulations reinforce the docking predictions, demonstrating that derivatives “**7**” and “**12**” forms a more stable and strongly interacting complexes with AChE than chlorpyrifos. The combination of low ligand RMSD, reduced RMSF at functionally critical residues, and sustained intermolecular contacts suggests that derivatives “**7**” and “**12**” are potent and persistent AChE binders, supporting their potential as effective insecticidal agents against *Culex pipiens* larvae. Although molecular dynamics simulations provide high-resolution insight and strongly support the docking results—indicating that larval mortality likely stems from acetylcholinesterase inhibition experimental enzymatic inhibition assays will be essential in future studies to validate these computational findings.

## Conclusion

This work highlights the potential of newly developed 3-methyl-pyrazole derivatives as highly effective larvicides against *Culex pipiens*. Using a rational design approach combined with chemical synthesis and thorough biological testing, several derivatives demonstrated remarkable insecticidal activity most notably derivatives **“7”** and **“12”** which outperformed the benchmark insecticide chlorpyrifos. Computational studies, including molecular docking and molecular dynamics, revealed that the most active compounds formed stable, high-affinity complexes with two primary neural targets: the enzyme responsible for acetylcholine breakdown (AChE) and the ligand-gated ion channel involved in cholinergic signaling (nAChR). These findings are consistent with a neurotoxic mode of action. Structure–activity relationship (SAR) analysis further identified key functional groups that enhance bioactivity, offering valuable guidance for future molecular optimization. Overall, the results support the strategy of disrupting mosquito neural pathways with novel chemical scaffolds and provide a strong basis for the development of next-generation, environmentally safe, and resistance-mitigating insecticides for vector control. Nevertheless, this study represents a preliminary stage in the discovery process. Further investigations are recommended, including enzyme-inhibition assays on acetylcholinesterase (AChE), comprehensive toxicological evaluations, and environmental-safety studies involving non-target organisms, to validate these findings and ensure ecological compatibility before any field application. It is also recommended that future research expand larvicidal screening to other medically important mosquito species, such as *Aedes* and *Anopheles*, followed by semi-field or field evaluations to confirm cross-species efficacy and assess practical feasibility under realistic conditions.

## Supplementary Information

Below is the link to the electronic supplementary material.

Supplementary Material 1

## Data Availability

All data generated or analyzed during this study are included in this published article and its supplementary information files.

## References

[1] Barba, M., Fairbanks, E. L. & Daly, J. M. Equine viral encephalitis: prevalence, impact, and management strategies. 10.2147/VMRR.S168227 (2019). 10.2147/VMRR.S168227 PMC6689664 31497528

[2] Vitek, C. J., Richards, S. L., Mores, C. N., Day, J. F. & Lord, C. C. Arbovirus transmission by Culex nigripalpus in Florida, 2005. *J. Med. Entomol.* **45**, 483 (2008). 18533444 10.1603/0022-2585(2008)45[483:atbcni]2.0.co;2 PMC2770802

[3] Hamer, G. L. et al. Host selection by Culex pipiens mosquitoes and west nile virus amplification. *Am. J. Trop. Med. Hyg.* **80**, 268–278. 10.4269/AJTMH.2009.80.268 (2009). 19190226

[4] Gorris, M. E. et al. Updated distribution maps of predominant Culex mosquitoes across the Americas. *Parasit. Vectors*. **14**, 1–13. 10.1186/s13071-021-05051-3 (2021). 34688314 10.1186/s13071-021-05051-3 PMC8542338

[5] Adly, E., Hegazy, A. A., Kamal, M. & Abu-Hussien, S. H. Midguts of Culex pipiens L. (Diptera: Culicidae) as a potential source of raw milk contamination with pathogens. *Sci. Rep.* **12**, 968. 10.1038/s41598-022-16992-9 (2022). 10.1038/s41598-022-16992-9 PMC9343664 35915127

[6] Tomlin, C. D. S. & Council, B. C. P. *The Pesticide Manual: A World Compendium* (Springer, 2006).

[7] Labbé, P. et al. Evolution of resistance to insecticide in disease vectors. *Evol. Resist. Insecticide Disease Vectors*. **14**, 686. 10.1016/B978-0-12-799942-5.00014-7 (2017).

[8] Liu, N. Insecticide resistance in mosquitoes: impact, mechanisms, and research directions. *Annu. Rev. Entomol.* **60**, 537–559. 10.1146/ANNUREV-ENTO-010814-020828 (2015). 25564745 10.1146/annurev-ento-010814-020828

[9] Mohamed, A. M. M., Ismail, M. F., Madkour, H. M. F., Aly, A. F. & Salem, M. S. Straightforward synthesis of 2-chloro-N-(5-(cyanomethyl)-1,3,4-thiadiazol-2-yl)benzamide as a precursor for synthesis of novel heterocyclic compounds with insecticidal activity. *Synth. Commun.* **50**, 3424–3442. 10.1080/00397911.2020.1802652 (2020).

[10] Ismail, M. F., Madkour, H. M. F., Salem, M. S., Mohamed, A. M. M. & Aly, A. F. Design, synthesis and insecticidal activity of new 1,3,4-thiadiazole and 1,3,4-thiadiazolo[3,2-a]pyrimidine derivatives under solvent-free conditions. *Synth. Commun.* **51**, 2644–2660. 10.1080/00397911.2021.1945106 (2021).

[11] Ismail, M. F., Aly, A. F., Abdel-Wahab, S. S. & El-Sayed, A. A. Synthesis, characterization and insecticidal activity against cotton leaf worm of new heterocyclics which scaffold on hydrazide-hydrazone derivative. *Polycycl. Aromat. Compd.* **43**, 1288–1308. 10.1080/10406638.2022.2026990 (2023).

[12] Ismail, M. F., Hashem, A. I., Sleem, R. A. & Hassaballah, A. I. Utility of 5,5′-Methylenebis(2-hydroxybenzaldehyde) – cyanoacetohydrazide hybrid as Scaffold for the synthesis of bis-heterocyclic moieties and study of their insecticidal activity. *ChemistrySelect* **8**, e202204946. 10.1002/SLCT.202204946 (2023).

[13] El-Sayed, A. A., Elsayed, G. A., Rizk, S. A. & Ismail, M. F. Synthesis insecticidal activity and DFT study of 1,2,4-Triazolidinthione, 1,3,5-oxadiazine and thiourea derivatives. *Org. Prep Proced. Int.* **56**, 38–51. 10.1080/00304948.2023.2209489 (2024).

[14] Abbass, E. M., Ali, A. K., El-Farargy, A. F., Abdel-Haleem, D. R. & Shaban, S. S. Synthesis, toxicological and in silico evaluation of novel spiro pyrimidines against Culex pipiens L. referring to chitinase enzyme. *Sci. Rep.* **14**, 1–17. 10.1038/S41598-024-51771-8 (2024). 38233515 10.1038/s41598-024-51771-8 PMC10794250

[15] Rady, M. H. et al. Potential establishment, prevalence of dengue vector, Aedes sp. and its risk map in Hurghada region, Red Sea, Egypt. *Egypt. J. Aquat. Biol. Fish.* **28**, 399. 10.21608/EJABF.2024.378883 (2024).

[16] Abouelhassan, E. M. et al. Molecular identification and morphological variations of Amblyomma lepidum imported to Egypt, with notes about its potential distribution under climate change. *Parasitol. Res.* **123**, 1–13. 10.1007/S00436-024-08284-0 (2024). 10.1007/s00436-024-08284-0 PMC11255089 39017762

[17] Okely, M., Chen, Z., Adly, E. & Kamal, M. Climate change influences on the potential geographic distribution of the invasive Asian longhorned tick, Haemaphysalis longicornis. *Sci. Rep. 2025*. **15**, 1. 10.1038/s41598-025-86205-6 (2025). 10.1038/s41598-025-86205-6 PMC11748616 39824882

[18] Kamal, M., Kenawy, M. A., Rady, M. H., Khaled, A. S. & Samy, A. M. Mapping the global potential distributions of two arboviral vectors Aedes aegypti and Ae. Albopictus under changing climate. *PLoS One*. **13**, 96. 10.1371/journal.pone.0210122 (2018). 10.1371/journal.pone.0210122 PMC6312308 30596764

[19] Hosni, E. M., Al-Khalaf, A. A., Nasser, M. G., ElShahed, S. M. & Alashaal, S. A. Locusta migratoria (L.) (Orthoptera) in a warming world: unravelling the ecological consequences of climate change using GIS. *Biodivers. Data J.* **12**, e115845. 10.3897/BDJ.12.E115845 (2024). E115845 12. 38481856 10.3897/BDJ.12.e115845 PMC10933582

[20] ElShahed, S. M., Mostafa, Z. K., Radwan, M. H. & Hosni, E. M. Modeling the potential global distribution of the Egyptian cotton leafworm, Spodoptera littoralis under climate change. *Sci. Rep.* **13**, 2563. 10.1038/S41598-023-44441-8 (2023). 10.1038/s41598-023-44441-8 PMC10570271 37828108

[21] Al-Khalaf, A. A., Nasser, M. G. & Hosni, E. M. Global potential distribution of Sarcophaga dux and Sarcophaga haemorrhoidalis under climate change. *Divers. (Basel)*. **15**, 903. 10.3390/D15080903 (2023).

[22] Achee, N. L. et al. A critical assessment of vector control for dengue prevention. *PLoS Negl. Trop. Dis.* **9**, e0003655. 10.1371/JOURNAL.PNTD.0003655 (2015). 25951103 10.1371/journal.pntd.0003655 PMC4423954

[23] Hemingway, J. et al. Averting a malaria disaster: will insecticide resistance derail malaria control? *Lancet* **387**, 1785–1788. 10.1016/S0140-6736(15)00417-1 (2016). 26880124 10.1016/S0140-6736(15)00417-1 PMC6215693

[24] Ranson, H. et al. Pyrethroid resistance in African anopheline mosquitoes: what are the implications for malaria control? *Trends Parasitol.* **27**, 91–98. 10.1016/J.PT.2010.08.004 (2011). 20843745 10.1016/j.pt.2010.08.004

[25] ffrench-Constant, R. H., Williamson, M. S., Davies, T. G. E. & Bass, C. Ion channels as insecticide targets. *J. Neurogenet.* **30**, 163. 10.1080/01677063.2016.1229781 (2016). 27802784 10.1080/01677063.2016.1229781 PMC6021766

[26] Sparks, T. C. et al. Insecticides, biologics and nematicides: updates to IRAC’s mode of action classification - a tool for resistance management. *Pestic Biochem. Physiol.* **167**, 104587. 10.1016/J.PESTBP.2020.104587 (2020). 32527435 10.1016/j.pestbp.2020.104587

[27] Pang, Y. P. Insect acetylcholinesterase as a target for effective and environmentally safe insecticides. *Adv. Insect Phys.* **46**, 435–494. 10.1016/B978-0-12-417010-0.00006-9 (2014).

[28] Sparks, T. C. & Nauen, R. Mode of action classification and insecticide resistance management. *Pestic Biochem. Physiol.* **121**, 122–128. 10.1016/J.PESTBP.2014.11.014 (2015). 26047120 10.1016/j.pestbp.2014.11.014

[29] Casida, J. E. & Durkin, K. A. Neuroactive insecticides: targets, selectivity, resistance, and secondary effects. *Annu. Rev. Entomol.* **58**, 99–117. 10.1146/ANNUREV-ENTO-120811-153645 (2013). 23317040 10.1146/annurev-ento-120811-153645

[30] Haikal, A., Kamal, M., Hosni, E. M. & Amen, Y. Evaluation of hesperidin as a potential larvicide against Culex pipiens with computational prediction of its mode of action via molecular docking. *Sci. Rep. ***15**, 1. 10.1038/s41598-025-85760-2 (2025). 39837950 10.1038/s41598-025-85760-2 PMC11751293

[31] Khalil, M. S. et al. Synthesis and insecticidal assessment of nitrogenous heterocycles derived from 2-pyridone derivative against Culex pipiens L. Larvae. *J. Mol. Struct.* **1322**, 140405. 10.1016/J.MOLSTRUC.2024.140405 (2025).

[32] El-Sayed, M. K. F. et al. Synthesis, molecular modelling and evaluation of larvicidal efficacy of annulated Benzo[h]chromenes against Culex pipiens L. *Sci. Rep.* **14**, 1–19. 10.1038/s41598-024-68035-0 (2024). 10.1038/s41598-024-68035-0 PMC11310521 39117743

[33] Shah, R. M. et al. Toxicity of 25 synthetic insecticides to the field population of Culex quinquefasciatus Say. *Parasitol. Res.* **115**, 4345–4351. 10.1007/S00436-016-5218-8 (2016). 27530515 10.1007/s00436-016-5218-8

[34] Kauffman, E. et al. Rearing of Culex spp. and Aedes spp. *Mosquitoes Bio-Protocol*. **7**, 2542. 10.21769/BioProtoc.2542 (2017). 10.21769/BioProtoc.2542 PMC5654580 29075656

[35] World Health Organization, Guidelines for laboratory and field testing of mosquito larvicides (2005). https://www.WHO/CDS/WHOPES/GCDPP/2005.13.

[36] Ismail, M. F., El-Sayed, A. A., Hosni, E. M. & Hassaballah, A. I. Synthesis and evaluation of larvicidal efficacy against C. pipiens of some new heterocyclic compounds emanated from 2-cyano-N’-(2-(2,4-dichlorophenoxy)acetyl)acetohydrazide. *Chem. Biodivers.* **21**, 896. 10.1002/CBDV.202301560 (2024). 10.1002/cbdv.202301560 38251927

[37] Abbott, W. S. A method of computing the effectiveness of an insecticide. *J. Econ. Entomol.* **18**, 265–267. 10.1093/JEE/18.2.265A (1925).

[38] Finney, D. J. *Probit Analysis* (Cambridge University Press, 1971). 10.1002/JPS.2600600940.

[39] Sun, Y. P. Toxicity index-an improved method of comparing the relative toxicity of insecticides1. *J. Econ. Entomol.* **43**, 45–53. 10.1093/JEE/43.1.45 (1950).

[40] Bateman, A. UniProt: a worldwide hub of protein knowledge. *Nucleic Acids Res.* **47**, D506–D515. 10.1093/nar/gky1049 (2019). 30395287 10.1093/nar/gky1049 PMC6323992

[41] Waterhouse, A. et al. SWISS-MODEL: homology modelling of protein structures and complexes. *Nucleic Acids Res.* **46**, W296–W303. 10.1093/nar/gky427 (2018). 10.1093/nar/gky427 PMC6030848 29788355

[42] Guex, N., Peitsch, M. C. & Schwede, T. Automated comparative protein structure modeling with SWISS-MODEL and Swiss-PdbViewer: a historical perspective. *Electrophoresis 30 Suppl.*10.1002/ELPS.200900140 (2009). 10.1002/elps.200900140 19517507

[43] Bienert, S. et al. The SWISS-MODEL Repository-new features and functionality. *Nucleic Acids Res.* **45**, D313–D319. 10.1093/NAR/GKW1132 (2017). 27899672 10.1093/nar/gkw1132 PMC5210589

[44] Abd-Alsalam, E. et al. Exploring the Antiproliferative potency of pyrido[2,3-d]pyrimidine derivatives: studies on design, synthesis, anticancer evaluation, SAR, docking, and DFT calculations. *Chem. Biodivers.* **21**, 562. 10.1002/CBDV.202301682 (2024). 10.1002/cbdv.202301682 38084395

[45] Abbass, E. M., Khalil, A. K., Abdel-Mottaleb, Y. & Abdel-Mottaleb, M. S. A. Exploiting modeling studies for evaluating the potential antiviral activities of some clinically approved drugs and herbal materials against SARS-CoV-2: theoretical studies toward hindering the virus and blocking the human cellular receptor. *Polycycl. Aromat. Compd.* **44** 1209–1220. 10.1080/10406638.2023.2189736 (2024).

[46] Nofal, H. R. et al. Pharmacophore-based, rationale design, and efficient synthesis of novel tetrahydrobenzo[b]thiophene candidates as potential dual Topo I/II inhibitors and DNA intercalators. *Arch. Pharm. (Weinheim)* **2024**, 357. 10.1002/ARDP.202400217 (2024). 10.1002/ardp.202400217 38864845

[47] Rao, P., Goswami, D. & Rawal, R. M. Revealing the molecular interplay of curcumin as Culex pipiens Acetylcholine esterase 1 (AChE1) inhibitor. *Sci. Rep.* **11**, 256. 10.1038/s41598-021-96963-8 (2021). 10.1038/s41598-021-96963-8 PMC8410813 34471175

[48] El-Helw, E. A. E., Hosni, E. M., Kamal, M., Hashem, A. I. & Ramadan, S. K. Synthesis, insecticidal activity, and molecular docking analysis of some benzo[h]quinoline derivatives against Culex pipiens L. Larvae. *Bioorg. Chem.* **2024**, 107591. 10.1016/J.BIOORG.2024.107591 (2024). 10.1016/j.bioorg.2024.107591 38964147

[49] Ramadan, S. K. et al. Synthesis, in vivo evaluation, and in silico molecular docking of benzo[h]quinoline derivatives as potential Culex pipiens L. larvicides. *Bioorg. Chem.* **154**, 108090. 10.1016/J.BIOORG.2024.108090 (2025). 39742674 10.1016/j.bioorg.2024.108090

[50] Hassaballah, A. I. et al. Design synthesis, characterization, molecular docking and antimicrobial evaluation of novel heterocycles with acrylonitrile and anthracene moieties. *Sci. Rep. 2025*. **15** (15), 1. 10.1038/s41598-025-03272-5 (2025). 10.1038/s41598-025-03272-5 PMC12134333 40461523

[51] Rashad, A. A. et al. Aquatic larvicidal efficacy of periplaneta americana chitin and essential oils against culex pipiens: a comparative assessment. *Egypt. J. Aquat. Biol. Fish.* **29**, 2911–2938. 10.21608/EJABF.2025.434231 (2025).

[52] El-Helw, E. A. E. et al. Synthesis of Benzo[h]quinoline derivatives and evaluation of their insecticidal activity against Culex pipiens L. larvae. *Eur. J. Med. Chem.* **2025**, 117565. 10.1016/J.EJMECH.2025.117565 (2025). 10.1016/j.ejmech.2025.117565 40153929

[53] Bondock, S., El-Azap, H., Kandeel, E. E. M. & Metwally, M. A. Eco-friendly solvent-free synthesis of thiazolylpyrazole derivatives. *Monatsh Chem.* **139**, 1329–1335. 10.1007/S00706-008-0930-4 (2008).

[54] Casas, J. S. et al. Reactions of cadmium(II) acetate with acetoacetanilide and methylacetoacetate thiosemicarbazones: a cyclization process leading to a pyrazolone – the molecular and crystal structures of the free ligands and the complex [CdL2Py] (HL = 2-[Amino(thioxo)methyl]-5-methyl-2,3-dihydro-1H-3-pyrazolone, Py = Pyridine). *Eur. J. Inorg. Chem.* **2000**, 83–89. 10.1002/(SICI)1099-0682(200001)2000:1<83::AID-EJIC83>3.0.CO;2-9 (2000).

[55] Kiran, V. & Joshi, R. Pundeer, α,α-Dibromoketone precursors in the synthesis of some new thiazole derivatives: thiazol-2-yl hydrazonobutanoates, thiazol-2-yl pyrazole-4-carboxylates and acids. *J. Heterocycl. Chem.* **57**, 2173–2183. 10.1002/JHET.3937 (2020).

[56] Mohd Amir, R. A. Synthesis and antibacterial activity of 1-Thiocarbamoyl-3-methyl-4-(arylhydrazono)-2-pyrazolin-5-one. 10.5281/ZENODO.5876022 (2025).

[57] Sharma, V. K. & Singh, S. K. Synthesis, utility and medicinal importance of 1,2- & 1,4-dihydropyridines. *RSC Adv.* **7**, 2682–2732. 10.1039/C6RA24823C (2017).

[58] Daaboub, J., Tabbabi, A., Ben Cheikh, R., Lamari, A. & Ibtissem, B. Susceptibility of larval stage of mosquito Culex pipiens against chlorpyrifos insecticide in Southern Tunisia. *Hereditary Genet.*10.4172/2161-1041.1000182 (2017).

[59] Talipouo, A. et al. High insecticide resistance mediated by different mechanisms in Culex quinquefasciatus populations from the city of Yaoundé, Cameroon. *Sci. Rep.* **11**, 1–11. 10.1038/S41598-021-86850-7 (2021). 33795804 10.1038/s41598-021-86850-7 PMC8017000

[60] Yoo, D. H. et al. Insecticide susceptibility of field-collected populations of Culex tritaeniorhynchus in the Republic of Korea. *J. Insect Sci.* **13**, 2. 10.1673/031.013.0201 (2013). 23879898 10.1673/031.013.0201 PMC3735112

[61] Li, C. X. et al. Identification of genes involved in pyrethroid-, propoxur-, and dichlorvos- insecticides resistance in the mosquitoes, Culex pipiens complex (Diptera: Culicidae). *Acta Trop.* **157**, 84–95. 10.1016/J.ACTATROPICA.2016.01.019 (2016). 26802491 10.1016/j.actatropica.2016.01.019

[62] Liu, H. et al. Trends in insecticide resistance in Culex pipiens pallens over 20 years in Shandong, China. *Parasit. Vectors*. **12**, 1–9. 10.1186/S13071-019-3416-9 (2019). 30975185 10.1186/s13071-019-3416-9 PMC6460514

[63] Pasteur, N., Marquine, M., Ben Cheikh, H., Bernard, C. & Bourguet, D. A new mechanism conferring unprecedented high resistance to chlorpyrifos in Culex pipiens (Diptera: Culicidae). *J. Med. Entomol.* **36**, 794–802. 10.1093/JMEDENT/36.6.794 (1999). 10593083 10.1093/jmedent/36.6.794

[64] Alout, H. et al. High chlorpyrifos resistance in Culex pipiens mosquitoes: strong synergy between resistance genes. *Heredity (Edinb)*. **116**, 224–231. 10.1038/HDY.2015.92 (2016). 26463842 10.1038/hdy.2015.92 PMC4806891

[65] Daaboub, J., Tabbabi, A., Lamari, A., Ben Cheikh, R. & Ben Jha, I. Evaluation of chlorpyrifos resistance and biochemical mechanisms of culex pipiens in five localities of grand Tunis Area, Northeast Tunisia. *Hereditary Genet.*10.4172/2161-1041.1000183 (2017).

[66] Waiskopf, N. & Soreq, H. Cholinesterase inhibitors: from molecular mechanisms of action to current and future prospects. from molecular mechanisms of action to current and future prospects. *Handb. Toxicol. Chem. Warfare Agents: Second Edition* **2015**, 761–778. 10.1016/B978-0-12-800159-2.00052-X (2015).

[67] Rust, M. K., Waggoner, M. M., Hinkle, N. C., Stansfield, D. & Barnett, S. Efficacy and longevity of nitenpyram against adult cat fleas (Siphonaptera: Pulicidae). *J. Med. Entomol.* **40**, 678–681. 10.1603/0022-2585-40.5.678 (2003). 14596282 10.1603/0022-2585-40.5.678

[68] Ihara, M. et al. Crystal structures of Lymnaea stagnalis AChBP in complex with neonicotinoid insecticides imidacloprid and clothianidin. *Invertebr. Neurosci.* **8**, 71–81. 10.1007/S10158-008-0069-3 (2008). 10.1007/s10158-008-0069-3 PMC2413115 18338186

[69] Duan, H. et al. A novel halogen bond and a better-known hydrogen bond cooperation of neonicotinoid and insect nicotinic acetylcholine receptor recognition. *J. Mol. Model.* **18**, 3867–3875. 10.1007/S00894-012-1393-4 (2012). 22426511 10.1007/s00894-012-1393-4

[70] Kamal, M. et al. Synthesis and in silico studies of new thiophene-isoquinolinone hybrids as potential larvicides against Culex pipiens. *Sci. Rep.* **15**, 1–24. 10.1038/S41598-025-13063-7 (2025). 40745435 10.1038/s41598-025-13063-7 PMC12314032

[71] Haneen, D. S. A. et al. Synthesis, comprehensive in silico studies, and cytotoxicity evaluation of novel quinazolinone derivatives as potential anticancer agents. *Sci. Rep.* **15**, 1–25. 10.1038/S41598-025-08062-7 (2025). 40610494 10.1038/s41598-025-08062-7 PMC12229541

