# Supplementary material for: Design, synthesis, and insecticidal potency of novel 3-methyl-pyrazole derivatives against Culex pipiens larvae
Source: Sci Rep. 2026 May 9;16:14699. doi: 10.1038/s41598-026-50895-3 (PMC13157491; doi:10.1038/s41598-026-50895-3)

**Design, Synthesis, and Insecticidal Potency of Novel 3-Methyl-Pyrazole Derivatives Against *Culex pipiens* Larvae**

**Hager R. Nofal^1^, Ali Khalil Ali^1^, Mahmoud F. Ismail^1^, Mahmoud Kamal^2,*^, Eslam M. Hosni^2^, Eslam M. Abbass^1,*^**

^1^ Chemistry Department, Faculty of Science, Ain Shams University, Cairo, 11566, Egypt.

^2^ Entomology Department, Faculty of Science, Ain Shams University, Cairo, 11566, Egypt

* Corresponding Author:

**Eslam M. Abbass**

**ORCID**:<https://orcid.org/0000-0002-3245-1653>

**Email**: [eslammorad@sci.asu.edu.eg](mailto:eslammorad@sci.asu.edu.eg)

**Table of Contents**

| **Title** | **page** |
| --- | --- |
| **IR spectral data of the target compounds (1-20)** | **S3** |
| **Figure S1.** **IR** spectrum of compound **1** | S3 |
| **Figure S2.** **IR** spectrum of compound **2** | S4 |
| **Figure S3.** **IR** spectrum of compound **3** | S5 |
| **Figure S4.** **IR** spectrum of compound **4** | S6 |
| **Figure S5.** **IR** spectrum of compound **5** | S7 |
| **Figure S6.** **IR** spectrum of compound **6** | S8 |
| **Figure S7.** **IR** spectrum of compound **7** | S9 |
| **Figure S8.** **IR** spectrum of compound **8** | S10 |
| **Figure S9.** **IR** spectrum of compound **9** | S11 |
| **Figure S10.** **IR** spectrum of compound **10** | S12 |
| **Figure S11.** **IR** spectrum of compound **11** | S13 |
| **Figure S12.** **IR** spectrum of compound **12** | S14 |
| **Figure S13.** **IR** spectrum of compound **13** | S15 |
| **Figure S14.** **IR** spectrum of compound **14** | S16 |
| **Figure S15.** **IR** spectrum of compound **15** | S17 |
| **Figure S16.** **IR** spectrum of compound **16** | S18 |
| **Figure S17.** **IR** spectrum of compound **17** | S19 |
| **Figure S18.** **IR** spectrum of compound **18** | S20 |
| **Figure S19.** **IR** spectrum of compound **19** | S21 |
| **^1^H NMR spectral data of the target compounds** | **S22** |
| **Figure S21.** ^1^H NMR (500 MHz, DMSO-*d_6_*) spectrum of compound **2** | S22 |
| **Figure S22.** ^1^H NMR (500 MHz, DMSO-*d_6_*) spectrum of compound **4** | S23 |
| **Figure S23.** ^1^H NMR (500 MHz, DMSO-*d_6_*) spectrum of compound **5** | S24 |
| **Figure S24.** ^1^H NMR (500 MHz, DMSO-*d_6_*) spectrum of compound **6** | S25 |
| **Figure S25.** ^1^H NMR (500 MHz, DMSO-*d_6_*) spectrum of compound **7** | S26 |
| **Figure S26.** ^1^H NMR (500 MHz, DMSO-*d_6_*) spectrum of compound **8** | S27 |
| **Figure S27.** ^1^H NMR (500 MHz, DMSO-*d_6_*) spectrum of compound **9** | S28 |
| **Figure S28.** ^1^H NMR (500 MHz, DMSO-*d_6_*) spectrum of compound **10** | S29 |
| **Figure S29.** ^1^H NMR (500 MHz, DMSO-*d_6_*) spectrum of compound **11** | S30 |
| **Figure S30.** ^1^H NMR (500 MHz, DMSO-*d_6_*) spectrum of compound **14** | S31 |
| **Figure S31.** ^1^H NMR (500 MHz, DMSO-*d_6_*) spectrum of compound **15** | S32 |
| **Figure S32.** ^1^H NMR (500 MHz, DMSO-*d_6_*) spectrum of compound **16** | S33 |
| **Figure S33.** ^1^H NMR (500 MHz, DMSO-*d_6_*) spectrum of compound **17** | S34 |
| **Figure S34.** ^1^H NMR (500 MHz, DMSO-*d_6_*) spectrum of compound **18** | S35 |
| **Mass fragmentation of the target compounds** | **S36** |
| **Figure 35.** Mass fragmentation of compound **4** | S36 |
| **Figure 36.** Mass fragmentation of compound **5** | S37 |
| **Figure 37.** Mass fragmentation of compound **6** | S38 |
| **Figure 38.** Mass fragmentation of compound **7** | S39 |
| **Figure 39.** Mass fragmentation of compound **8** | S40 |
| **Figure 40.** Mass fragmentation of compound **9** | S41 |
| **Figure 41.** Mass fragmentation of compound **10** | S42 |
| **Figure 42.** Mass fragmentation of compound **11** | S43 |
| **Figure 43.** Mass fragmentation of compound **14** | S44 |
| **Figure 44.** Mass fragmentation of compound **15** | S45 |
| **Figure 45.** Mass fragmentation of compound **16** | S46 |
| **Figure 46.** Mass fragmentation of compound **17** | S47 |
| **Figure 47.** Mass fragmentation of compound **18** | S48 |
| **Biological evaluation** |  |
| **Figure S47.** Quality estimate parameters for modeled Acetylcholine Esterase (AChE) protein. | S49 |
| **Figure S48.** Quality estimate parameters for modeled nicotinic acetylcholine receptor. | S50 |
| **Figure S49.** AlphaFold Model of the Nicotinic Acetylcholine Receptor (nAChR) from Culex quinquefasciatus | S51 |
| **Table S1.** 2D interaction profiles of the 19 synthesized compounds targeting acetylcholinesterase (AChE) in *Culex pipiens*, compared with the conventional AChE inhibitor chlorpyrifos | S52 |
| **Table S2.** Molecular docking interaction profiles of 19 synthesized compounds with acetylcholinesterase (AChE) in *Culex pipiens* | S56 |
| **Table S3.** 2D interaction profiles of the 19 synthesized compounds targeting the nicotinic acetylcholine receptor (nAChR) in *Culex pipiens* | S60 |
| **Table S4.** Molecular docking interaction profiles of 19 synthesized compounds with the nicotinic acetylcholine receptor (nAChR) in *Culex pipiens* | S64 |
| **Ethical Approval** | S66 |

**IR spectral data of the target compounds (1-20)**


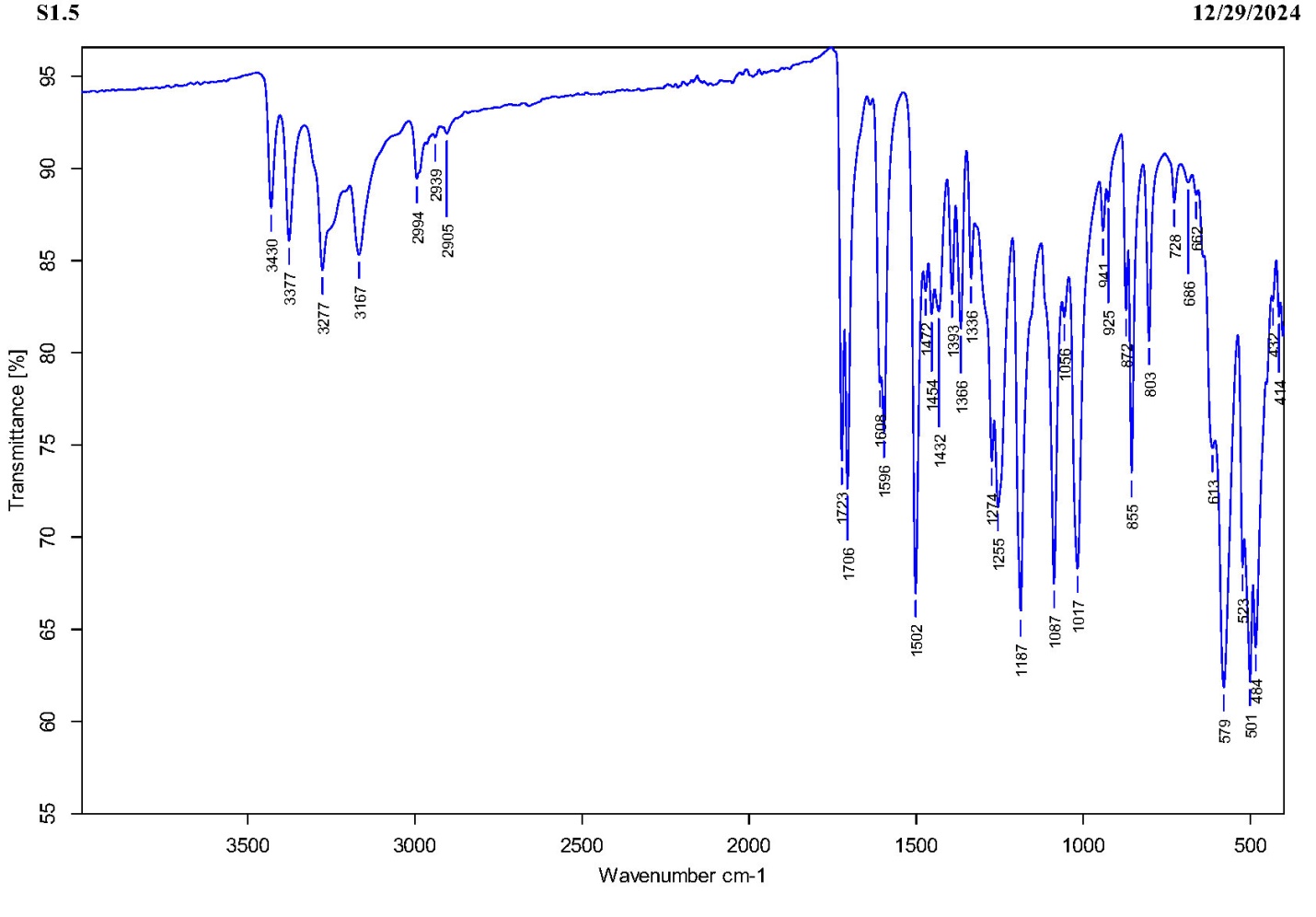


**Figure S1.** **IR** spectrum of compound **1.**


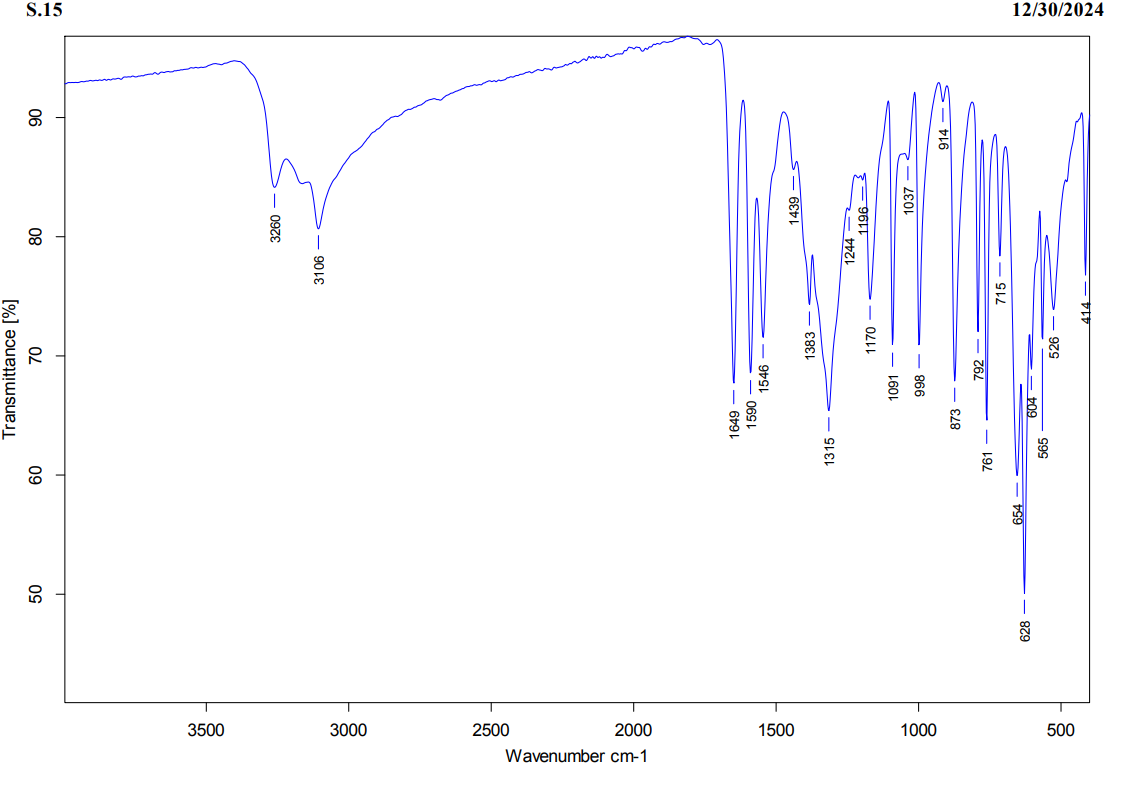


**Figure S2.** **IR** spectrum of compound **2**


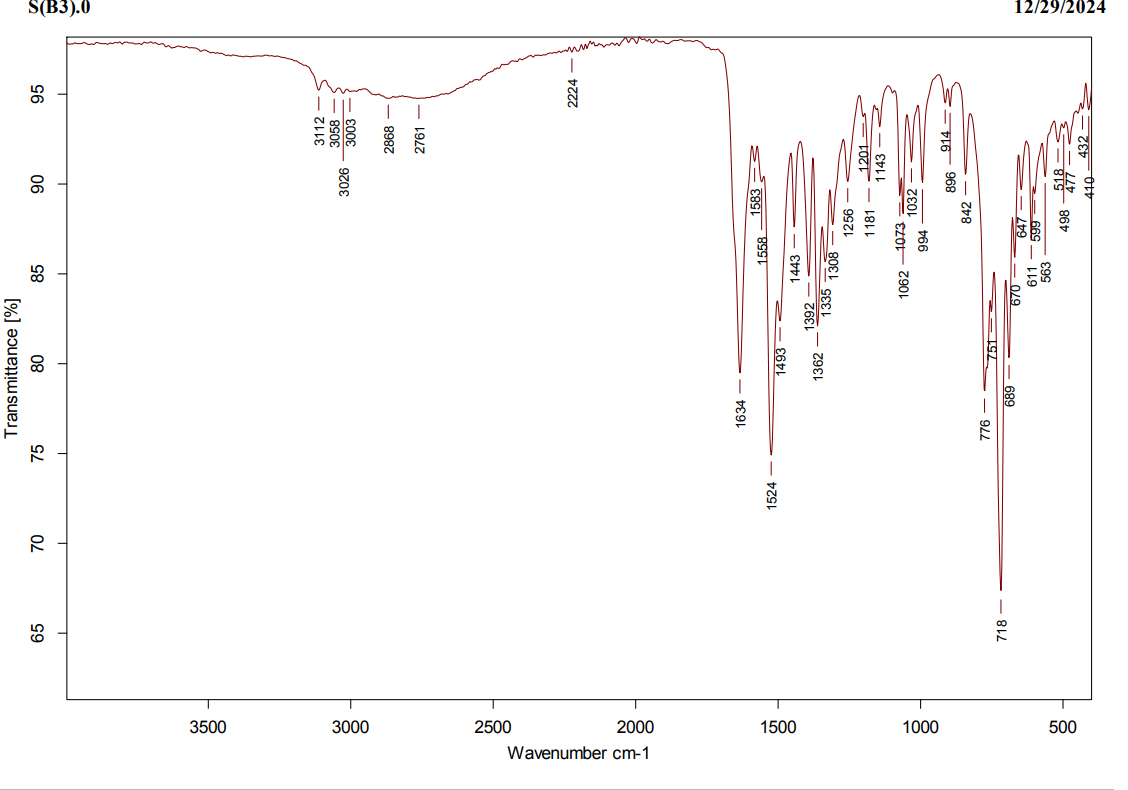


**Figure S3.** **IR** spectrum of compound **3**


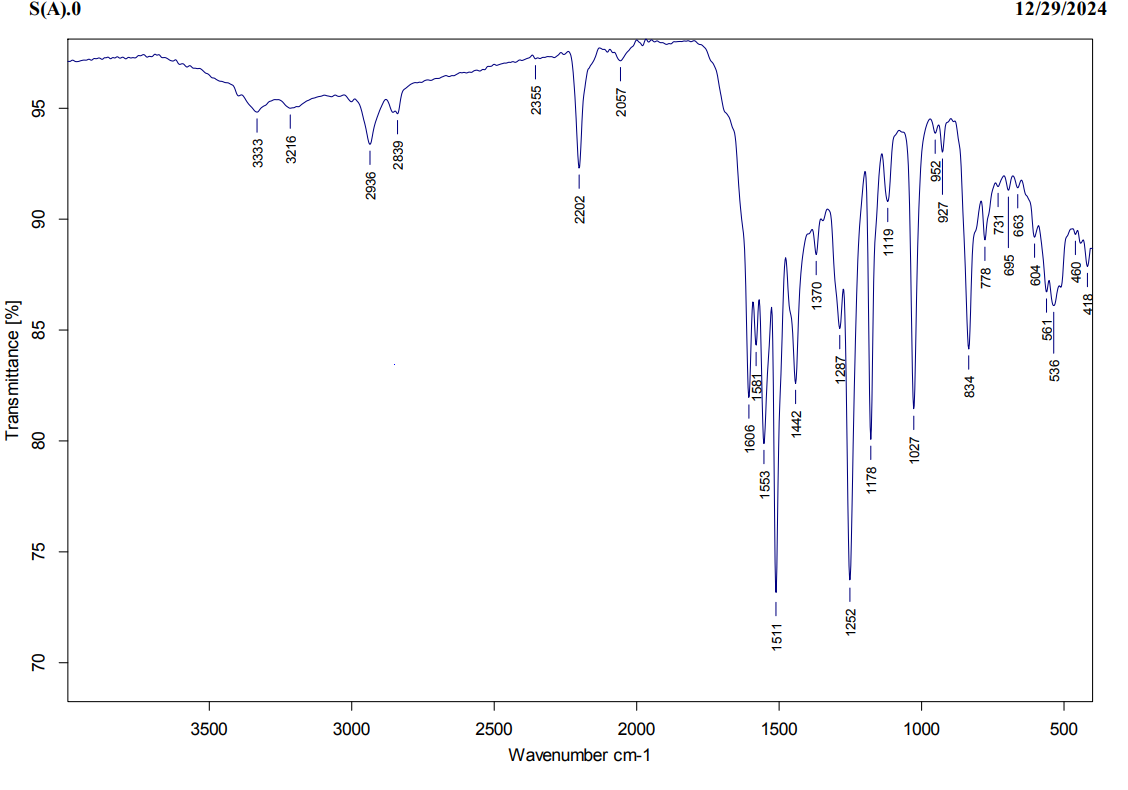


**Figure S4.** **IR** spectrum of compound **4**


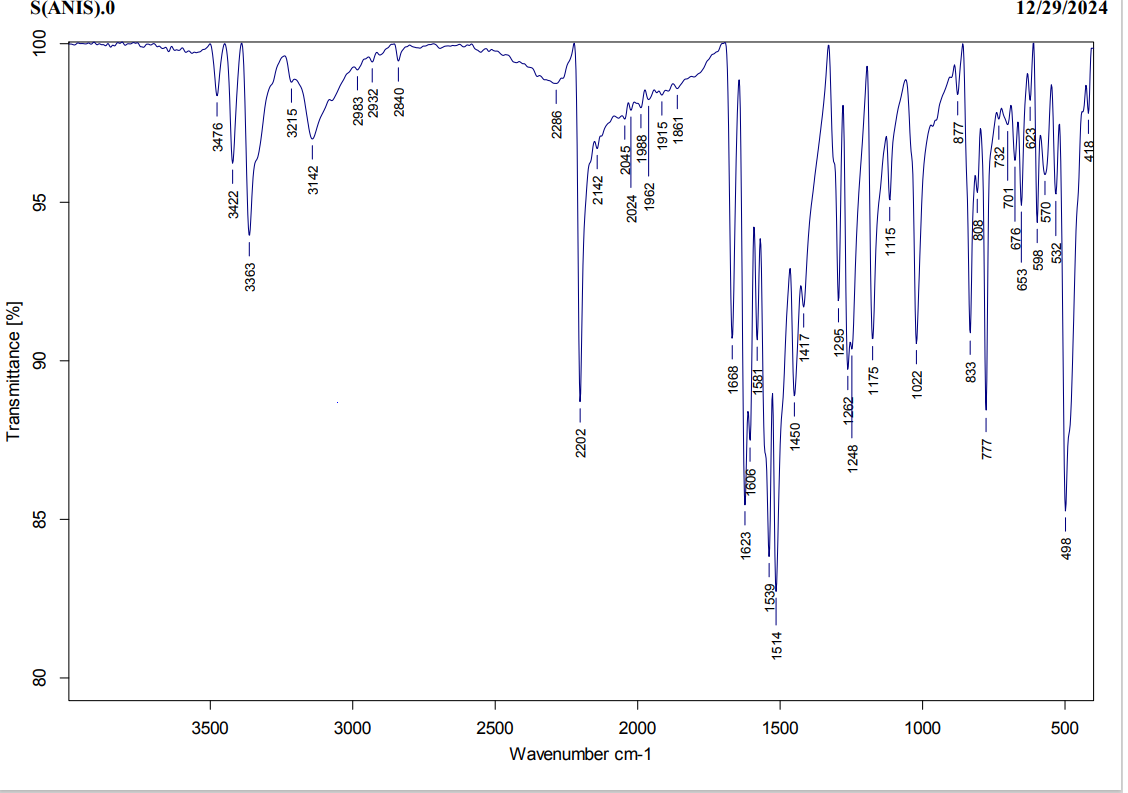


**Figure S5.** **IR** spectrum of compound **5**


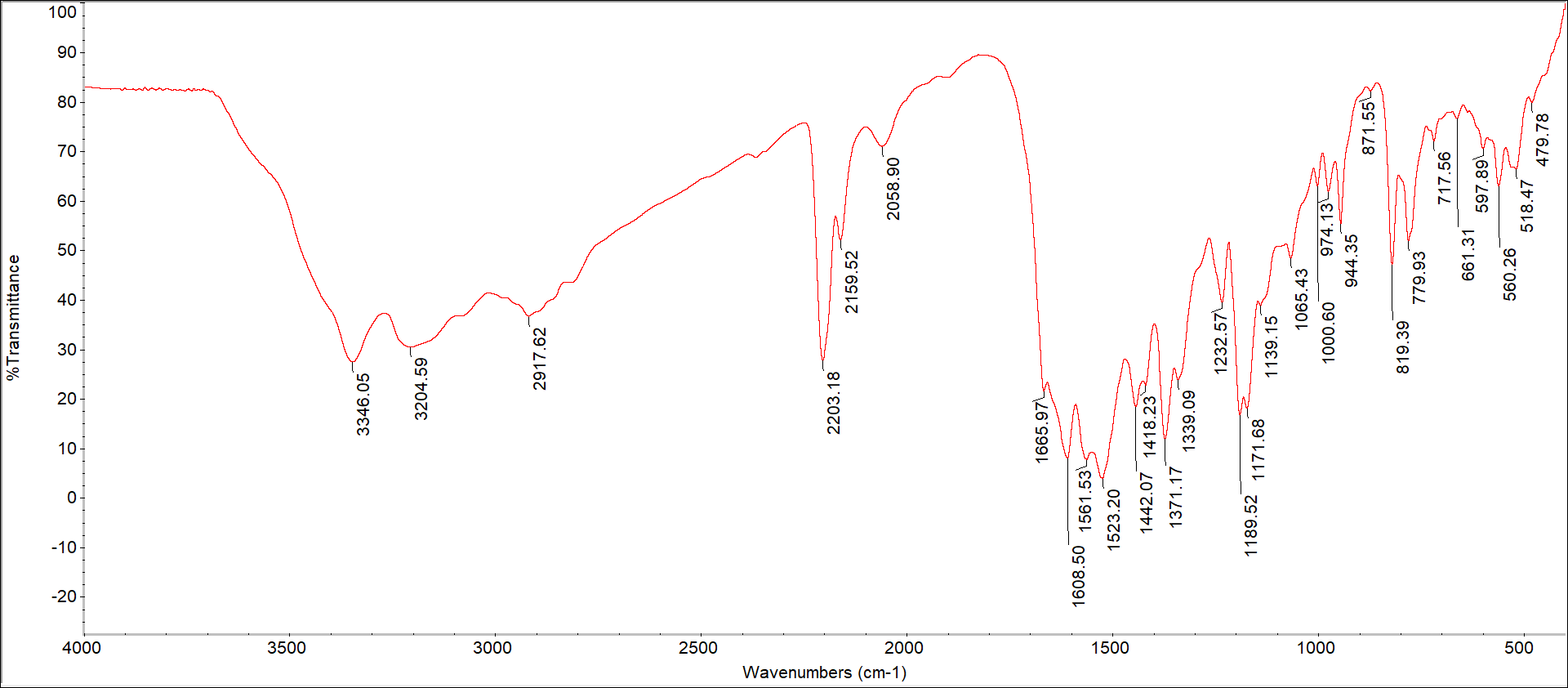


**Figure S6.** **IR** spectrum of compound **6**


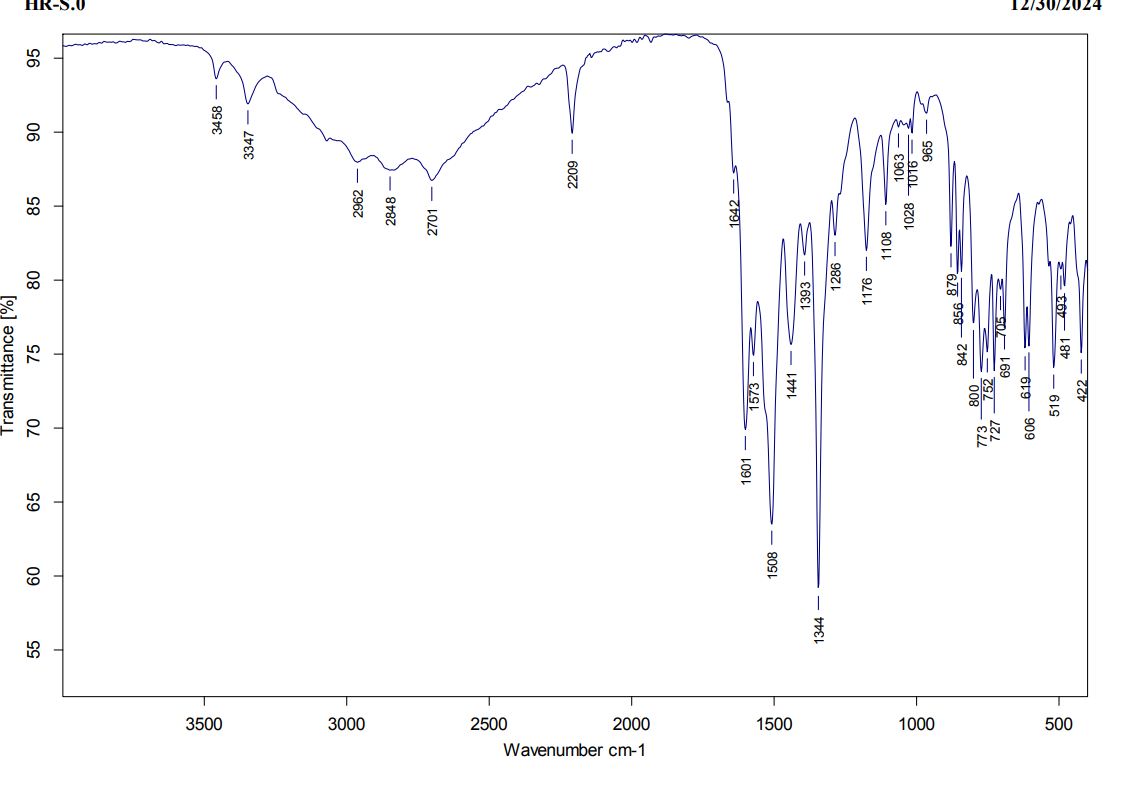


**Figure S7.** **IR** spectrum of compound **7**


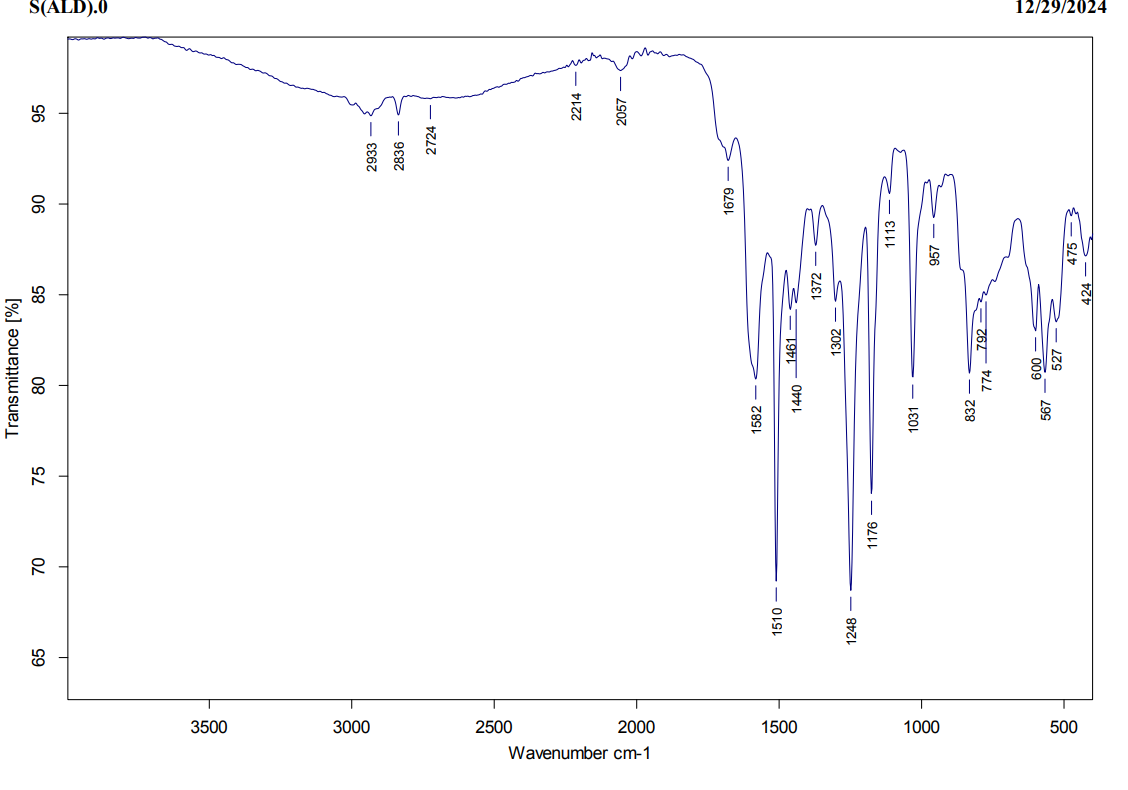


**Figure S8.** **IR** spectrum of compound **8**


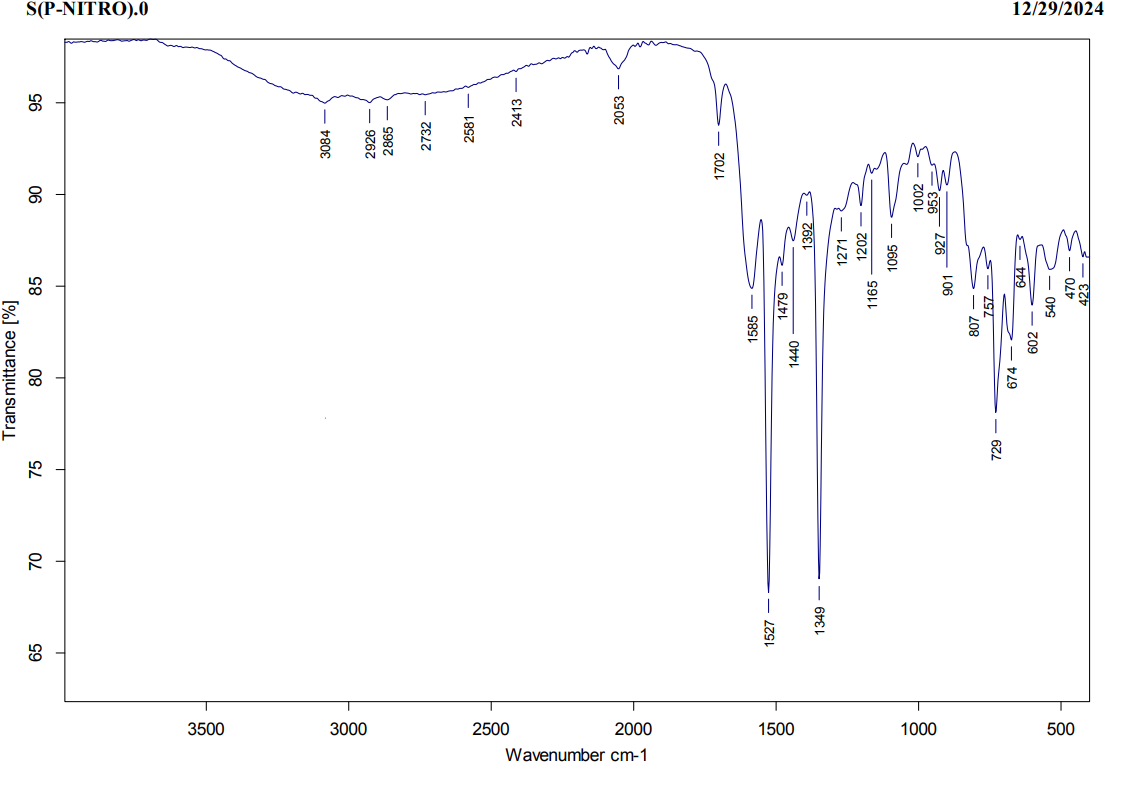


**Figure S9.** **IR** spectrum of compound **9**


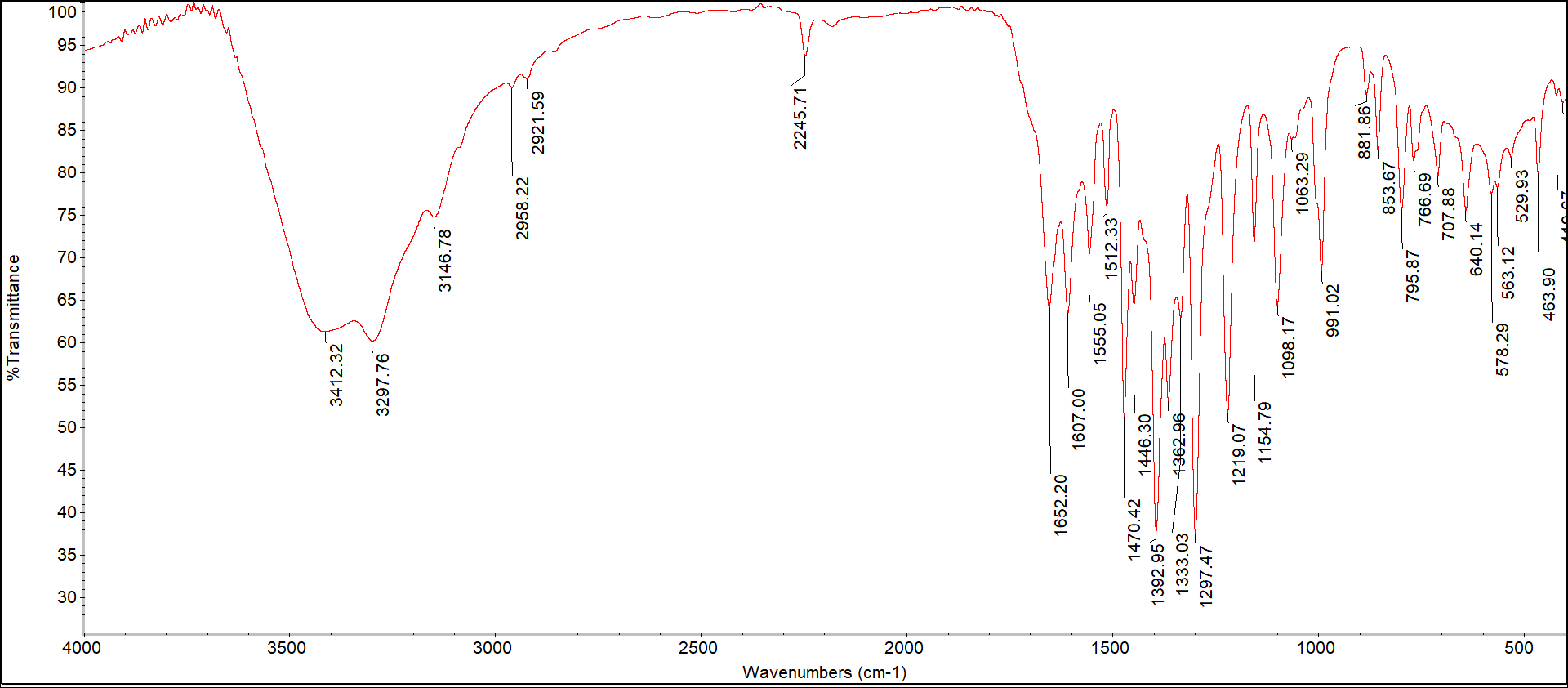


**Figure S10.** **IR** spectrum of compound **10**


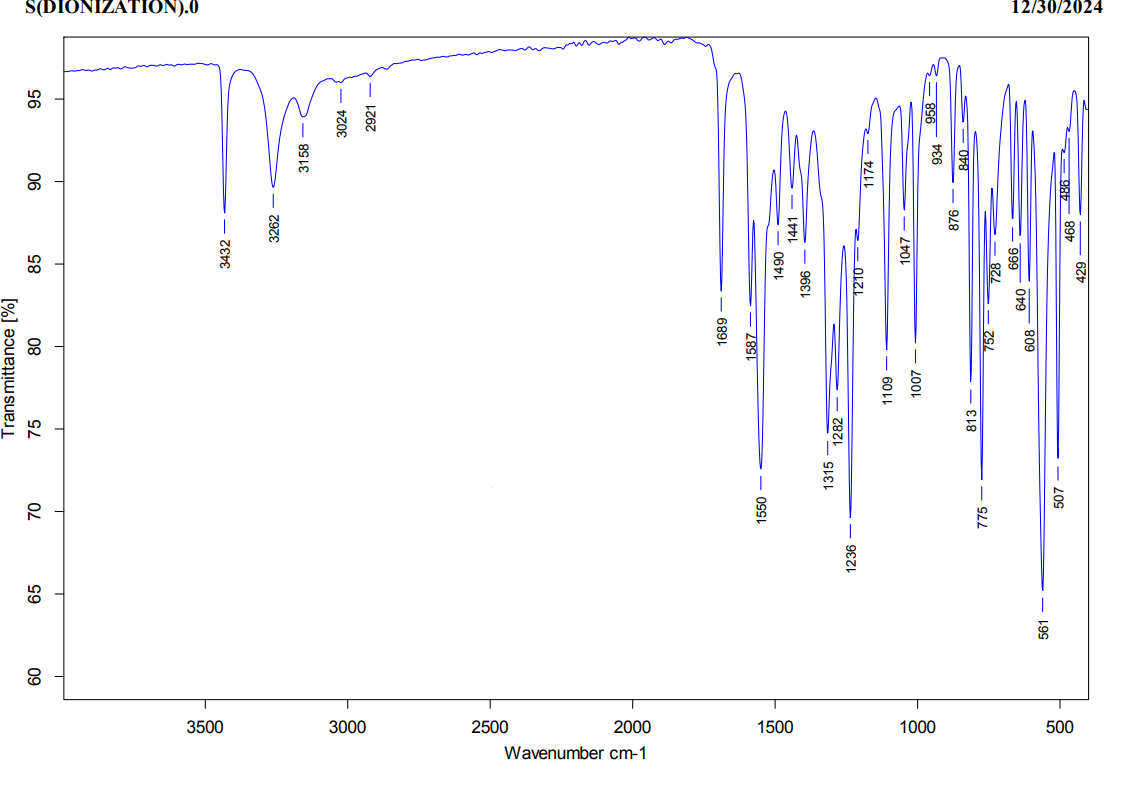


**Figure S11.** **IR** spectrum of compound **11**


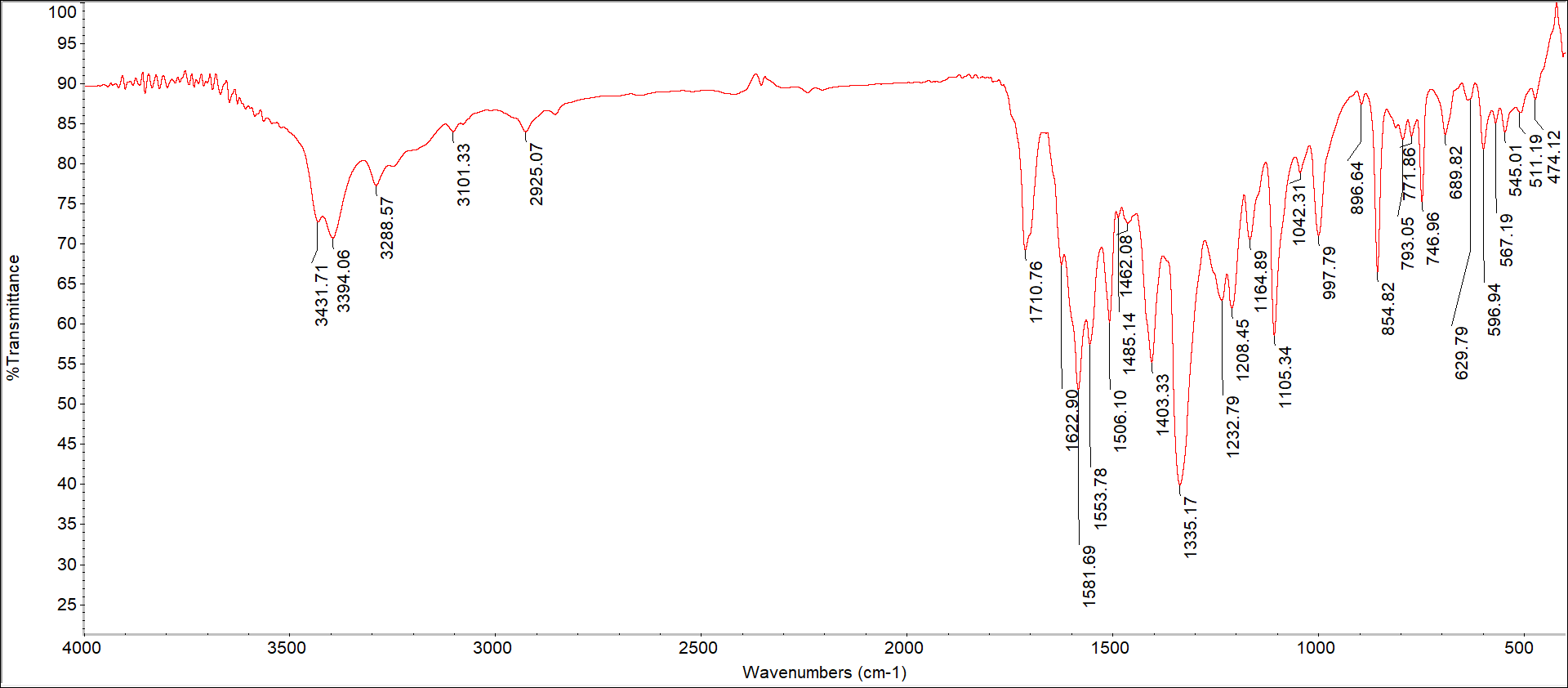


**Figure S12.** **IR** spectrum of compound **12**


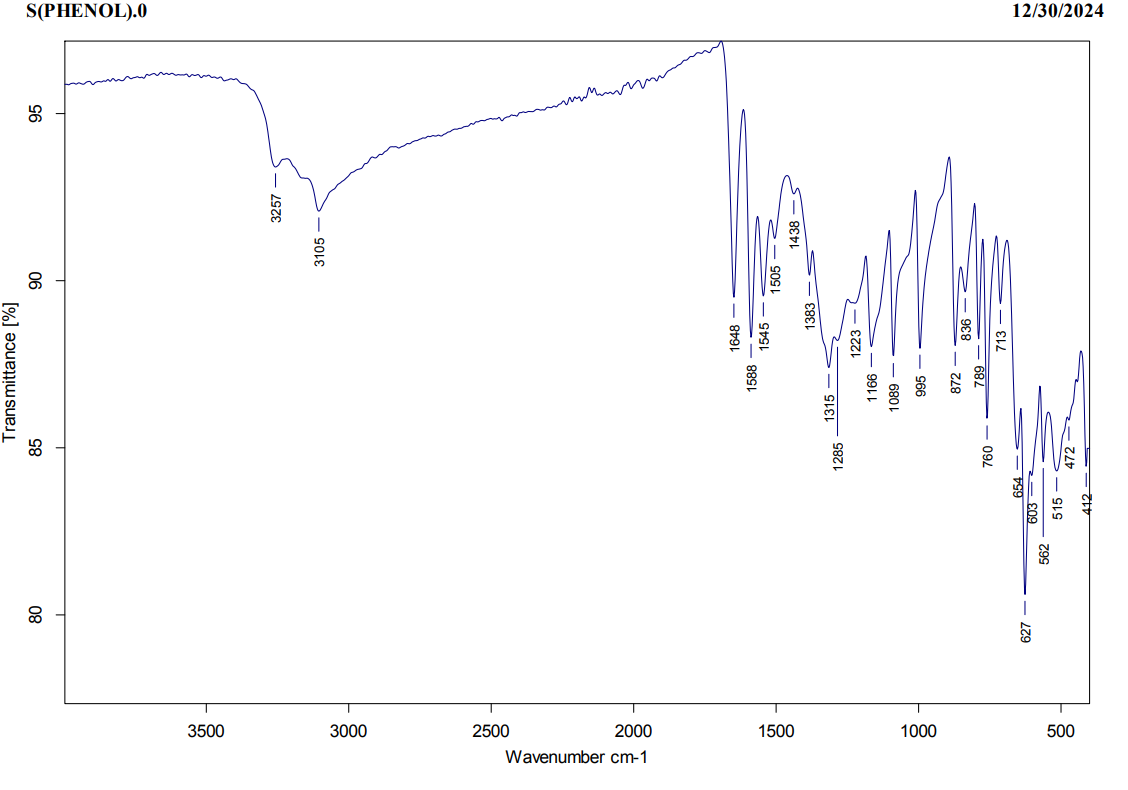


**Figure S13.** **IR** spectrum of compound **13**


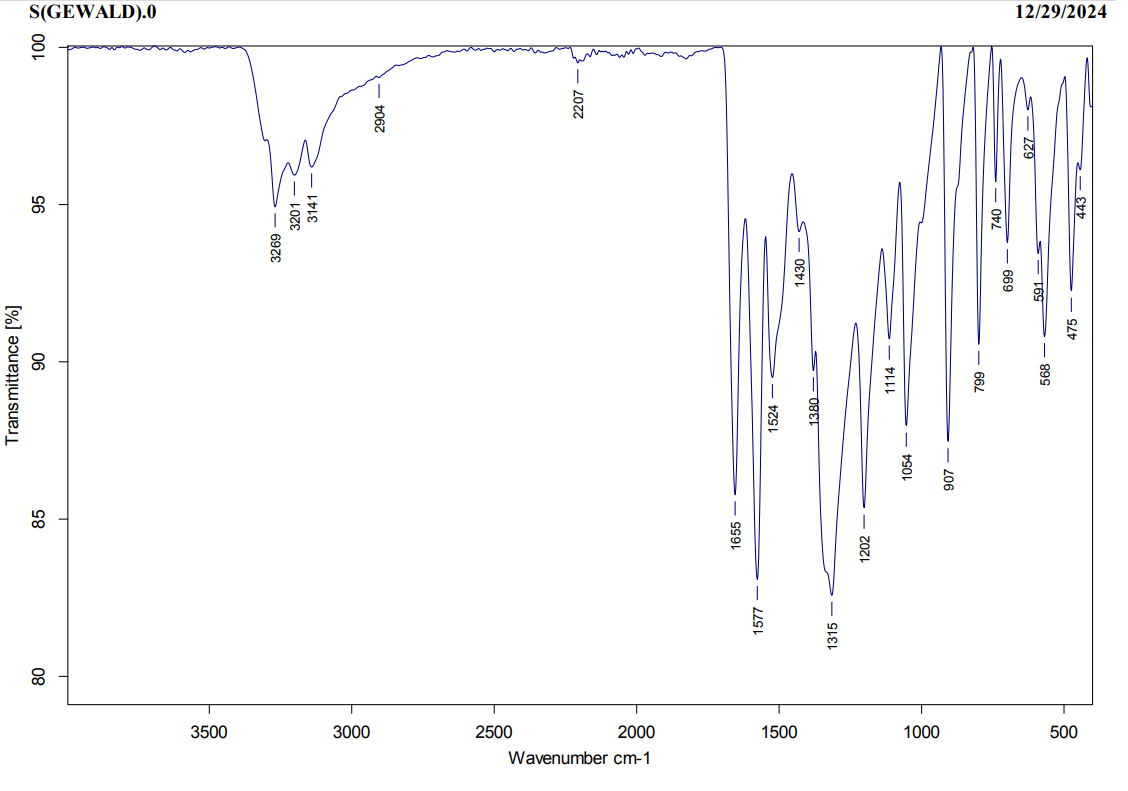


**Figure S14.** **IR** spectrum of compound **14**


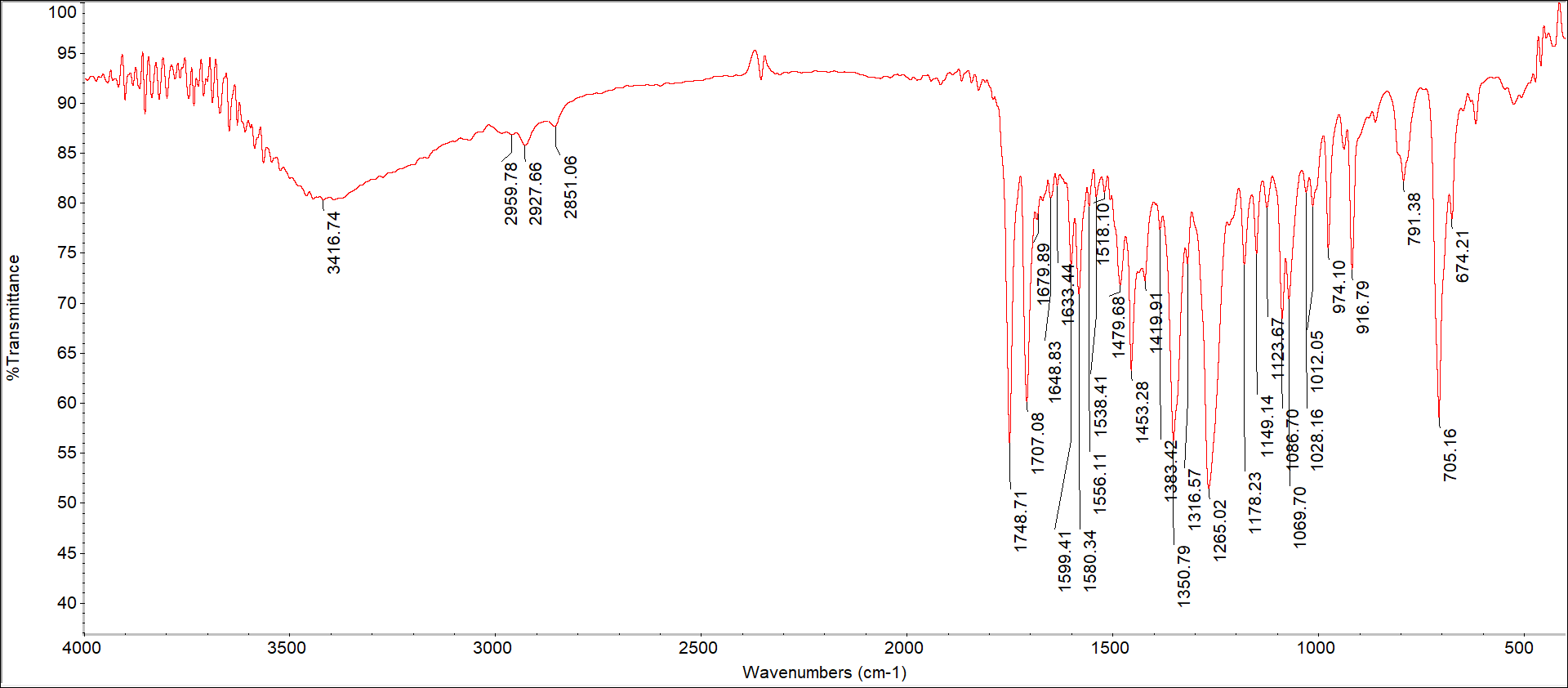


**Figure S15.** **IR** spectrum of compound **15**


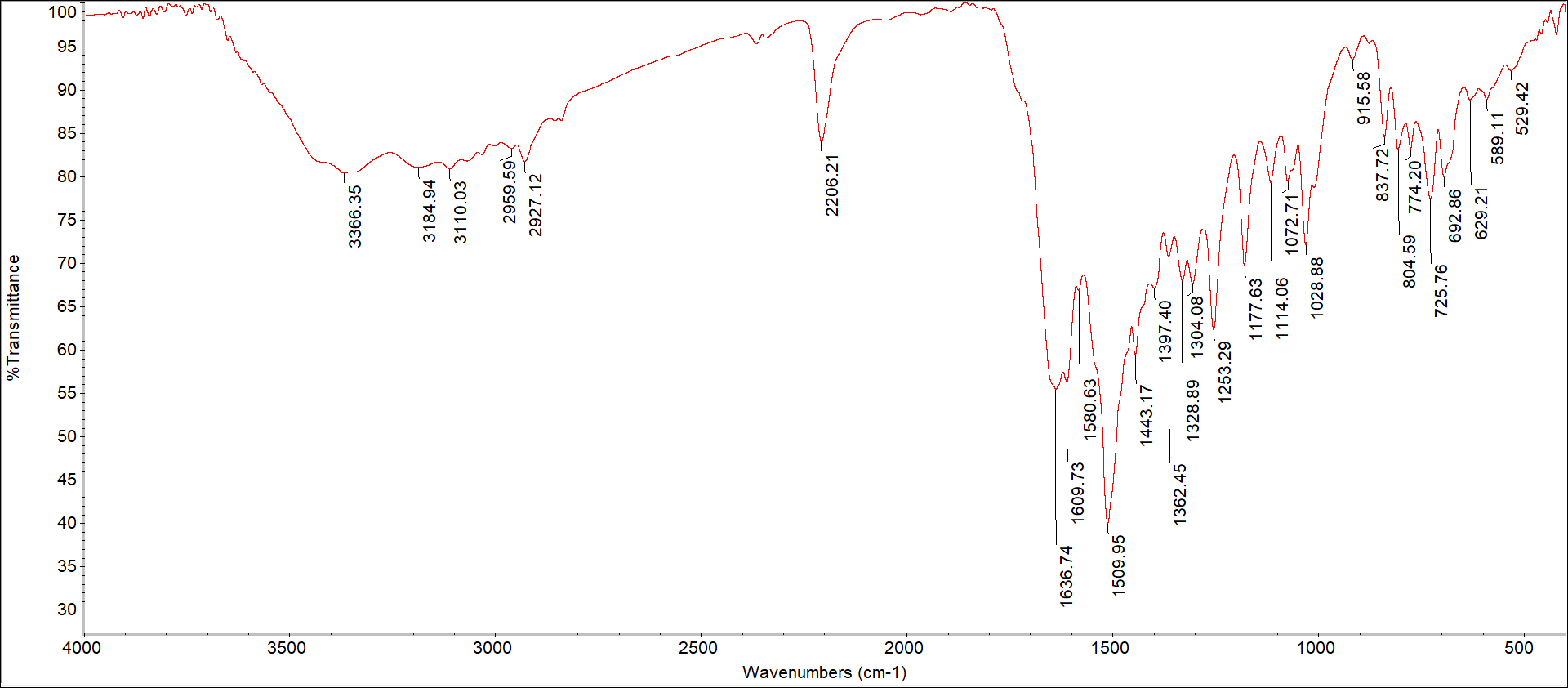


**Figure S16.** **IR** spectrum of compound **16**


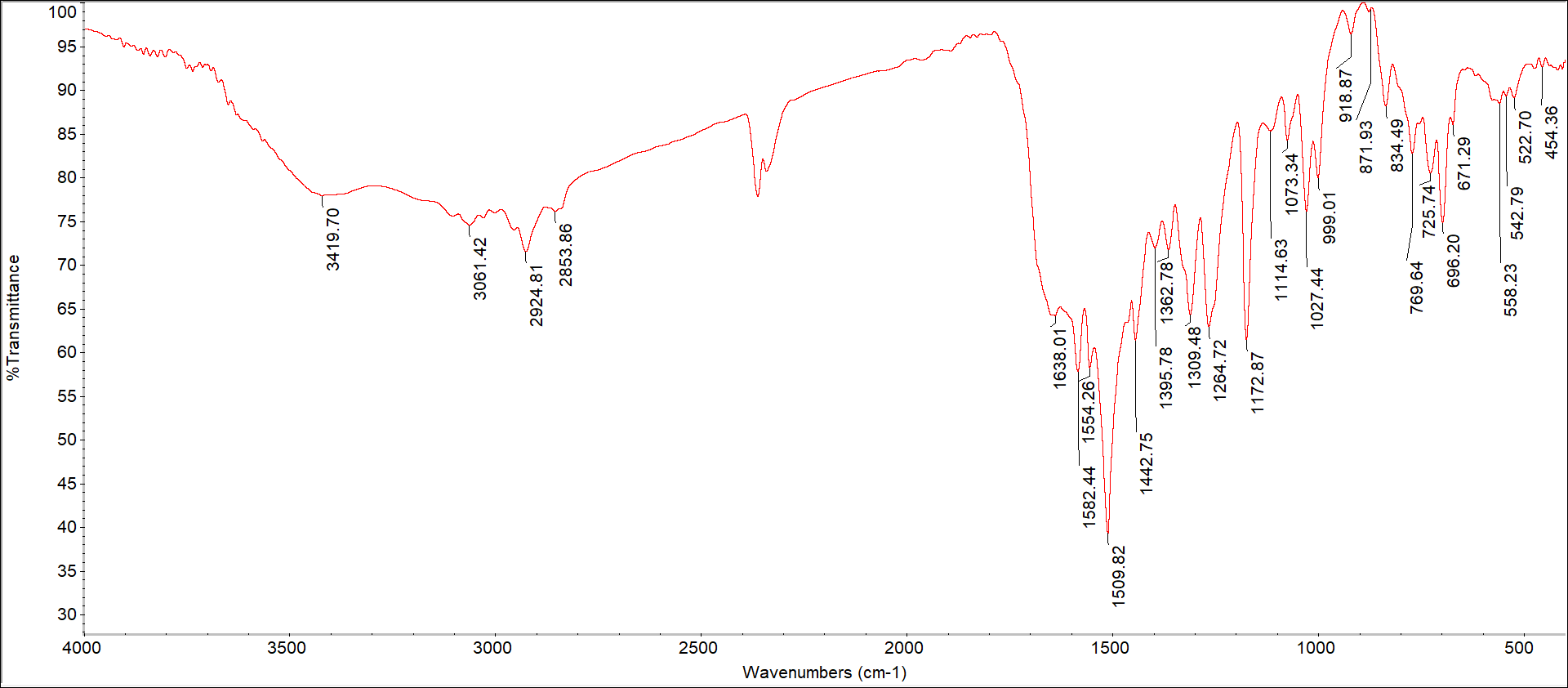


**Figure S17.** **IR** spectrum of compound **17**


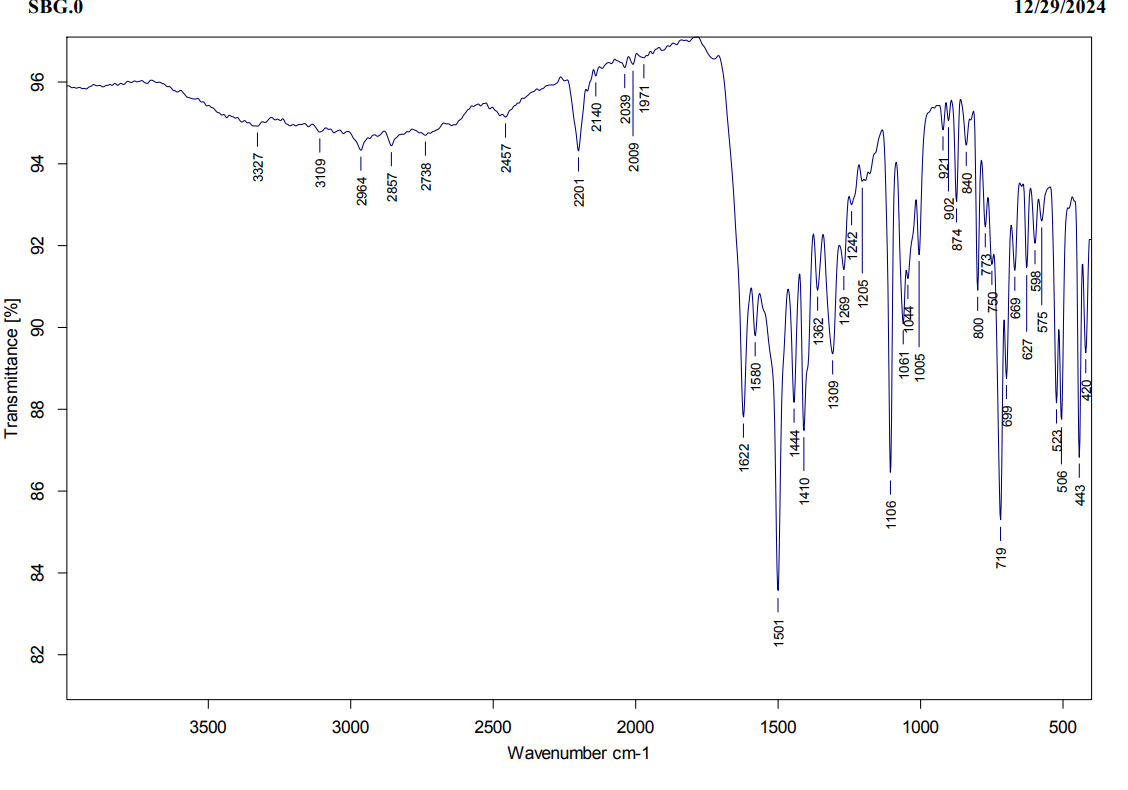


**Figure S18.** **IR** spectrum of compound **18**


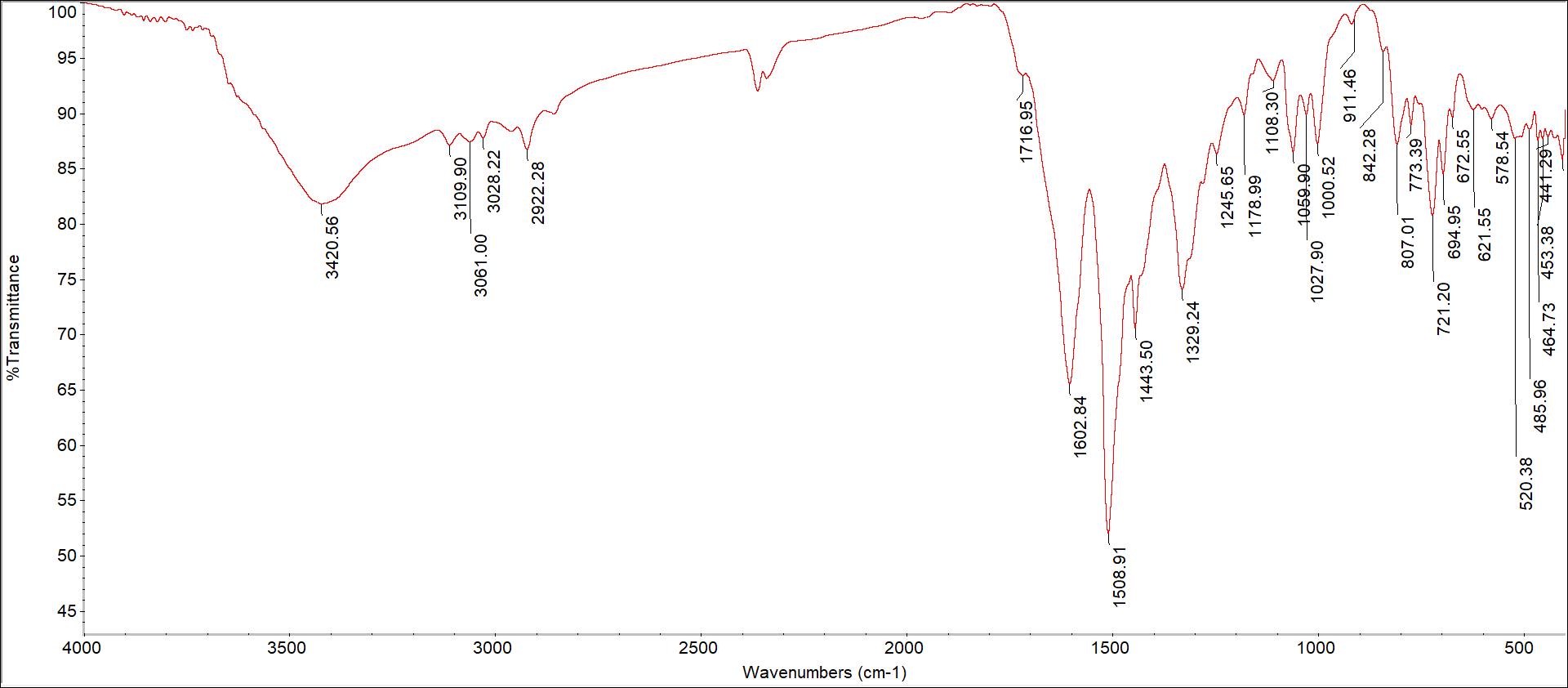


**Figure S19.** **IR** spectrum of compound **19**

**^1^H NMR spectral data of the target compounds**


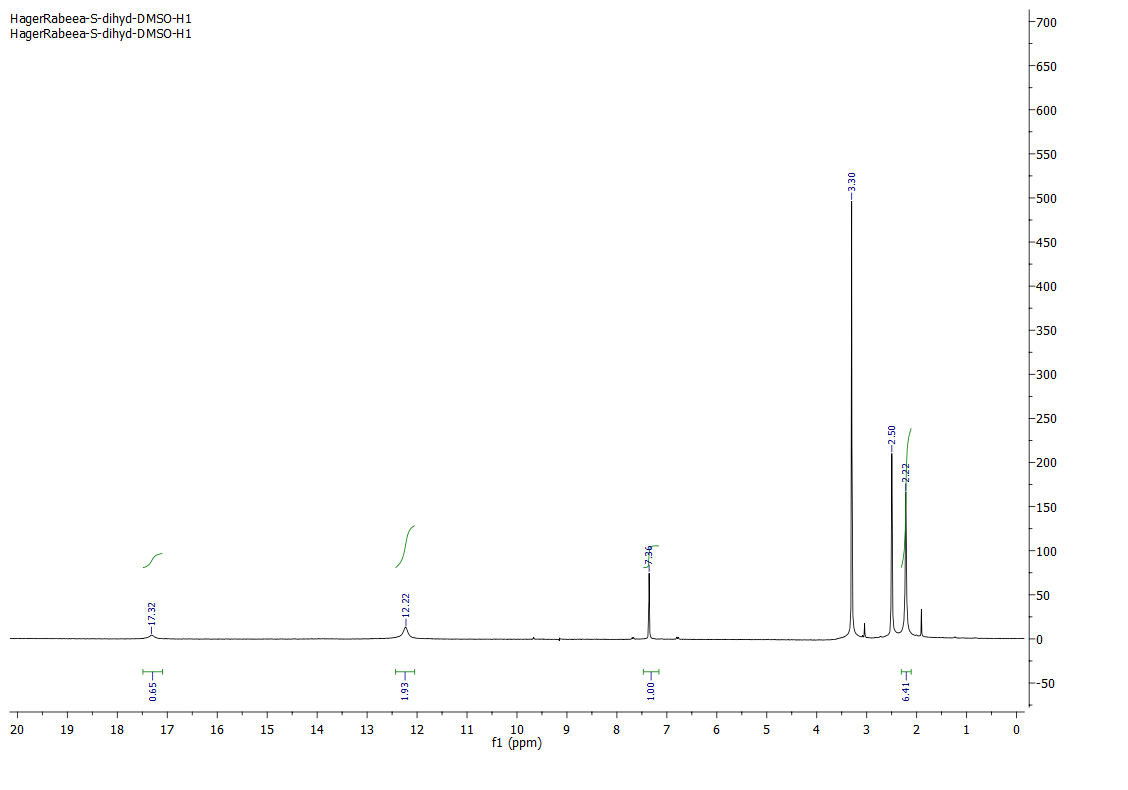


**Figure S21.** ^1^H NMR (500 MHz, DMSO-*d_6_*) spectrum of compound **2**


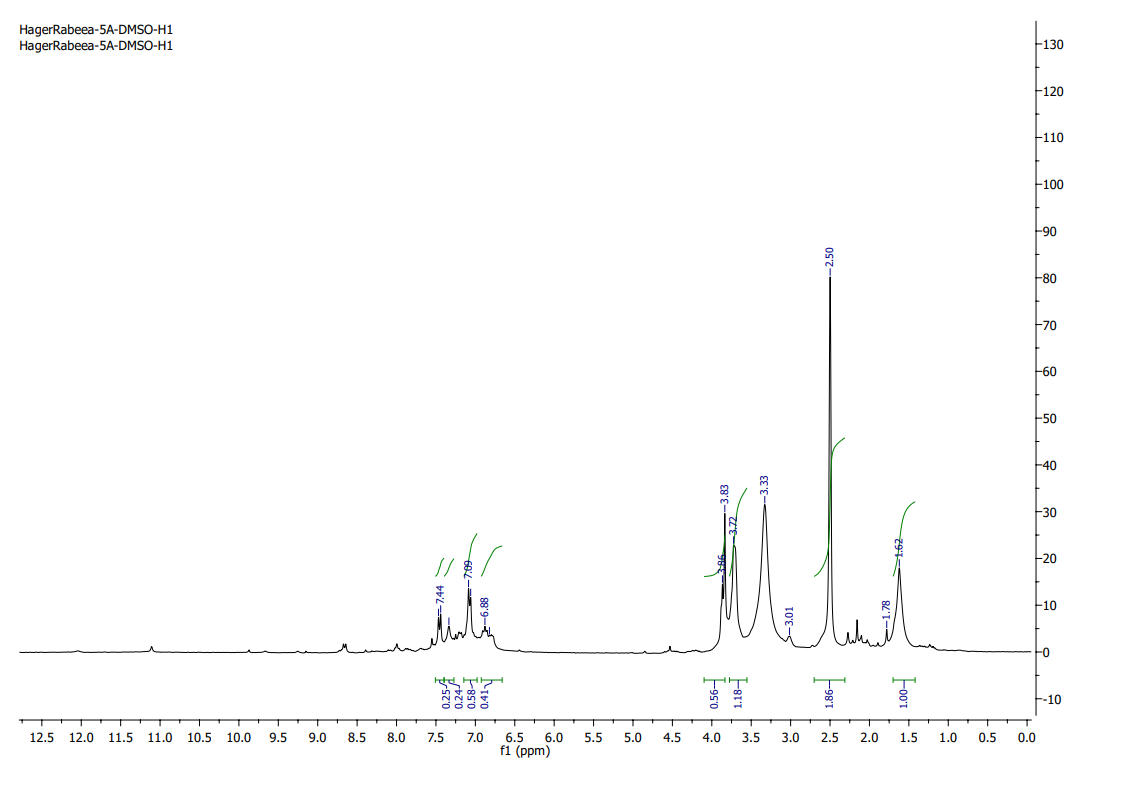


**Figure S22.** ^1^H NMR (500 MHz, DMSO-*d_6_*) spectrum of compound **4**


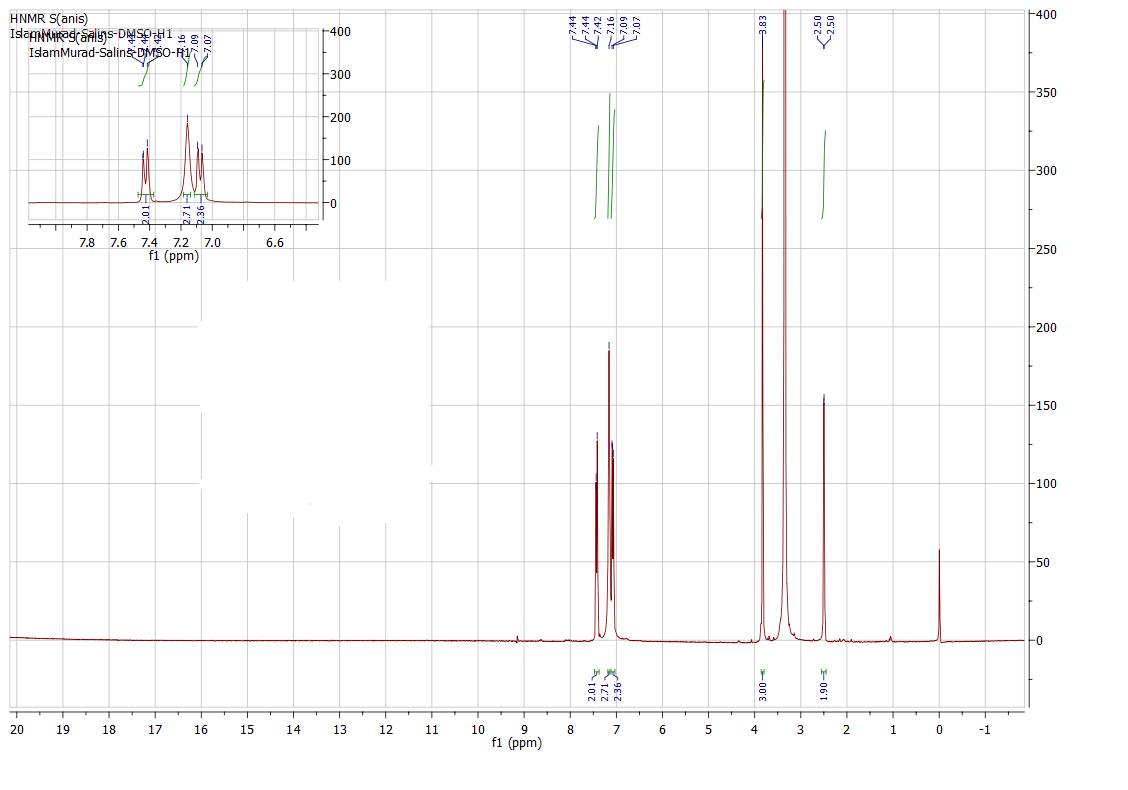


**Figure S23.** ^1^H NMR (500 MHz, DMSO-*d_6_*) spectrum of compound **5**


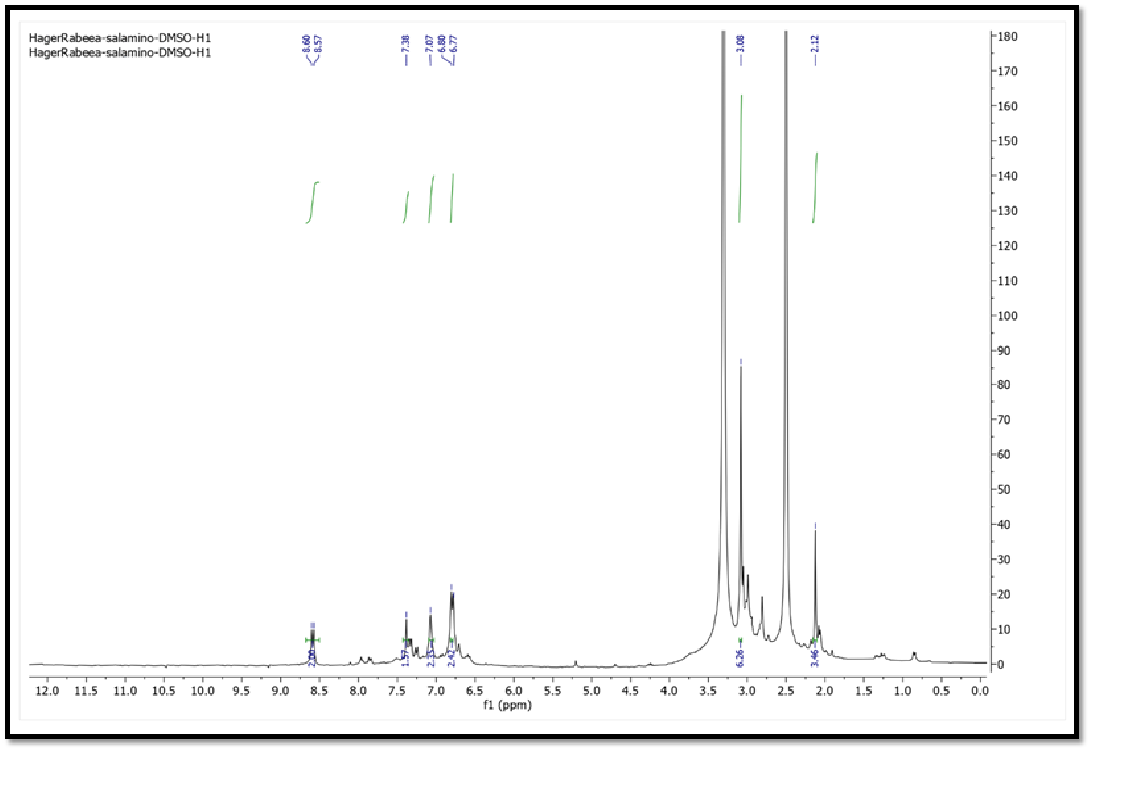


**Figure S24.** ^1^H NMR (500 MHz, DMSO-*d_6_*) spectrum of compound **6**

**
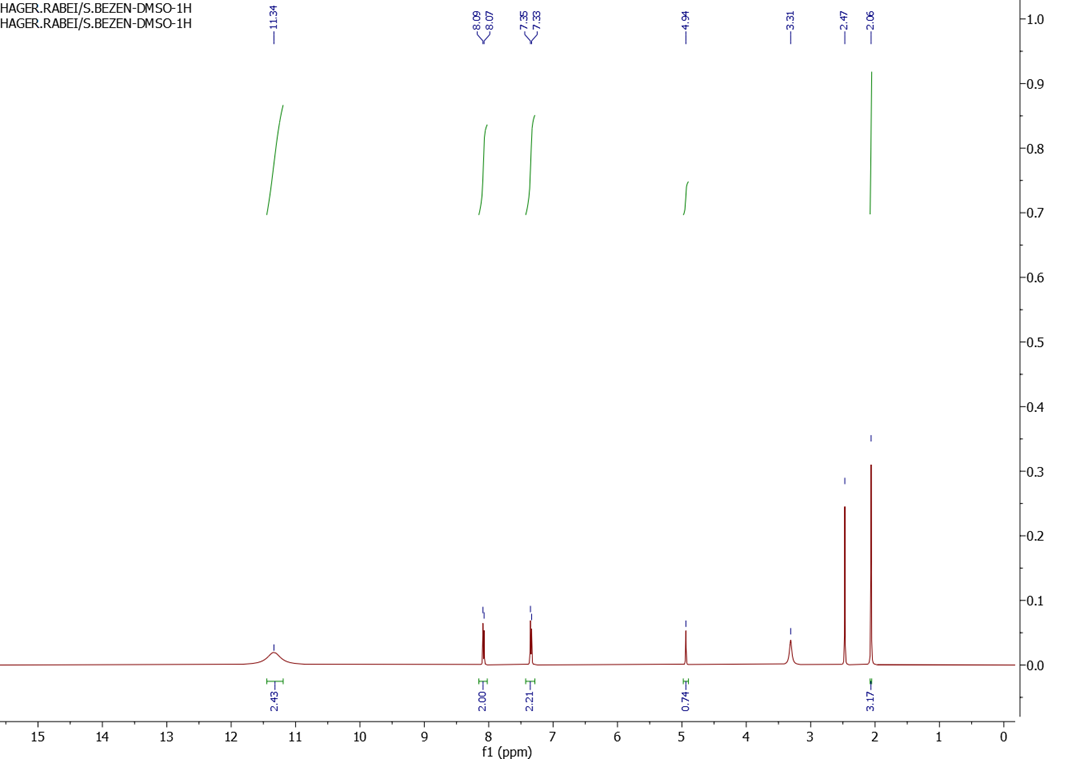
**

**Figure S25.** ^1^H NMR (500 MHz, DMSO-*d_6_*) spectrum of compound **7**


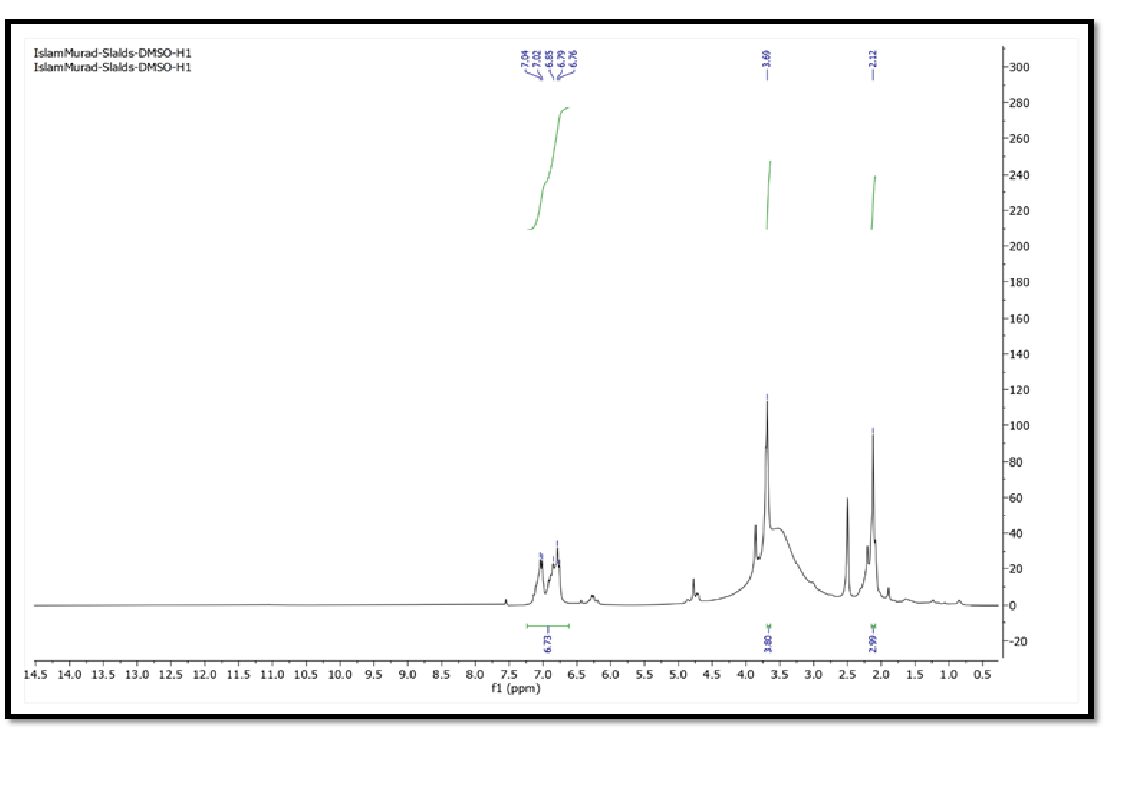


**Figure S26.** ^1^H NMR (500 MHz, DMSO-*d_6_*) spectrum of compound **8**


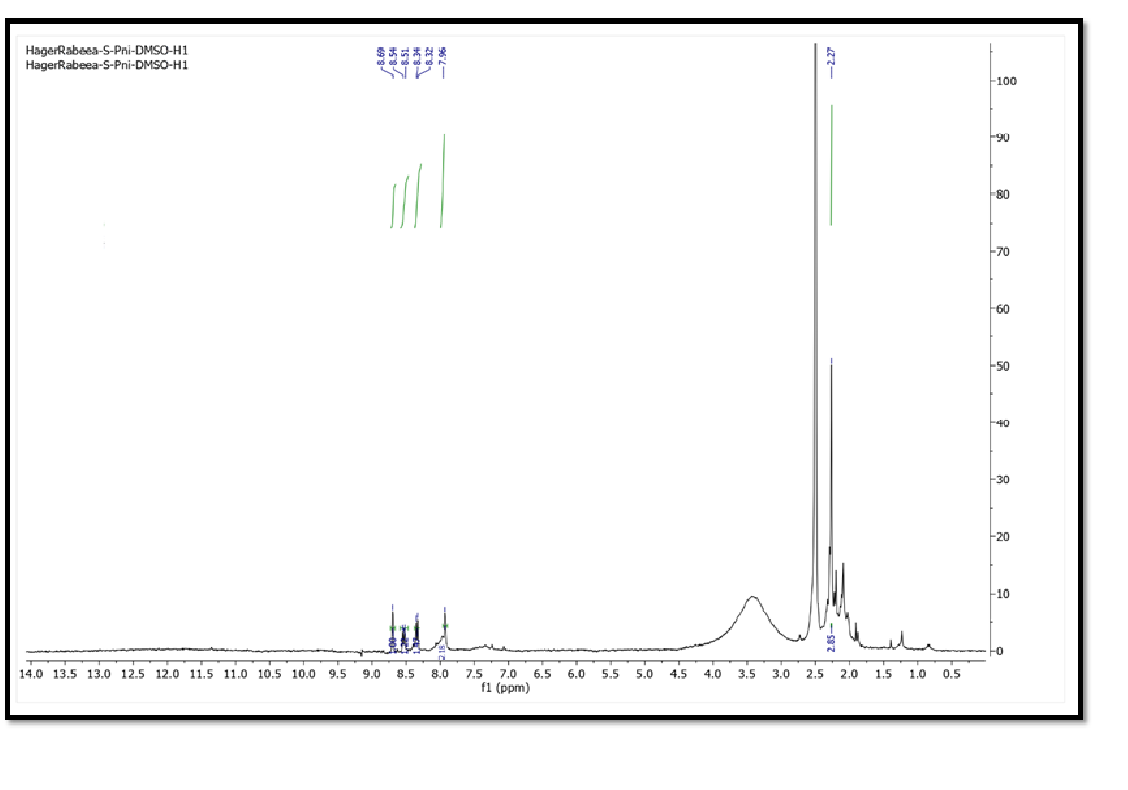


**Figure S27.** ^1^H NMR (500 MHz, DMSO-*d_6_*) spectrum of compound **9**


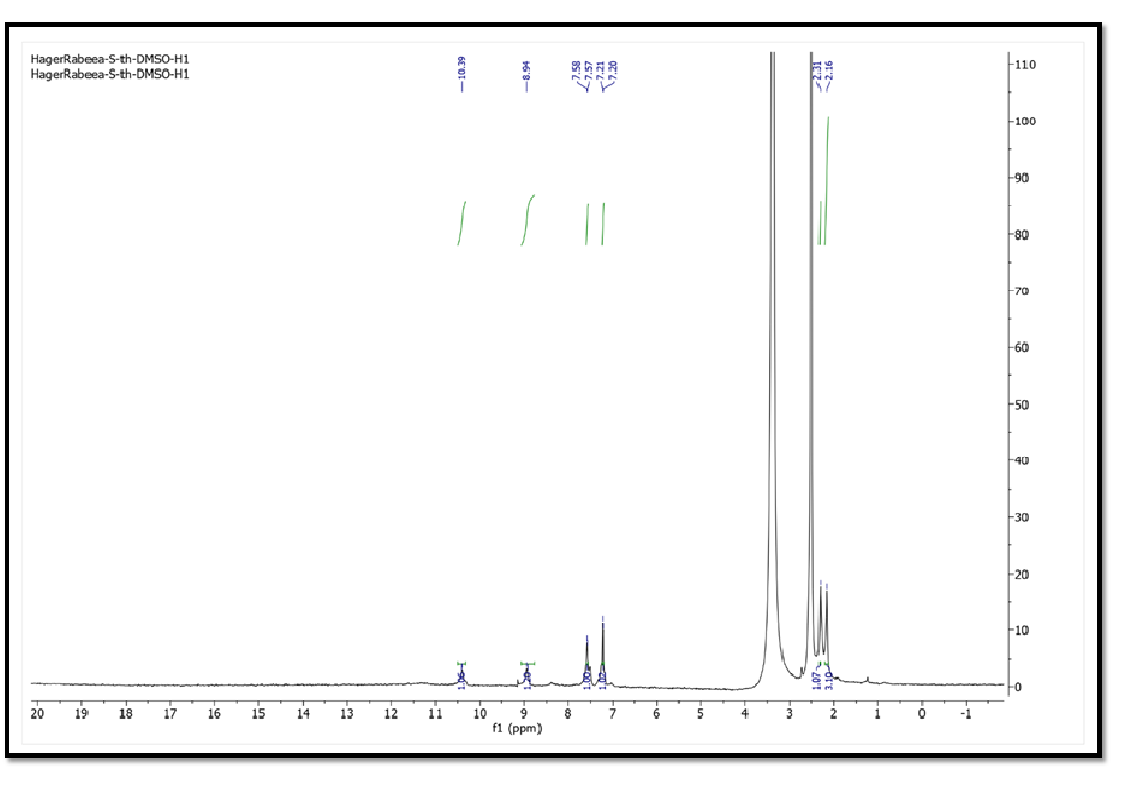


**Figure S28.** ^1^H NMR (500 MHz, DMSO-*d_6_*) spectrum of compound **10**


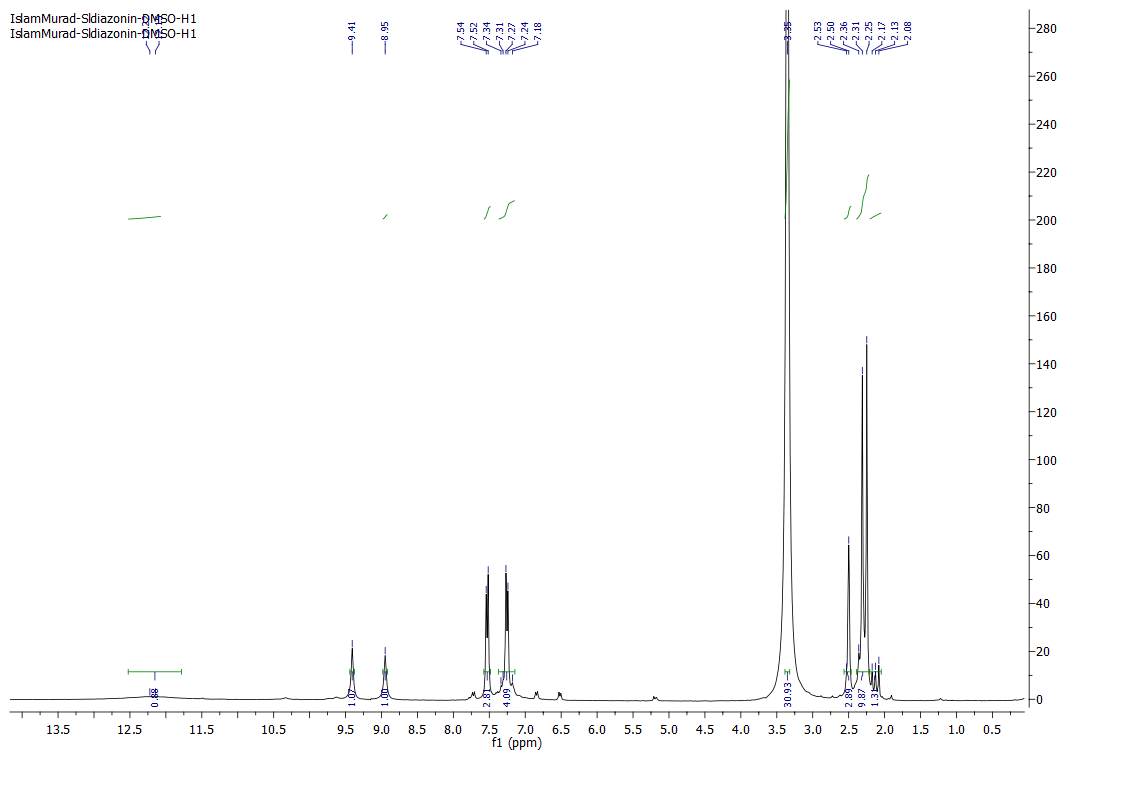


**Figure S29.** ^1^H NMR (500 MHz, DMSO-*d_6_*) spectrum of compound **11**


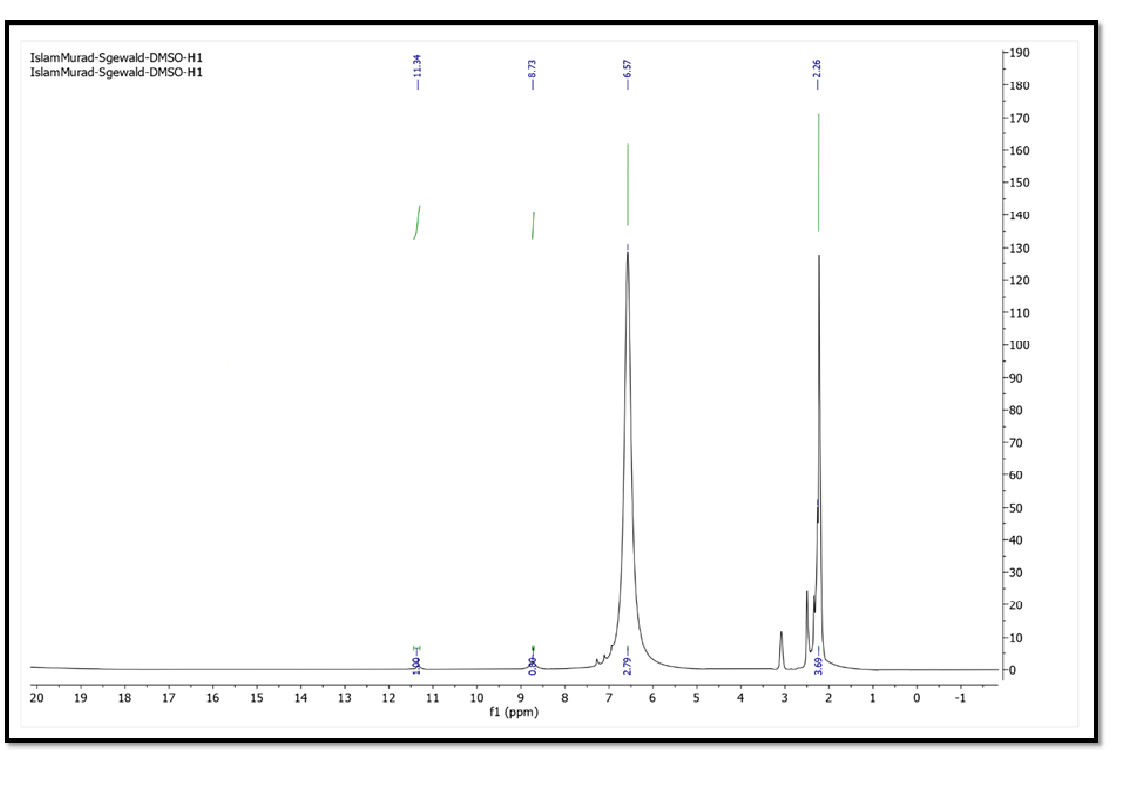


**Figure S30.** ^1^H NMR (500 MHz, DMSO-*d_6_*) spectrum of compound **14**


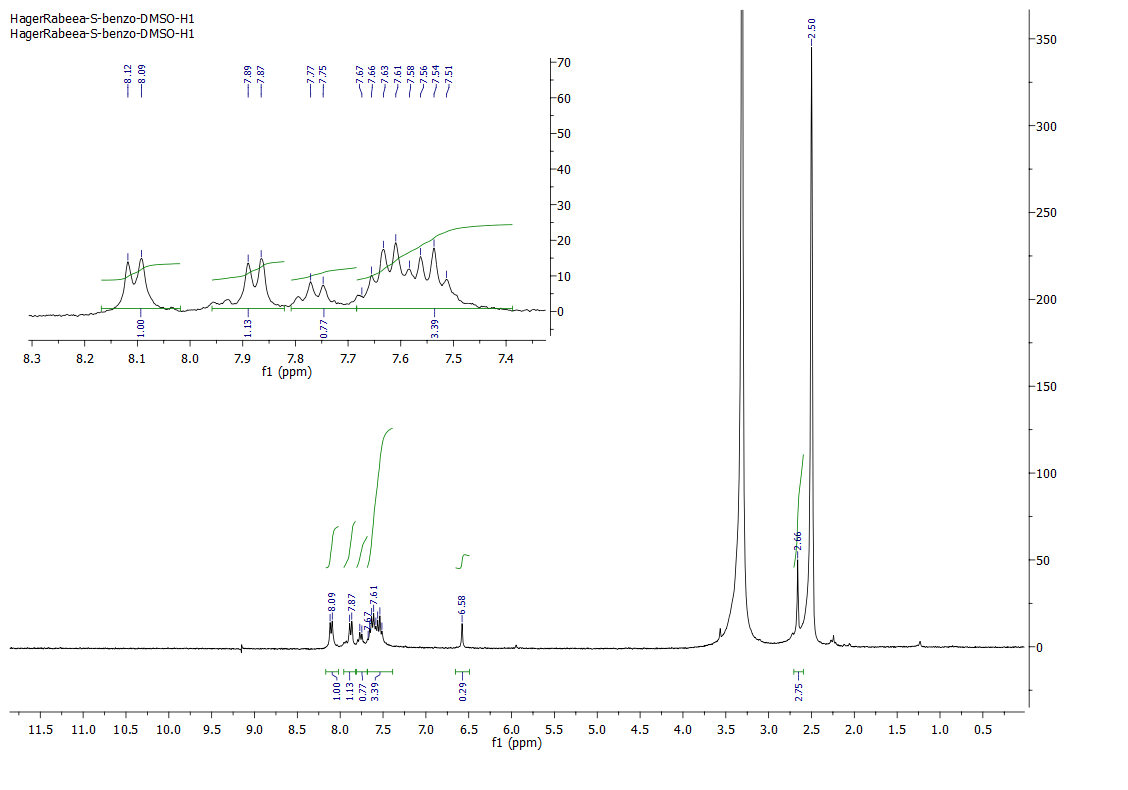


**Figure S31.** ^1^H NMR (500 MHz, DMSO-*d_6_*) spectrum of compound **15**

**
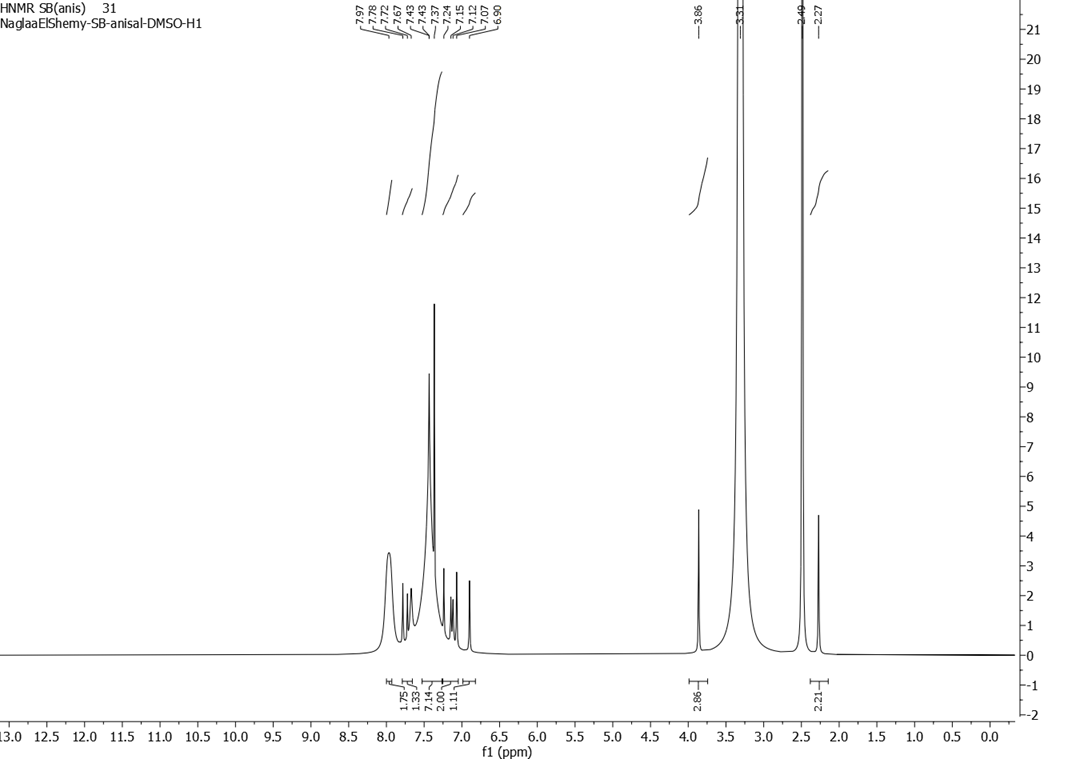
**

**Figure S32.** ^1^H NMR (500 MHz, DMSO-*d_6_*) spectrum of compound **16**

**
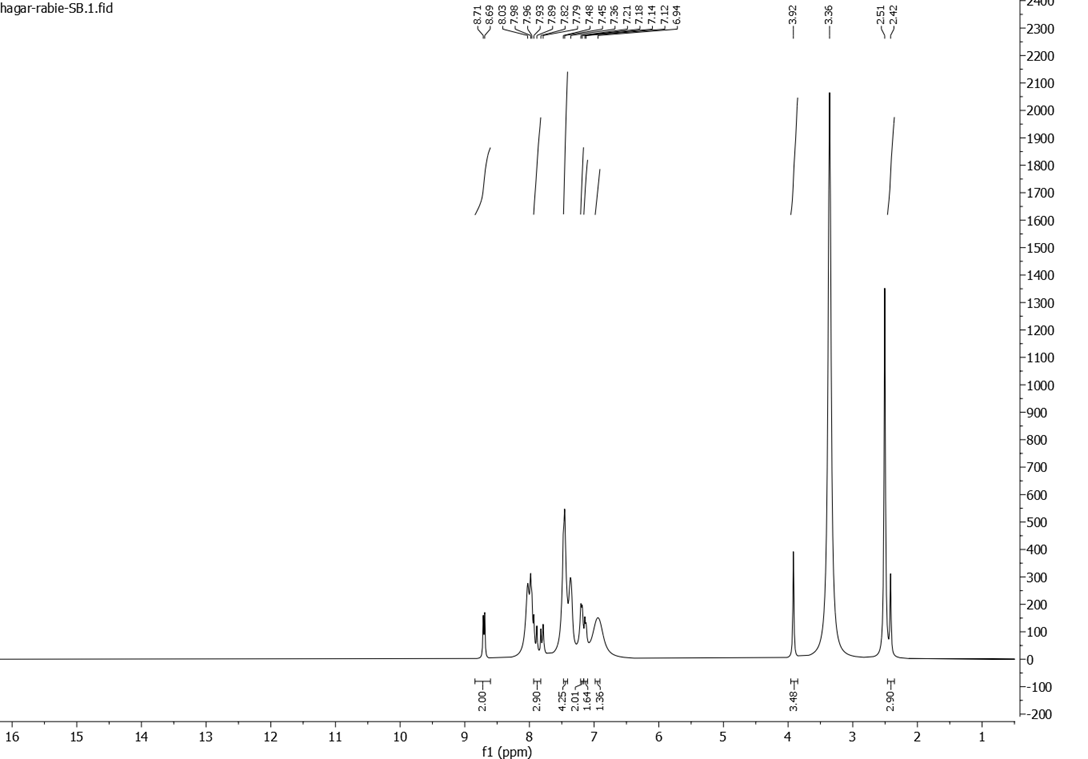
**

**Figure S33.** ^1^H NMR (500 MHz, DMSO-*d_6_*) spectrum of compound **17**

**
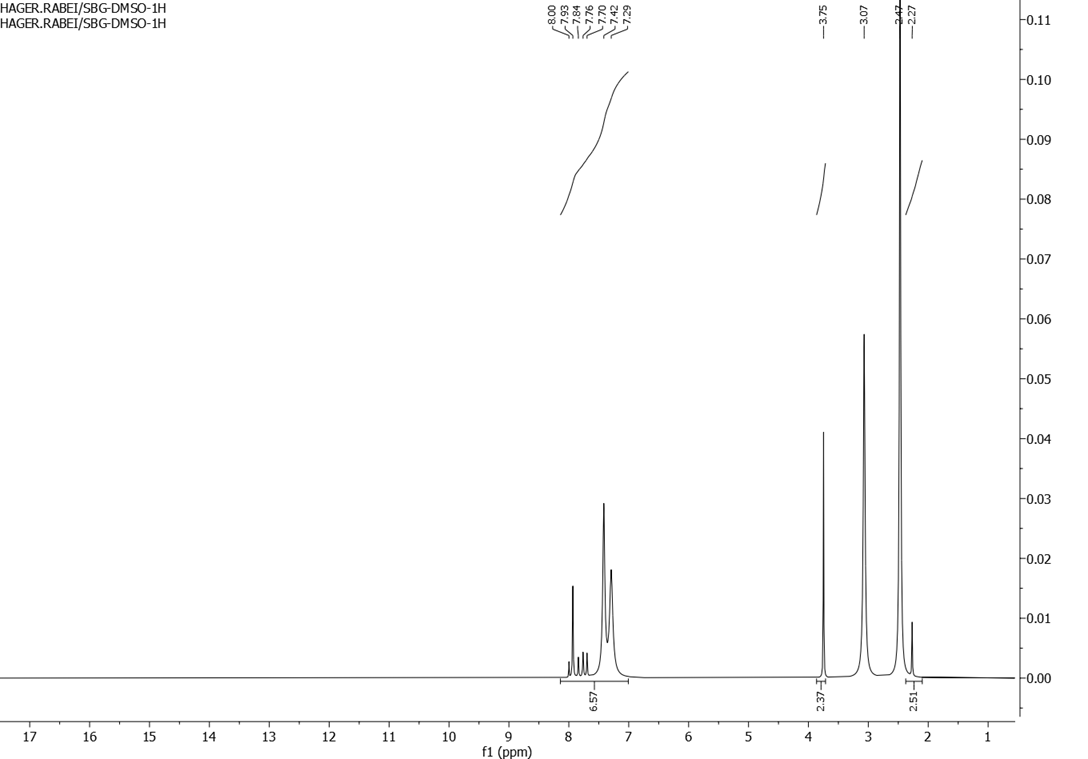
**

**Figure S34.** ^1^H NMR (500 MHz, DMSO-*d_6_*) spectrum of compound **18**

**Mass fragmentation of the target compounds**

**Figure S35.** Mass fragmentation of compound **4**

**Figure S36.** Mass fragmentation of compound **5**

**Figure S37.** Mass fragmentation of compound **6**

**Figure S38.** Mass fragmentation of compound **7**

**Figure S39.** Mass fragmentation of compound **8**

**Figure S40.** Mass fragmentation of compound **9**

**Figure S41.** Mass fragmentation of compound **10**

**Figure S42.** Mass fragmentation of compound **11**

**Figure S43.** Mass fragmentation of compound **14**

**Figure S44.** Mass fragmentation of compound **15**

**Figure S45.** Mass fragmentation of compound **16**

**Figure S46.** Mass fragmentation of compound **17**

**Figure S47.** Mass fragmentation of compound **18**


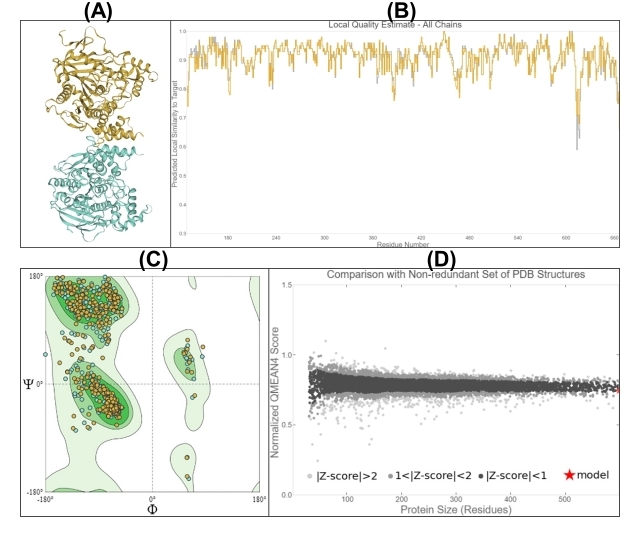


**Figure S47.** Quality estimate parameters for modeled Acetylcholine Esterase (AChE) protein. (A) Modelled AChE 3D Structure, (B) local model quality estimate, (C) Ramachandran plot, and (D) comparison with a non-redundant set of PDB structures. This figure was generated using SWISS-MODEL (online tool, URL: https://swissmodel.expasy.org).

**
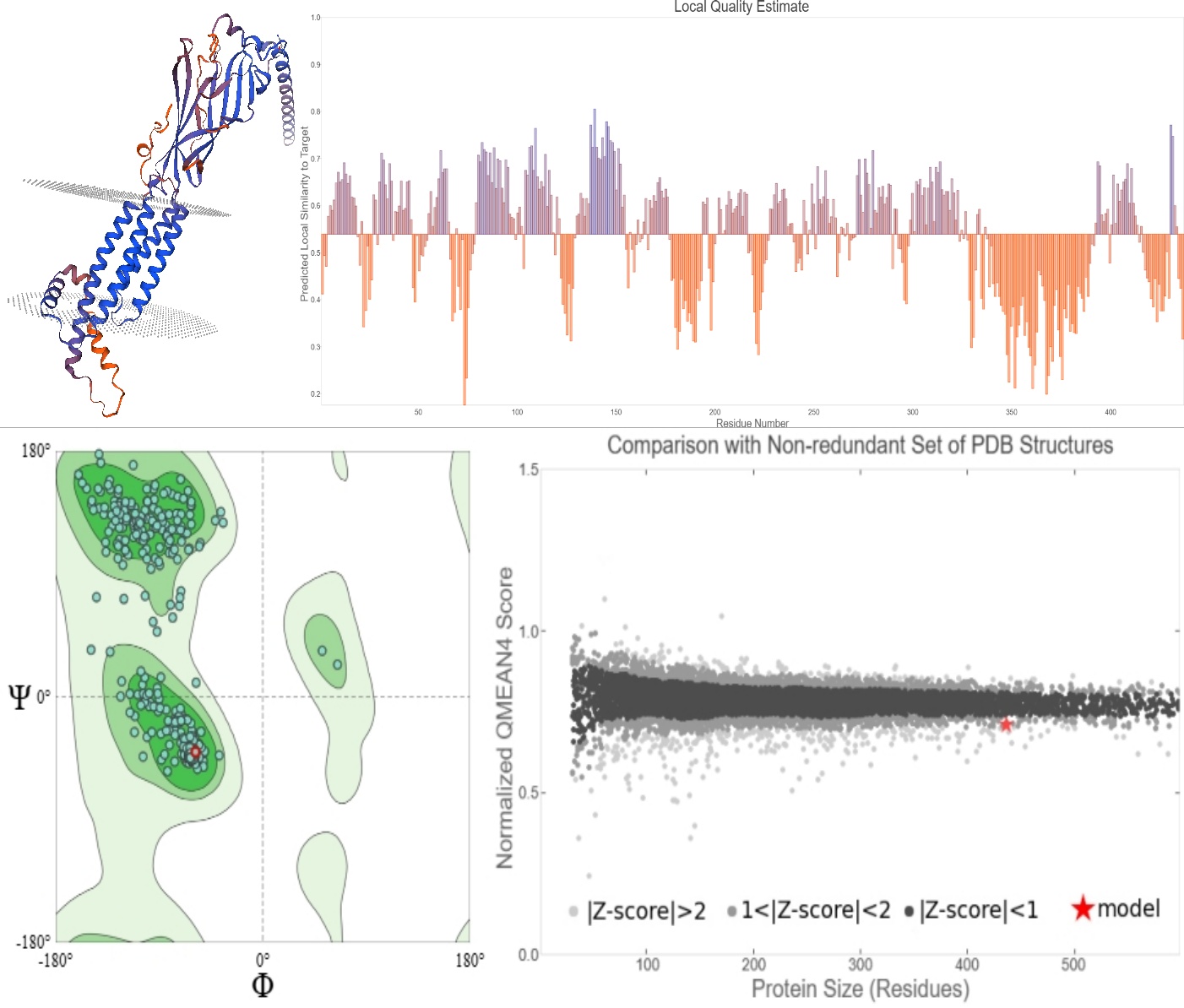
**

**Figure S48.** Quality estimate parameters for modeled nicotinic acetylcholine receptor. (A) Modelled nAChR 3D Structure, (B) local model quality estimate, (C) Ramachandran plot, and (D) comparison with a non-redundant set of PDB structures. This figure was generated using SWISS-MODEL (online tool, URL: https://swissmodel.expasy.org).

**
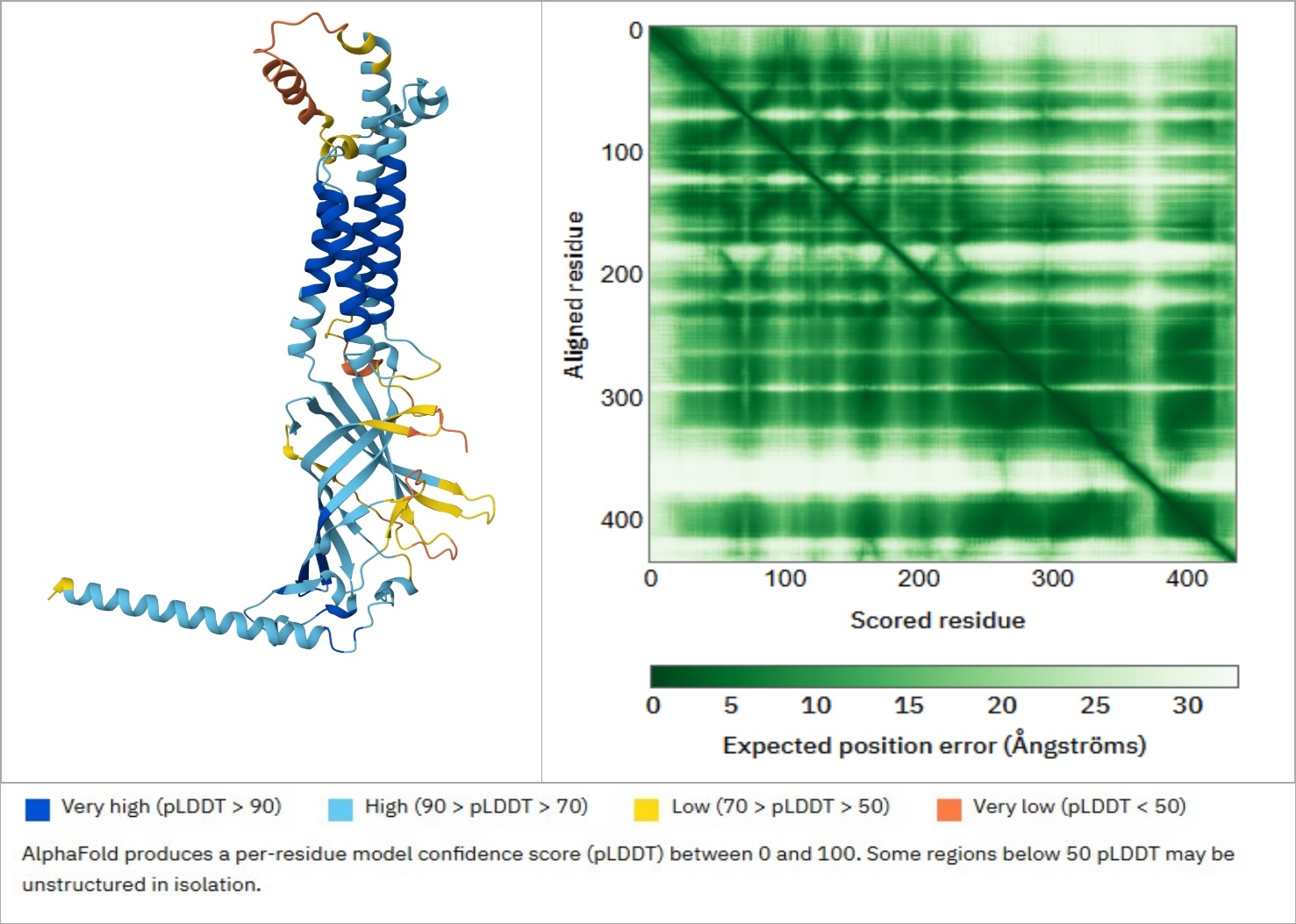
**

**Figure S49.** AlphaFold Model of the Nicotinic Acetylcholine Receptor (nAChR) from *Culex quinquefasciatus.* This figure presents the predicted three-dimensional structure and confidence metrics of the *Culex quinquefasciatus* nicotinic acetylcholine receptor (nAChR) obtained using AlphaFold. **(Left Panel):** The predicted structural model is color-coded based on per-residue confidence scores (pLDDT). Regions with very high confidence (pLDDT > 90) are shown in dark blue, high-confidence regions (70 < pLDDT < 90) in light blue, lower-confidence regions (50 < pLDDT < 70) in yellow, and regions with very low confidence (pLDDT < 50) in orange. Regions with lower confidence may be flexible or disordered in isolation. **(Right Panel):** The predicted alignment error (PAE) matrix indicates the expected positional error in Ångströms for each residue pair. The diagonal represents intra-domain confidence, while off-diagonal regions suggest inter-domain flexibility or uncertainty. Dark green indicates low expected error (higher confidence), whereas lighter shades denote higher uncertainty. This model provides structural insights into the *Culex quinquefasciatus* nAChR, which plays a critical role in neurotransmission and is a potential target for insecticides.

| **Table S1.** 2D interaction profiles of the 19 synthesized compounds targeting acetylcholinesterase (AChE) in *Culex pipiens*, compared with the conventional AChE inhibitor chlorpyrifos | |
| --- | --- |
| **1** | **2** |
| ** 3** | **4** |
| ** 5** | **6** |
| ** 7** | **8 ** |
| **9** | **10** |
| **11** | **12** |
| **13** | **14** |
| **15** | **16** |
| **17** | **18** |
| **19 ** | **Chlorpyrifos** |

| Table S2. Molecular docking interaction profiles of 19 synthesized compounds with acetylcholinesterase (AChE) in *Culex pipiens*, showing ligand–receptor interacting atoms, interacting residues, interaction types, hydrogen bond distances, and binding energies (E, kcal/mol). Data are compared with the conventional AChE inhibitor chlorpyrifos | | | | | | |
| --- | --- | --- | --- | --- | --- | --- |
| Ligand | **Ligand interacting atoms** | **Receptor Interacting atoms** | **Receptor Interacting Residues** | **Interaction Bond Type** | **Distance** | **E (kcal/mol)** |
| 1 | N 21 | OD1 | ASN 213 (B) | H-donor | 3.04 | -0.8 |
|  | O 11 | OH | TYR 249 (B) | H-acceptor | 3 | -0.8 |
|  | S 20 | CA | VAL 199 (B) | H-acceptor | 4.43 | -0.9 |
|  | S 20 | N | ASP 200 (B) | H-acceptor | 4.16 | -2.5 |
|  | S 20 | CA | ASN 213 (B) | H-acceptor | 3.86 | -0.8 |
|  | S 20 | CD | PRO 214 (B) | H-acceptor | 3.85 | -0.5 |
|  | S 20 | CB | SER 250 (B) | H-acceptor | 3.68 | -0.9 |
| 2 | S 14 | CA | GLY 251 (B) | H-acceptor | 3.54 | -0.7 |
| 3 | O 12 | OH | TYR 249 (B) | H-acceptor | 2.9 | -1.5 |
|  | 6-ring | 5-ring | TRP 212 (B) | pi-pi | 4.34 |  |
|  | 6-ring | 6-ring | TRP 212 (B) | pi-pi | 4.01 |  |
| 4 | N 17 | O | VAL 199 (B) | H-donor | 2.88 | -0.9 |
|  | 5-ring | CE1 | TYR 249 (B) | pi-H | 3.77 | -0.8 |
| 5 | S 19 | CA | VAL 199 (B) | H-acceptor | 4.31 | -0.5 |
|  | S 19 | N | ASP 200 (B) | H-acceptor | 4.08 | -0.8 |
|  | S 19 | CA | ASN 213 (B) | H-acceptor | 4.16 | -1.2 |
|  | S 19 | CD | PRO 214 (B) | H-acceptor | 4.03 | -0.7 |
|  | S 19 | CB | SER 250 (B) | H-acceptor | 3.54 | -0.5 |
|  | 6-ring | 6-ring | TYR 460 (B) | pi-pi | 4.22 |  |
| 6 | N 20 | O | ILE 198 (B) | H-donor | 2.84 | -1.6 |
|  | S 19 | CA | VAL 199 (B) | H-acceptor | 4.2 | -0.9 |
|  | S 19 | CA | ASN 213 (B) | H-acceptor | 3.9 | -1 |
|  | S 19 | CD | PRO 214 (B) | H-acceptor | 3.45 | -1.1 |
|  | 6-ring | CE1 | TYR 249 (B) | pi-H | 3.91 | -0.9 |
| 7 | N 20 | OD1 | ASN 213 (B) | H-donor | 2.71 | -1.2 |
|  | S 19 | CA | VAL 199 (B) | H-acceptor | 4.2 | -0.9 |
|  | S 19 | N | ASP 200 (B) | H-acceptor | 4.1 | -1.7 |
|  | S 19 | CA | ASN 213 (B) | H-acceptor | 3.81 | -0.8 |
|  | S 19 | CD | PRO 214 (B) | H-acceptor | 3.57 | -0.6 |
|  | S 19 | CB | SER 250 (B) | H-acceptor | 3.8 | -0.7 |
|  | N 16 | 6-ring | TYR 460 (B) | H-pi | 4.54 | -0.6 |
|  | 6-ring | CE1 | TYR 249 (B) | pi-H | 3.9 | -0.7 |
| 8 | N 13 | O | ILE 198 (B) | H-donor | 3.1 | -0.8 |
|  | S 12 | CA | VAL 199 (B) | H-acceptor | 4.2 | -1 |
|  | S 12 | N | ASP 200 (B) | H-acceptor | 4.17 | -0.6 |
|  | S 12 | CA | ASN 213 (B) | H-acceptor | 3.9 | -1.1 |
|  | S 12 | CD | PRO 214 (B) | H-acceptor | 3.56 | -1.1 |
| 9 | N 13 | O | ILE 198 (B) | H-donor | 3.22 | -1.7 |
|  | O 10 | OH | TYR 249 (B) | H-acceptor | 3.06 | -1.3 |
|  | S 12 | CE1 | TYR 249 (B) | H-acceptor | 3.88 | -0.5 |
|  | O 30 | OH | TYR 258 (B) | H-acceptor | 3.23 | -0.7 |
| 10 | N 13 | O | ILE 198 (B) | H-donor | 3 | -3.1 |
|  | O 10 | OH | TYR 249 (B) | H-acceptor | 3.36 | -0.5 |
|  | S 12 | CE1 | TYR 249 (B) | H-acceptor | 3.96 | -0.5 |
| 11 | S 12 | CA | GLY 568 (B) | H-acceptor | 3.91 | -1.2 |
|  | 5-ring | 5-ring | TRP 212 (B) | pi-pi | 4.24 |  |
| 12 | N 13 | O | ILE 198 (B) | H-donor | 3.34 | -1.2 |
|  | O 31 | OH | TYR 258 (B) | H-acceptor | 3.15 | -1 |
|  | C 25 | 5-ring | TRP 212 (B) | H-pi | 3.61 | -0.6 |
| 13 | N 13 | O | ILE 198 (B) | H-donor | 2.87 | -1.3 |
|  | S 12 | CA | VAL 199 (B) | H-acceptor | 4.15 | -1 |
|  | S 12 | N | ASP 200 (B) | H-acceptor | 4.11 | -0.5 |
|  | S 12 | CA | ASN 213 (B) | H-acceptor | 3.98 | -1.1 |
|  | S 12 | CD | PRO 214 (B) | H-acceptor | 3.65 | -1.1 |
|  | 5-ring | CE1 | TYR 249 (B) | pi-H | 4.19 | -0.6 |
|  | 6-ring | 5-ring | TRP 212 (B) | pi-pi | 3.96 |  |
|  | 6-ring | 6-ring | TRP 212 (B) | pi-pi | 3.82 |  |
| 14 | S 3 | OG | SER 250 (B) | H-donor | 3.18 | -0.7 |
|  | N 21 | OE1 | GLU 326 (B) | H-donor | 3.11 | -2.8 |
|  | S 20 | CD2 | HIS 567 (B) | H-acceptor | 3.71 | -1.2 |
|  | N 18 | OE1 | GLU 326 (B) | Ionic | 3.91 | -0.7 |
|  | N 21 | OE1 | GLU 326 (B) | Ionic | 3.11 | -3.8 |
|  | 6-ring | 5-ring | TRP 212 (B) | pi-pi | 4.14 |  |
|  | 6-ring | 6-ring | TRP 212 (B) | pi-pi | 4.11 |  |
| 15 | N 13 | O | ILE 198 (B) | H-donor | 3.13 | -1.7 |
|  | S 12 | CA | ASN 213 (B) | H-acceptor | 3.75 | -1.4 |
|  | S 12 | CD | PRO 214 (B) | H-acceptor | 4.09 | -0.7 |
|  | 5-ring | CE1 | TYR 249 (B) | pi-H | 4.03 | -0.5 |
|  | 6-ring | 5-ring | TRP 212 (B) | pi-pi | 3.98 |  |
|  | 6-ring | 6-ring | TRP 212 (B) | pi-pi | 4.12 |  |
| 16 | N 50 | N | CYS 414 (B) | H-acceptor | 3.67 | -1.2 |
| 17 | O 16 | OH | TYR 249 (B) | H-acceptor | 2.88 | -0.8 |
|  | 6-ring | 5-ring | TRP 212 (B) | pi-pi | 4.31 |  |
|  | 6-ring | 6-ring | TRP 212 (B) | pi-pi | 3.93 |  |
|  |  |  |  |  |  |  |
| 18 | N 23 | OH | TYR 258 (B) | H-acceptor | 2.98 | -1.6 |
| 19 | S 11 | OG | SER 250 (B) | H-donor | 3.08 | -0.5 |
|  | O 16 | OH | TYR 249 (B) | H-acceptor | 2.98 | -0.7 |
|  | 6-ring | 5-ring | TRP 212 (B) | pi-pi | 4.33 |  |
|  | 6-ring | 6-ring | TRP 212 (B) | pi-pi | 3.95 |  |
| Chlorpyrifos | S 4 | CB | TRP 212 (B) | H-acceptor | 3.52 | -0.8 |
|  | S 4 | OH | TYR 460 (B) | H-acceptor | 4.27 | -0.8 |
|  | O 6 | OH | TYR 249 (B) | H-acceptor | 3.02 | -0.6 |
|  | 6-ring | 5-ring | TRP 212 (B) | pi-pi | 3.97 |  |

| **Table S3.** 2D interaction profiles of the 19 synthesized compounds targeting the nicotinic acetylcholine receptor (nAChR) in *Culex pipiens*, compared with three conventional nAChR agonists (neonicotinoids): thiamethoxam, clothianidin, and imidacloprid | |
| --- | --- |
| **1** | **2** |
| **3** | **4** |
| **5** | **6** |
| **7** | **8** |
| **9** | **10** |
| **11** | **12** |
| **13** | **14** |
| **15** | **16** |
| **17** | **18** |
| **19** | **Thiamethoxam** |
| **Clothianidin** | **Imidacloprid** |

| Table S4. Molecular docking interaction profiles of 19 synthesized compounds with the nicotinic acetylcholine receptor (nAChR) in *Culex pipiens*, showing ligand–receptor interacting atoms, interacting residues, interaction types, hydrogen bond distances, and binding energies (E, kcal/mol). Data are compared with three conventional nAChR agonists (neonicotinoids): thiamethoxam, clothianidin, and imidacloprid | | | | | | |
| --- | --- | --- | --- | --- | --- | --- |
| Ligand | **Ligand interacting atoms** | **Receptor Interacting atoms** | **Receptor Interacting Residues** | **Interaction Bond Type** | **Distance** | **E (kcal/mol)** |
| 1 | O 11 | CE | LYS 78 (A) | H-acceptor | 3.32 | -0.7 |
|  | S 20 | CB | ALA 76 (A) | H-acceptor | 3.88 | -0.7 |
|  | S 20 | CA | MET 156 (A) | H-acceptor | 3.7 | -1.0 |
|  | S 20 | N | CYS 157 (A) | H-acceptor | 3.82 | -4.0 |
| 2 | S 14 | CA | THR 124 (A) | H-acceptor | 3.95 | -1.1 |
|  | S 14 | N | PHE 125 (A) | H-acceptor | 3.34 | -2.2 |
|  | S 14 | N | ASP 126 (A) | H-acceptor | 3.56 | -4.3 |
| 3 | S 14 | N | CYS 157 (A) | H-acceptor | 4.25 | -0.5 |
| 4 | S 20 | N | CYS 157 (A) | H-acceptor | 4.37 | -1.8 |
|  | 6-ring | CB | SER 295 (A) | pi-H | 4.32 | -0.5 |
|  | 5-ring | CE1 | PHE 435 (A) | pi-H | 3.85 | -0.5 |
| 5 | N 16 | O | VAL 77 (A) | H-donor | 2.92 | -2.5 |
|  | N 16 | O | CYS 157 (A) | H-donor | 2.92 | -2.5 |
|  | N 24 | CA | LYS 78 (A) | H-acceptor | 3.32 | -0.6 |
|  | N 24 | CA | MET 156 (A) | H-acceptor | 3.59 | -0.6 |
| 6 | N 16 | O | VAL 77 (A) | H-donor | 2.9 | -2.4 |
|  | N 16 | O | CYS 157 (A) | H-donor | 3.04 | -2.2 |
|  | N 24 | CA | LYS 78 (A) | H-acceptor | 3.36 | -0.6 |
|  | N 24 | CA | MET 156 (A) | H-acceptor | 3.61 | -0.6 |
|  | 6-ring | CD | LYS 78 (A) | pi-H | 4.68 | -0.5 |
| 7 | N 20 | O | CYS 157 (A) | H-donor | 3.17 | -3.3 |
|  | N 24 | N | ASP 297 (A) | H-acceptor | 3.4 | -1.3 |
|  | 6-ring | NZ | LYS 434 (A) | pi-cation | 4.6 | -0.9 |
| 8 | S 12 | N | CYS 157 (A) | H-acceptor | 3.65 | -3.6 |
| 9 | N 13 | O | CYS 157 (A) | H-donor | 2.96 | -3.0 |
|  | 6-ring | CA | ASP 160 (A) | pi-H | 4.52 | -0.5 |
| 10 | O 10 | N | VAL 77 (A) | H-acceptor | 2.94 | -2.2 |
|  | N 16 | N | CYS 157 (A) | H-acceptor | 3.36 | -0.6 |
| 11 | O 10 | N | CYS 157 (A) | H-acceptor | 3.25 | -2.3 |
|  | S 12 | N | VAL 77 (A) | H-acceptor | 4.43 | -0.8 |
|  | 6-ring | CB | PRO 154 (A) | pi-H | 3.91 | -0.6 |
| 12 | N 13 | O | CYS 157 (A) | H-donor | 3.13 | -3.2 |
|  | O 10 | N | CYS 157 (A) | H-acceptor | 2.99 | -2.8 |
|  | 6-ring | CE | MET 156 (A) | pi-H | 3.99 | -0.5 |
| 13 | O 10 | N | CYS 157 (A) | H-acceptor | 3.49 | -0.9 |
|  | 6-ring | CB | PRO 154 (A) | pi-H | 3.8 | -0.6 |
| 14 | N 13 | O | VAL 77 (A) | H-donor | 3.15 | -2.0 |
|  | N 13 | O | CYS 157 (A) | H-donor | 3.18 | -1.2 |
| 15 | S 12 | CA | THR 124 (A) | H-acceptor | 4.39 | -0.7 |
|  | S 12 | N | PHE 125 (A) | H-acceptor | 3.47 | -2.5 |
|  | S 12 | N | ASP 126 (A) | H-acceptor | 3.54 | -2.9 |
|  | O 10 | NZ | LYS 78 (A) | Ionic | 3.62 | -1.5 |
|  | 5-ring | CG | LYS 78 (A) | pi-H | 3.73 | -1.0 |
| 16 | N 20 | O | CYS 157 (A) | H-donor | 2.91 | -1.0 |
| 17 | S 11 | O | CYS 157 (A) | H-donor | 3.83 | -0.7 |
|  | 5-ring | CE1 | PHE 435 (A) | pi-H | 3.66 | -0.5 |
| 18 | S 18 | O | CYS 157 (A) | H-donor | 4.1 | -0.7 |
|  | N 20 | O | CYS 157 (A) | H-donor | 3.13 | -2.7 |
| 19 | O 16 | N | CYS 157 (A) | H-acceptor | 3.2 | -2.2 |
|  | 6-ring | CB | PRO 154 (A) | pi-H | 3.86 | -0.6 |
|  | 6-ring | CA | SER 158 (A) | pi-H | 4.15 | -0.9 |
| Thiamethoxam | O4 | N | CYS 157 (A) | H-acceptor | 3.35 | -0.8 |
|  | N9 | CA | THR 124 (A) | H-acceptor | 3.61 | -0.5 |
| Clothianidin | O3 | N | CYS 157 (A) | H-acceptor | 3.46 | -0.5 |
| Imidacloprid | O2 | N | CYS 157 (A) | H-acceptor | 2.88 | -2.8 |


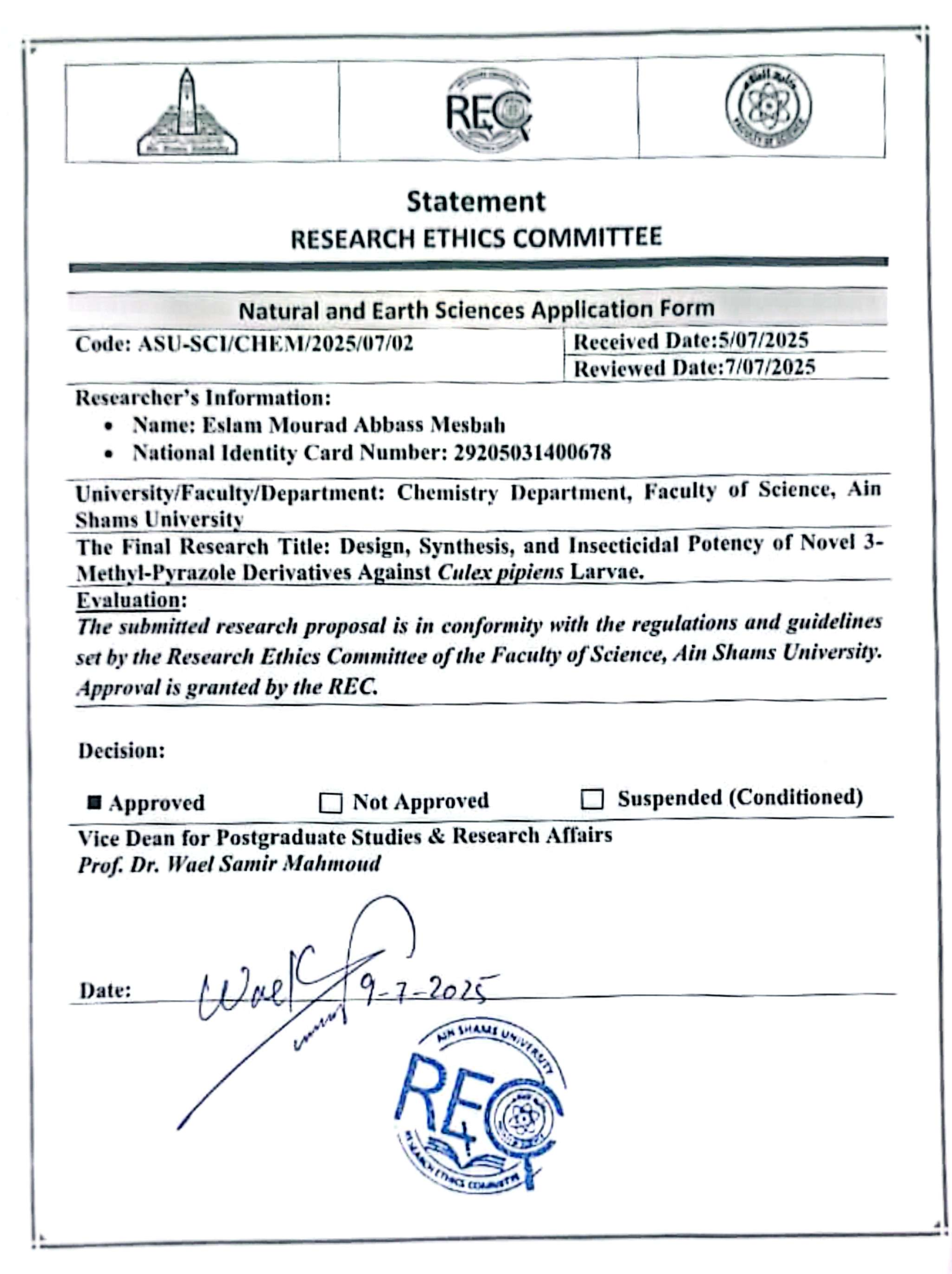

Supplement: Supplementary file 1 — Supplementary Material 1 [file 41598_2026_50895_MOESM1_ESM.docx]
